# Complex assembly and activity states as multifaceted protein attributes explaining phenotypic variability

**DOI:** 10.1038/s44320-026-00228-3

**Published:** 2026-07-22

**Authors:** George Rosenberger, Peng Xue, Isabell Bludau, Claudia Martelli, Evan Williams, Ben C Collins, Andrea Califano, Yansheng Liu, Ruedi Aebersold

**Affiliations:** 1 Department of Systems Biology, Columbia University Irving Medical Center, New York, NY USA; 2 Institute of Molecular Systems Biology, Department of Biology, ETH Zurich, Zurich, Switzerland; 3 Department of Medicine, Columbia University Irving Medical Center, New York, NY USA; 4 Department of Biochemistry & Molecular Biophysics, Columbia University Irving Medical Center, New York, NY USA; 5 Herbert Irving Comprehensive Cancer Center, Columbia University Irving Medical Center, New York, NY USA; 6 Department of Biomedical Informatics, Columbia University Irving Medical Center, New York, NY USA; 7 Biohub New York, New York, NY USA; 8 Yale Cancer Biology Institute, Yale University, West Haven, CT USA; 9 Department of Pharmacology, Yale University School of Medicine, New Haven, CT USA; 10 Department of Biomedical Informatics & Data Science, Yale University School of Medicine, New Haven, CT 06510 USA; 11 Present Address: Bruker Switzerland AG, Fällanden, Switzerland; 12 Present Address: Guangzhou National Laboratory, Guangzhou, China; 13 Present Address: Department of Neuropathology, University Hospital Heidelberg, Heidelberg, Germany; 14 Present Address: Luxembourg Centre for Systems Biomedicine, University of Luxembourg, Esch-Belval, Luxembourg; 15 Present Address: School of Biological Sciences, Queen’s University of Belfast, Belfast, UK

**Keywords:** Proteomics

## Abstract

The state of a cell depends not only on protein abundance, but also on the biochemical and cellular activities of proteins, which are largely invisible to abundance profiling alone. Here, we introduce a multi-omics framework that infers context-specific protein activities from transcriptomic, phosphoproteomic, and protein correlation-based protein-protein interaction data, integrating modality-specific algorithms via network diffusion. Applying it to a panel of phenotypically diverse HeLa cell lines, whose genetic drift provides a natural perturbation system, we make three findings. First, physical separation of monomeric and assembled protein fractions by protein correlation profiling provides direct evidence that complex assembly buffers variation in gene copy number and transcription, a mechanism previously only inferred from bulk measurements. Second, using *Let7* perturbation data, CRISPR gene dependency scores, and subcellular localization, we orthogonally validate that inferred protein activities capture functional regulation linked to cellular phenotypes inaccessible from abundance data alone. Third, differential analysis of context-specific activity profiles identifies molecular mechanisms underlying phenotypic divergence, including a WIPF1/WIPF2--Arp2/3 axis governing invadopodium formation and infection susceptibility, and an immunoproteasome switch linked to immune adaptation.

## Synopsis

A multi-omics framework infers protein activities from co-fractionation, phosphoproteomic, and transcriptomic data. Applied to a panel of HeLa cell lines, it reveals how protein complex assembly buffers genetic variation and drives phenotypic divergence.

- Physical separation of monomeric and assembled protein fractions by SEC-SWATH-MS provides direct evidence that complex assembly buffers gene copy number and transcriptional variation.
- Orthogonal validation using CRISPR gene dependency scores, subcellular localization data, and *Let7* perturbation responses confirms that inferred protein activities capture functional regulation inaccessible from abundance data alone.
- Network diffusion across three complementary activity layers (protein complex dynamics, kinase/phosphatase signaling, and transcription factor activity) identifies a WIPF1/WIPF2–Arp2/3 cytoskeletal axis governing invadopodium formation and *Salmonella* infection susceptibility.
- Differential proteasome complex stoichiometry reveals an immunoproteasome switch linked to immune adaptation and antigen presentation.

## Abstract (web summary)

A multi-omics framework infers protein activities from co-fractionation, phosphoproteomic, and transcriptomic data. Applied to a panel of HeLa cell lines, it reveals how protein complex assembly buffers genetic variation and drives phenotypic divergence.

## Graphical abstract

**Figure:**
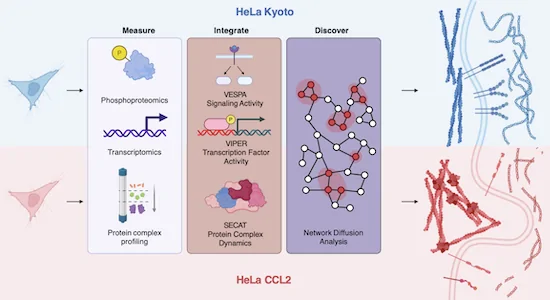

**Figure:**
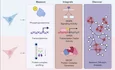

## Introduction

Phenotypes arise from the complex biochemical states of cells and tissues. These states, in turn, reflect the interplay of genes, transcripts, proteins, lipids, and metabolites, a network intricately woven by the central dogma of molecular biology (Crick, 1970). Understanding how phenotypes connect to the underlying molecular landscapes and the molecular mechanisms they constitute remains a fundamental pursuit in both basic and biomedical research. The advent of systems biology (Chuang et al, 2010) and the rapid evolution of molecular profiling techniques, especially multi-omics technologies, offer an unprecedented opportunity for the holistic exploration of biological systems. However, while multi-omics profiling technologies become more accessible, the interpretation and integration of the associated molecular profiles they generate remains challenging.

Many data integration methods struggle with the need to identify causal rather than associational links between molecular abundances and cell function (Dugourd and Saez-Rodriguez, 2019; Pfister et al, 2021; Williams et al, 2022). Latent factor models such as MOFA (Argelaguet et al, 2018) can identify shared variance across omics layers but primarily capture associational patterns. Causal reasoning frameworks such as CARNIVAL (Dugourd et al, 2021) and its generalization CORNETO (Rodriguez-Mier et al, 2025) go further by using optimization to select directed signaling paths through prior knowledge networks that are consistent with measured omics data; however, because the underlying network topology is drawn from precompiled repositories, these approaches are constrained to interactions represented in the reference and may not capture context-specific network rewiring. Crucially, none of these frameworks resolves protein-level structural processes, such as complex assembly and stoichiometry changes, that link molecular abundances to cell function. In particular, the role of proteins, essential in controlling biological processes, exemplifies this complexity, as their function and activities depend on intricate mechanisms of molecular state-dependent interactions.

To address these challenges, we and others have developed network-based approaches that integrate prior and de novo knowledge, including generalized or context-specific molecular interaction networks generated by computational analysis of large-scale data repositories or using experimental techniques (Dugourd and Saez-Rodriguez, 2019). Validated uses of such approaches include the assessment of transcription factor (TF) and co-factor (co-TF) activity from gene regulatory networks, as exemplified by the Virtual Inference of Protein activity using Enriched Regulon analysis, VIPER (Alvarez et al, 2016), or the assessment of kinase/phosphatase activity using signal transduction networks and phosphoproteomic profiles, as exemplified by algorithms such as kinase-substrate enrichment analysis (KSEA (Casado et al, 2013)), PHOsphorylation NEtworks for Mass Spectrometry (PHONEMeS (Gjerga et al, 2021)), or virtual enrichment-based signaling protein-activity analysis (VESPA (Rosenberger et al, 2024)). To further extend the ability of these methods to build quantitative, mechanistic models of cell behavior (Diss, 2020), we recently developed a high-resolution protein co-elution profiling approach (SEC-SWATH-MS (Heusel et al, 2020, 2019)) and the size-exclusion chromatography algorithmic toolkit (SECAT (Rosenberger et al, 2020)). These tools assess context-specific protein network states using metrics that represent protein complex abundance and stoichiometry based on quantitatively characterized protein–protein interactions (PPIs). SECAT’s focus on contextual protein network state, rather than static protein complexes, allows capturing dynamic state/condition-dependent changes in protein complex formation and stoichiometry, making it ideally suited to characterize protein complex dynamics from multi-omics profiles.

In this study, we explore multiomic, network-based inference of protein activity as a more comprehensive approach to unravel phenotype-associated molecular mechanisms, compared to direct transcriptomic or proteomic abundance analysis, or single-layer omics-based network analysis (Box 1). We leveraged previously generated data on the molecular and phenotypic heterogeneity of 14 HeLa cell lines gathered from 13 laboratories (Liu et al, 2019), for which matched genomic, transcriptomic, proteomic, and quantitative phenotypic data (cell doubling time and *Salmonella* infection susceptibility) are available. Among these, the HeLa CCL2 variant is the more invasive and infection-susceptible subline, whereas the HeLa Kyoto variant exhibits faster doubling time (Liu et al, 2019).

This panel represents a “natural perturbation” system where genetic drift has generated a diverse landscape of genotypes, proteotypes (i.e., the contextual state of the proteome encompassing protein abundance, post-translational modifications, and interaction states), and phenotypes, providing an ideal testbed for evaluating genotype–phenotype relationships along the axis of the central dogma. In the original study, cells were subjected to multi-omics profiling, including the transcriptome, proteome, and protein turnover. However, protein complex profiling was not performed, leaving open how complex assembly directly buffers gene dosage variation in this system. In the present study, we extend the multimodal dataset with protein complex profiling data that capture the context-specific protein interaction networks in phenotypically different cell lines. Using this extended dataset, we tested the following three hypotheses: First, protein complex stoichiometry represents an effective buffering mechanism against copy number (CNV) and transcriptional heterogeneity. Second, protein functional state is more closely related to molecular mechanisms and cellular phenotypes than protein abundance. Third, the integration of multiple omics layers widens the information that connects molecular profiles and phenotypes.

Our study demonstrates the importance of characterizing context-specific, dynamic protein complex composition as an essential element of the proteotype and the potential of multi-layer network integration to gain new insights into the molecular mechanisms underlying cellular phenotypes.

Box 1 Integrated multi-omics toolkit for inferring protein functional states To move beyond protein abundance as a proxy for function, we employ a suite of network-based algorithms that infer specific “activity” states from distinct omics layers. In this context, protein activity refers to the functional output of a protein, whether it is catalytic (signaling), regulatory (transcriptional), or structural (complex assembly).

- VIPER (Virtual inference of protein activity by enriched regulon analysis): Infers gene regulatory activity (transcription factor and co-regulator activity) from transcriptomic data (Alvarez et al, 2016). VIPER models a regulator’s activity as the enrichment of its transcriptional targets (its regulon) among the most differentially expressed genes in a given sample. Regulons are typically derived from context-specific gene regulatory networks reconstructed by ARACNe (Margolin et al, 2006), enabling sample-tailored inference rather than reliance on generic pathway databases.
- VESPA (Virtual enrichment-based signaling protein-activity Analysis): Infers signaling activity (kinase/phosphatase activity) from phosphoproteomic data, extending the VIPER enrichment principle to the post-translational layer (Rosenberger et al, 2024). It builds a curated kinase–substrate network and estimates the activity of each kinase or phosphatase based on the enrichment of its downstream phosphorylation targets among the most differentially phosphorylated sites.
- SECAT (Size-exclusion chromatography algorithmic toolkit): Infers protein complex assembly state from SEC-SWATH-MS data. By analyzing the co-elution profiles of interacting proteins, SECAT quantifies complex composition, stoichiometry and abundance, distinguishing between monomeric (often inactive or buffered) and assembled (functional) pools (Rosenberger et al, 2020).
- Network diffusion analysis: Integrates these distinct layers by modeling information flow over a protein–protein interaction network. diffuStats (Picart-Armada et al, 2018), the specific method used here, uses a random-walk-based diffusion approach to propagate molecular signals across the network, finding relevant pathways linking upstream perturbations (e.g., signaling activity) to downstream effects (e.g., complex state changes).

## Results

### A systems workflow measuring, inferring, and comparing protein activities and complex states

**Figure 1:**
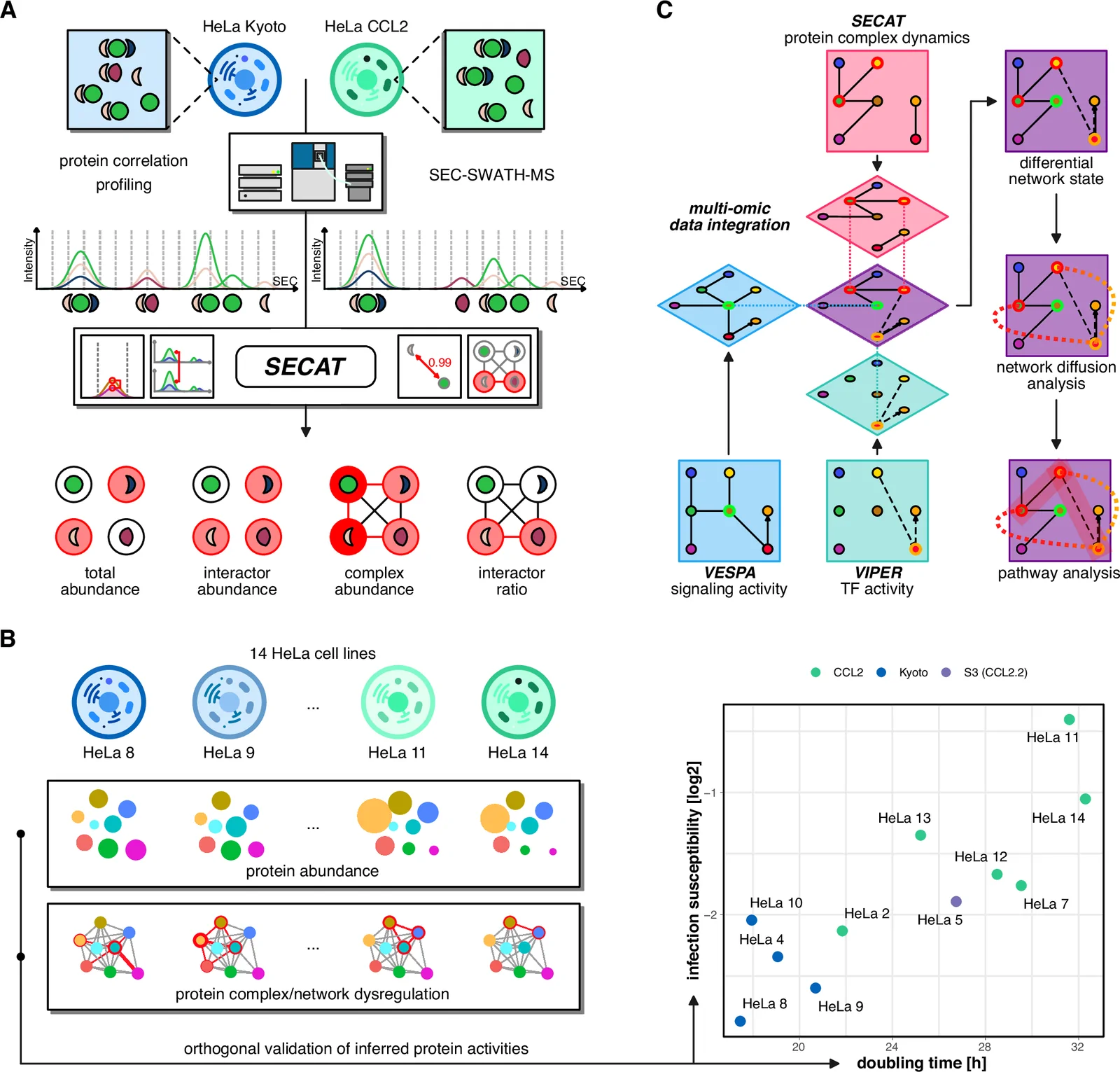
Methodological overview. (**A**) Protein complex profiles were generated by SEC-SWATH-MS and then analyzed using the size-exclusion chromatography algorithmic toolkit (SECAT). SECAT uses a STRING protein–protein interaction network (PPIN) as a reference to generate a context-specific network and to infer protein attributes for complex abundance and stoichiometry. (**B**) The HeLa cell lines used in the study were characterized by their protein abundance patterns and inferred network states (VIPER, VESPA, and SECAT). Correlation of these molecular profiles with phenotypic traits and Let7 microRNA perturbation responses across the panel served as orthogonal validation that the inferred protein activities capture biologically relevant variation. (**C**) Differential transcription factor (TF) activity was inferred using VIPER, differential signaling activity was inferred using VESPA, and protein complex attributes were obtained from the SECAT analysis. The combined results provide an overview of the connectivity of proteins and their multimodal differences between the two HeLa subtypes Kyoto and CCL2. Network diffusion analysis makes use of the context-specific protein–protein interaction network (PPIN), kinase-substrate network (KSN), and gene regulatory network (GRN) to assess the effects of upstream alterations on downstream layers.

We describe a workflow that comprises three components (see Fig. 1 for an overview). First, we deployed an experimental component to investigate the buffering effects of protein complex stoichiometry against copy number variation (CNV) and transcriptional variation. We measured the two most distant instances of the 14 tested HeLa Kyoto and CCL2 cell lines by SEC-SWATH-MS to obtain high-resolution protein complex profiles (Fig. 1A). Quantitative assessment of the effects of CNV and mRNA abundance on monomeric and assembled protein complex subunits allowed us to draw generalized conclusions about the effects of abundance buffering for monomeric and complexed proteins on the functional cellular state.

Second, we developed and applied a multiomic, network-based approach to infer protein activities and complex states. To ask whether these inferred activities and detected complex states capture biologically relevant mechanisms beyond the level apparent from protein abundance alone, we performed validation using three orthogonal datasets (Fig. 1B): CRISPR gene dependency scores from the DepMap portal (Tsherniak et al, 2017; Data ref: Tsherniak et al, 2017), subcellular localization data from the Human Protein Atlas (HPA) (Thul and Lindskog, 2018; Data ref: Thul and Lindskog, 2018), and the *Let7* microRNA perturbation data from the original 14-cell-line panel (Liu et al, 2019). Together, these ground-truth references demonstrate that inferred activities prioritize essential genes, map to coherent subcellular structures, and are predictive of phenotypic outcomes.

Third, we explored how protein activities and complex states support meaningful biological comparisons. We conducted a differential comparison of HeLa CCL2 vs. Kyoto using the inferred protein activities of VESPA, VIPER, and SECAT (Fig. 1C). A network diffusion approach elucidated the functional dependencies between the two cell lines, ranging from altered signaling and transcriptional programming to reorganization of the protein complexes and macromolecular structures involved in the organization of the extracellular matrix, which are directly responsible for previously observed phenotypes.

### Quantitative effects of copy number variation and mRNA abundance on protein complex assembly state

The assembly state of cellular proteins broadly impacts their physiological function and cellular phenotypes. Previous work has shown that ~60% of protein mass in cell extracts is observed in complexed form (Heusel et al, 2019). Multiple layers of regulation contribute to maintaining protein complex stoichiometry: coordinated translational control can tune subunit production rates (Taggart et al, 2020), while orphaned subunits that fail to assemble are targeted for degradation by the ubiquitin-proteasome system (Juszkiewicz and Hegde, 2018; Pla-Prats and Thomä, 2022). The relative contributions of these mechanisms depend on the biological context: for instance, gain-type aneuploidy is primarily buffered by accelerated degradation of excess subunits, whereas loss-type aneuploidy seems to engage selective upregulation of protein synthesis (Di et al, 2025), establishing a multi-layered buffering system against gene dosage variation (Liu et al, 2019, 2016). However, previous studies largely inferred this buffering from global correlation analyses, pulsed-SILAC measurements, or bulk protein pools (Salovska et al, 2020). Here, we provide direct physical evidence for the complex-based buffering hypothesis by resolving the distinct behavior of monomeric versus assembled fractions of the same proteins.

**Figure EV1:**
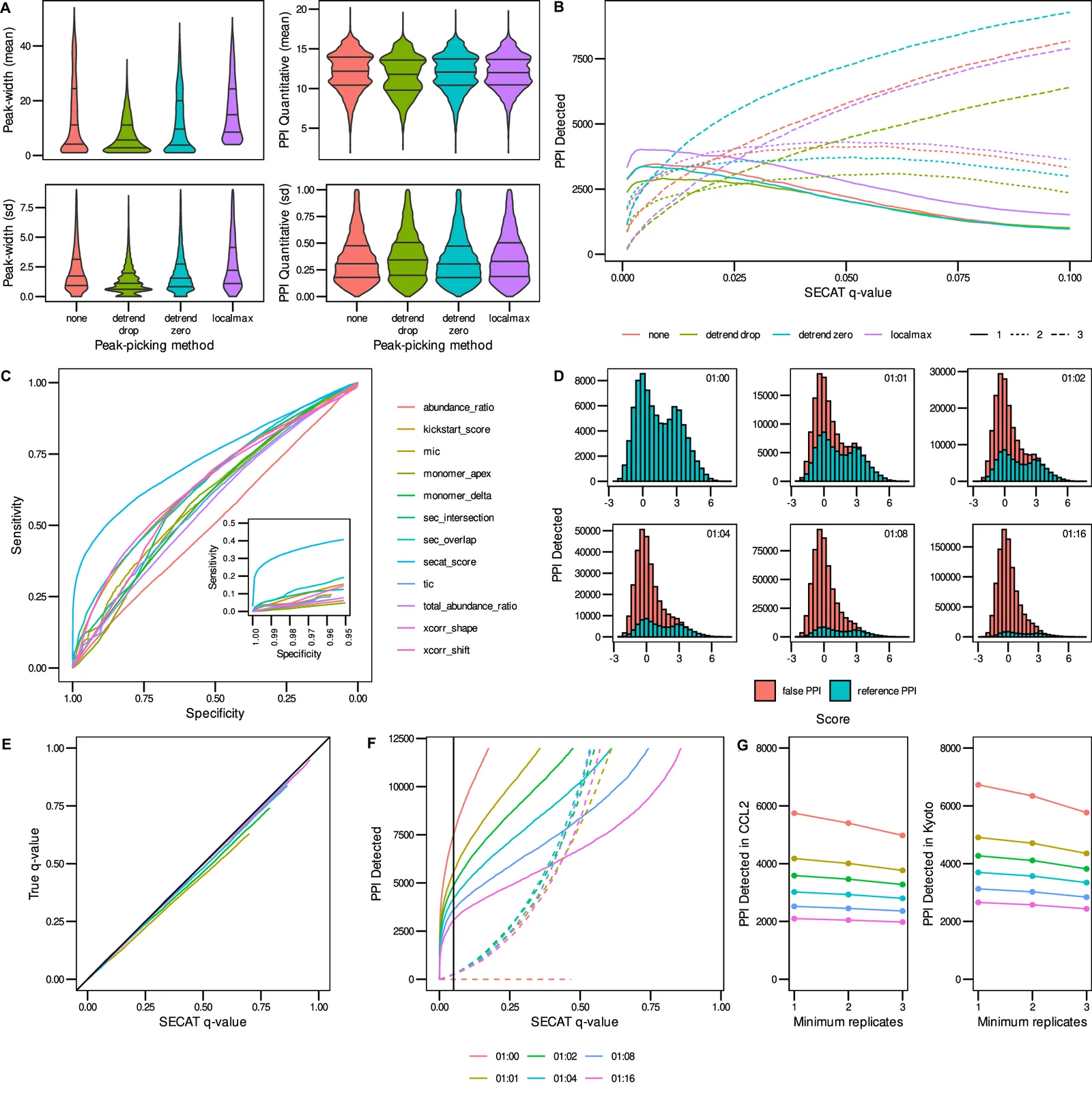
SECAT signal processing and PPI detection performance. (**A**) The effect of different peak-picking methods for signal processing on peak-width and PPI quantification within replicates of the same experimental condition is depicted in boxplots (center line, median; box limits, first and third quartiles; whiskers, 1.5× IQR). (**B**) Sensitivity of PPI detection vs. SECAT *q* value for peak-picking options. The PPI were filtered to a global context q-value < 0.05. Colors indicate different peak-picking options; line types indicate the number of detections with the three replicates per condition. (**C**) The receiver-operating characteristic (ROC) illustrates the sensitivity vs. specificity of the different SECAT subscores, the kickstart score and the integrated SECAT score. (**D**) The SECAT score histograms depict the dilution of the reference PPI network (true: CORUM) with false PPI (false: CORUM-inverted) for the benchmark (Methods). (**E**) The SECAT estimated q-value is accurate as evaluated against the ground truth for all levels of reference PPI network accuracy. (**F**) The sensitivity of PPI detection in dependency of SECAT *q* value for different levels of reference PPI network accuracy is depicted. (**G**) Consistency of PPI detection between replicates of the same conditions. All panels: n=3 biological replicates per cell line (HeLa CCL2 and HeLa Kyoto).

**Figure EV2:**
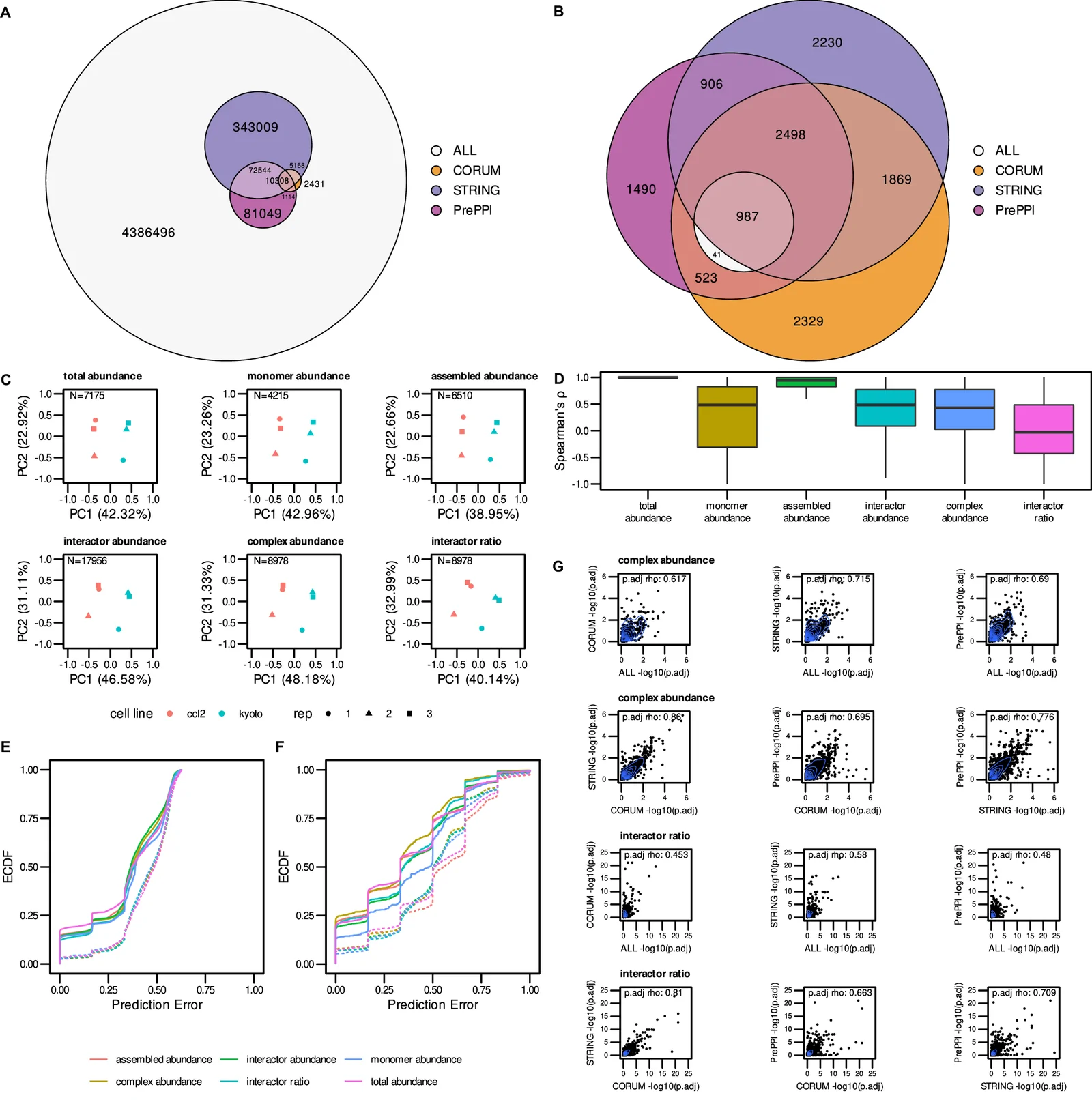
SECAT PPI quantification and Network integration. (**A**) The Euler diagram indicates the overlap of STRING, PrePPI and CORUM reference PPI networks, resulting in at least a partial overlap of the interactors in any of the replicates. (**B**) Overlap of confident (*q* value <0.05) PPI detections after SECAT analysis with different reference PPI networks. (**C**) Quantitative SECAT features can be used to separate experimental conditions and replicates. (**D**) Spearman’s correlation of features (PPI: summarized by averaging) to total-level features is depicted in individual boxplots (center line, median; box limits, first and third quartiles; whiskers, 1.5× IQR). (**E**) Predictive error for feature-wise leave-one-out cross-validated logistic regression classification of replicates to biological conditions. (**F**) Predictive error for group-wise leave-one-out cross-validated logistic regression classification of replicates to biological conditions. Groups represent several features connected to a core feature based on the PPI network. (**G**) Correlation of node-level integrated BH-adj. p-values estimated using different reference PPI networks. Spearman’s rank correlation coefficient (ρ) is depicted, with statistical significance evaluated using a two-sided Spearman’s rank correlation test. All panels: n=3 biological replicates per cell line (HeLa CCL2 and HeLa Kyoto).

We applied our SEC-SWATH-MS approach (Heusel et al, 2019) and the SECAT algorithm (Rosenberger et al, 2020) to physically separate and quantify these assembly states in representative HeLa Kyoto and HeLa CCL2 cell lines. From 420 LC-MS/MS runs across three replicates per cell line, we generated a quantitative profiling matrix comprising 7175 proteins (1% global context FDR). SECAT inferred context-specific protein–protein interaction (PPI) networks and derived six quantitative metrics per protein: total abundance, assembled (complex-bound) abundance, monomer abundance, complex abundance, interactor abundance, and interactor ratio (Figs. 1A and EV1 and EV2; Dataset EV1).

**Figure 2:**
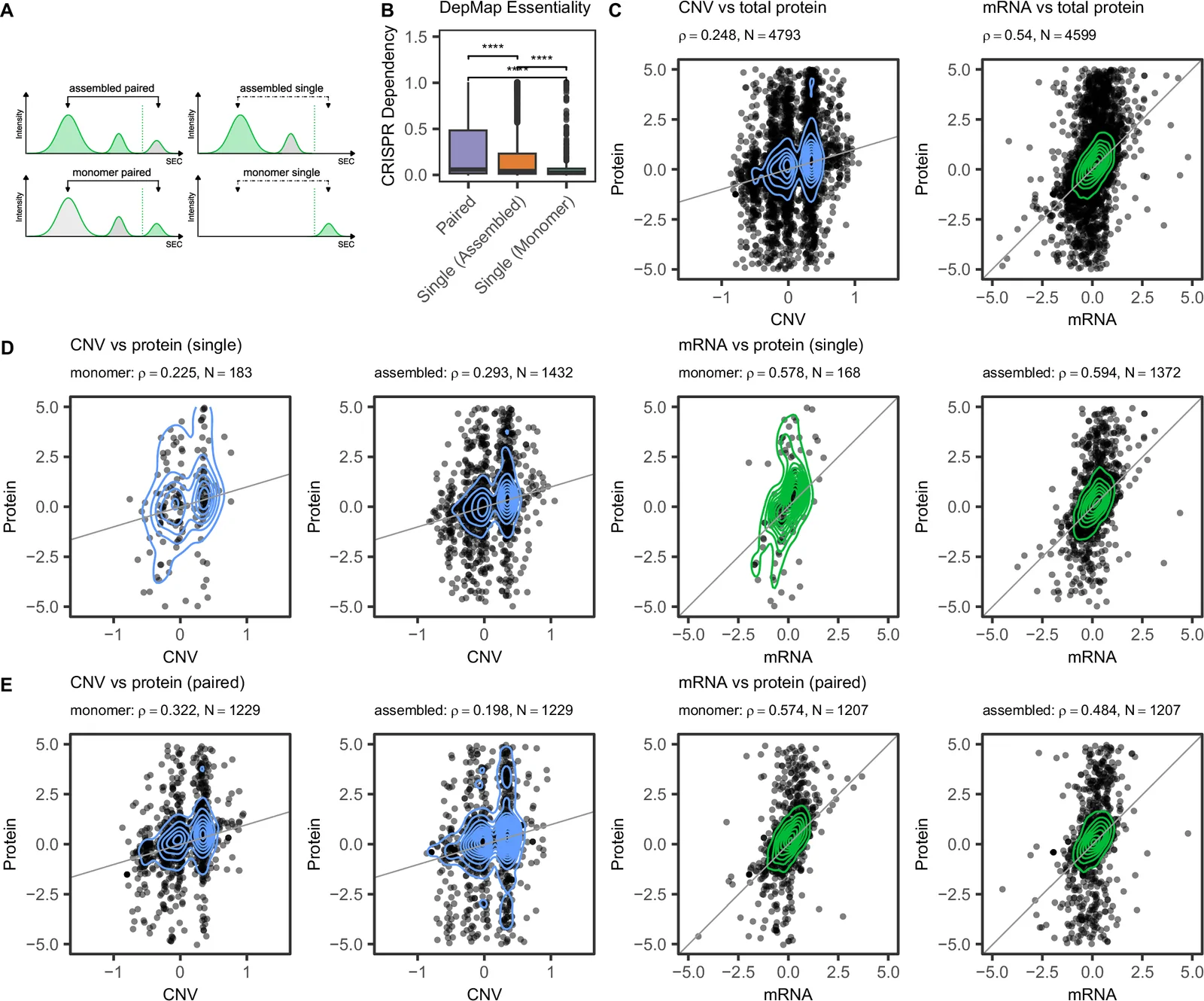
Dependency of monomer and assembled levels on copy number variation (CNV) and mRNA abundance. (**A**) Definition of paired and single monomer and assembled protein fractions. (**B**) DepMap gene essentiality analysis reveals that “paired state” proteins (buffered in assembly) are significantly more essential than “single monomer” proteins ($p=1.35\times {10}^{-29}$, Wilcoxon rank-sum test; median dependency probability: paired 0.065, single assembled 0.048, single monomer 0.032; ${n}_{\mathrm{paired}}=\mathrm{3,439}$; ${n}_{\text{single assembled}}=\mathrm{2,869}$; ${n}_{\text{single monomer}}=654$), supporting the functional shielding hypothesis. Boxplots: center line, median; box limits, first and third quartiles; whiskers, 1.5× interquartile range. (**C**) CNV and mRNA abundance vs. total protein abundance (CNV: $n=\mathrm{4,793}$; mRNA: $n=\mathrm{4,599}$ proteins with complete data). (**D**) CNV and mRNA abundanc**e** vs. single-state monomeric (CNV: n=183; mRNA: n=168) and assembled (CNV: $n=\mathrm{1,432}$; mRNA: $n=\mathrm{1,372}$) protein abundance. (**E**) CNV and mRNA abundance vs. paired monomeric and assembled protein abundance (CNV: $n=\mathrm{1,229}$; mRNA: $n=\mathrm{1,207}$ protein pairs with complete data). Panels (**C**–**E**) show Spearman’s *ρ* correlation coefficients; n=3 biological replicates per cell line. Significance of correlation differences was assessed by Fisher’s transformation (Methods). Source data are available online for this figure.

We integrated the fraction-specific capabilities of our framework with cell line-matched CNV and mRNA abundance data (Liu et al, 2019) (Fig. 2; Dataset EV2). While total protein abundance correlated with CNV and mRNA as previously reported (Spearman’s ρ: 0.248 and 0.540, respectively; original study: Spearman’s ρ: 0.217 and 0.510, Fig. 2C), the quantitative SECAT metrics provided direct evidence for monomer buffering when decoupling proteins into “single state” (exclusively monomeric or assembled) and “paired state” (present in both conformations) (Fig. 2A).

To interpret these results, it is important to consider the limitations and strengths of each comparison. “Single state” proteins (those detected exclusively as monomeric or assembled) may be subject to confounding factors such as differences in group size, signal-to-noise, and protein biological heterogeneity between monomeric or assembled groups. In contrast, the “paired state” design (proteins present in both monomeric and assembled forms) provides a robust internal control: both fractions are derived from the same protein in the same sample, so translational and environmental factors are matched, and the number of observations is balanced. This enables a more controlled and interpretable comparison of gene dosage effects on monomeric versus assembled fractions.

With that in mind, we compared the correlation of monomeric and assembled fractions with gene dosage for both groups. For “single state” proteins, monomeric and assembled fractions correlated similarly with CNV and mRNA abundance (no significant difference: monomer/assembled CNV p=0.177; mRNA p=0.383, Methods, Fig. 2D). For “paired state” proteins, the monomeric fractions tracked gene dosage significantly more strongly than their assembled counterparts (monomer/assembled: Spearman’s ρ: 0.322 vs. 0.198 for CNV, p=0.000492, based on 1229 CNV-protein pairs; 0.574 vs. 0.484 for mRNA, p=0.00109, based on 1207 mRNA-protein pairs, Fig. 2E). In this controlled comparison, the observation that monomeric fractions correlate better with gene dosage while assembled fractions of the same proteins tend to be buffered, provides physical evidence that complex assembly insulates subunits from dosage variation: uncomplexed protein subunits lacking cognate binding partners (due to CNV/mRNA variability) are recognized by cellular assembly quality control pathways (Juszkiewicz and Hegde, 2018; Pla-Prats and Thomä, 2022) and subjected to active protein degradation and turnover regulation (Salovska et al, 2020), while the assembled fraction remains stable.

Crucially, we investigated how this structural buffering acts as a mechanism preserving cellular fitness, a core component of our orthogonal validation. Using DepMap CRISPR gene dependency scores (Tsherniak et al, 2017; Data ref: Tsherniak et al, 2017), we found that “paired state” proteins, which exhibit this buffering, are significantly more essential than “single state” monomeric proteins ($p=1.35\times {10}^{-29}$, Wilcoxon rank-sum test; median dependency probability: 0.065 vs. 0.032) (Fig. 2B). This confirms that protein complex assembly functions as a protective shield for core physiological machinery against genetic drift.

By physically resolving protein complex states, we provide empirical evidence of protein complex stoichiometry-driven buffering that historically could only be inferred from steady-state, bulk correlations (Liu et al, 2017, 2019, 2016). This validates the principle that decomposing protein total abundance into distinct interaction and activity states is considerably more informative for deciphering mechanisms of cellular regulation.

### Multiomic inference of differential protein activities and complex states

A core premise of our framework is that protein function depends not only on abundance, but on a protein’s contextual embedding in molecular networks. In a living cell, multiple distinctive networks, specifically transcriptional networks, kinase-substrate, and PPI networks, are concurrently present and collectively contribute to molding the cellular state. To capture these functionally relevant attributes, we performed a comprehensive comparative analysis between HeLa CCL2 and Kyoto cells by deploying our multiomic network algorithms across the respective data layers.

To infer differential kinase/phosphatase activities between the cell lines tested, we applied the multi-sample VESPA (Rosenberger et al, 2024) (msVESPA) algorithm. Using a contextualized signaling network covering 549 kinases and phosphatases and previously published phosphoproteomic profiles of HeLa Kyoto vs. CCL2 cells (Wu et al, 2021) (six cell lines each, measured in triplicates), we identified 73 out of 549 enzymes to be differentially active (BH-adj. *p* value <0.05; Methods, Dataset EV3).

In parallel, we inferred differential transcription factor (TF) activities using multi-sample VIPER (Alvarez et al, 2016) (msVIPER) on previously published transcriptomic profiles (six HeLa Kyoto and ten HeLa CCL2 cells). Utilizing a metaVIPER (Ding et al, 2018) contextualized gene regulatory network comprising 18,926 target genes, msVIPER inferred the activity of 6156 TF/co-TF and signaling proteins, identifying 2009 as differentially active (BH-adj. *p* value <0.05; Methods, Dataset EV4).

Finally, at the structural level, we applied SECAT to the HeLa CCL2 vs. Kyoto SEC-SWATH-MS protein correlation profiles. SECAT identified 702 proteins differing by total abundance, 456 by assembled state abundance, and 281 by monomer abundance. At the level of protein interactions, we identified 412 proteins by differential complex abundance and 479 proteins with different interactor ratios (BH-adj. *p* value <0.05; Dataset EV1).

**Figure 3:**
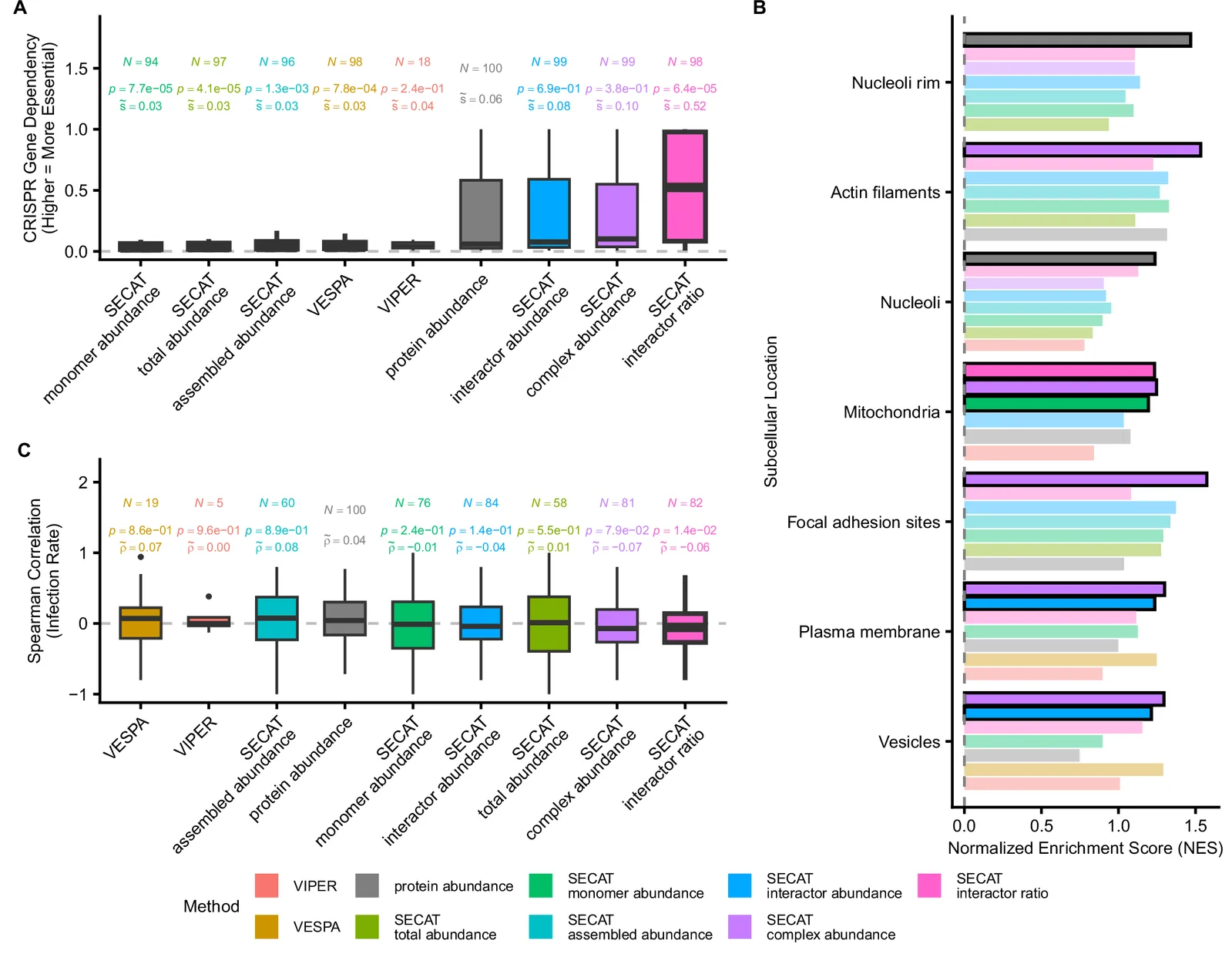
Orthogonal validation of inferred protein activities and complex states. (**A**) Essentiality: Median CRISPR gene dependency scores (DepMap 25Q3, cervical cancer cell lines) for the top 100 proteins per method. SECAT interactor ratio features uniquely map to highly essential genes (median dependency probability 0.52; $p=6.4\times {10}^{-5}$ vs. protein abundance, two-sided Wilcoxon rank-sum test). Boxplots: center line, median; box limits, first and third quartiles; whiskers, 1.5× IQR. (**B**) Subcellular coherence: Normalized enrichment scores (NES; fgsea, 10,000 permutations) against Human Protein Atlas subcellular compartments. Opaque bars with black outline indicate significant enrichment (BH-adj. p<0.05). SECAT complex abundance features are enriched in five compartments: focal adhesion sites, actin filaments, plasma membrane, vesicles, and mitochondria. (**C**) Phenotypic relevance: Per-protein Spearman correlations between Let7-induced abundance changes and Salmonella infection rate across ten HeLa sub-cell lines (n=100 proteins per method from the CCL2 vs. Kyoto baseline comparison). SECAT interactor ratio features show a significantly more negative distribution than protein abundance (p=0.026, two-sided Wilcoxon rank-sum test). Boxplots as in (**A**). Source data are available online for this figure.

We hypothesized that if these functionally relevant attributes reflect mechanistically relevant biology, they should: (i) prioritize genes that are essential for cell fitness, (ii) resolve spatially coherent subcellular structures, and (iii) identify proteins whose response to perturbation tracks with phenotypic outcomes. To test these hypotheses, below we evaluated the top 100 most differentially active proteins per data modality from the CCL2 vs. Kyoto baseline comparison against three independent reference datasets (Fig. 3, Methods). To ensure a systematic comparison, candidates were selected based on their statistical significance: for each omics layer, all proteins were ranked by −log_10_ (BH-adj. *p* value) and the top 100 were retained. This uniform significance-based ranking enabled a fair comparison across data modalities with different effect-size scales.

#### Essentiality: DepMap CRISPR dependency

The most discriminating validation of functionally relevant features emerged from CRISPR-based gene dependency scores derived for cervical cancer cell lines (DepMap 25Q3 (Tsherniak et al, 2017; Data ref: Tsherniak et al, 2017); Fig. 3A). The SECAT “interactor ratio” metric uniquely identified highly essential genes: the top 100 proteins with altered interaction stoichiometry mapped to a median gene dependency probability of 0.52, an 8.7-fold enrichment over all differentially abundant proteins (median 0.06; $p=6.4\times {10}^{-5}$, Wilcoxon rank-sum test). This indicates that changes in protein complex stoichiometry pinpoint core cellular machinery, including ribosomes, proteasomes, or spliceosomes, that is critical for cell viability, yet largely invisible to abundance-level analysis. SECAT “complex abundance” features also showed elevated essentiality (median 0.10), consistent with their structural role. In contrast, VESPA-nominated kinases showed significantly lower essentiality than abundance features (median 0.03; $p=7.8\times {10}^{-4}$), indicating that the signaling differences between CCL2 and Kyoto involve specialized regulatory circuits, rather than indispensable core machinery. SECAT abundance metrics (assembled, monomer, total) similarly identified predominantly non-essential genes, confirming that protein-level abundance differences largely reflect non-functional passenger variation.

#### Spatial coherence: Human Protein Atlas localization

We next assessed whether the nominated proteins resolve to coherent subcellular compartments using fast gene set enrichment analysis (fgsea) against HPA subcellular locations (Thul and Lindskog, 2018; Data ref: Thul and Lindskog, 2018) (Fig. 3B, Methods). SECAT “complex abundance” features showed the broadest range of spatial coherence, with five significantly enriched compartments (Benjamini–Hochberg [BH]-adj. p<0.05): focal adhesion sites, actin filaments, plasma membrane, vesicles, and mitochondria, compartments directly relevant to the cytoskeletal and membrane-trafficking phenotypes differentiating CCL2 and Kyoto cells. SECAT “interactor abundance” was enriched for the categories “plasma membrane” and “vesicles”, while the metric “interactor ratio” was enriched for mitochondria. In contrast, protein abundance features were enriched only for nucleolar proteins, likely reflecting the strong scaling of ribosomal biogenesis factors with proliferation rate (Kyoto cells have a faster doubling time) rather than specific mechanistic rewiring, and neither VESPA nor VIPER features showed significant compartment enrichment. The spatial specificity of SECAT complex metrics confirms the physical validity of the inferred complex perturbations.

#### Phenotypic relevance: *Let7* perturbation

Having established the general validity of these metrics against global reference datasets, we next asked if they could explain specific, complex phenotypic responses in our perturbation model. In our previous study (Liu et al, 2019), *Let7* microRNA mimics were transfected into ten HeLa sub-cell lines, and proteome-wide abundance ratios between *Let7*-treated and control cells were quantified by SWATH-MS. *Let7* is a conserved tumor suppressor microRNA whose expression is generally downregulated upon *Salmonella* Typhimurium infection of HeLa cells. *Let7* overexpression reduced *Salmonella* infection susceptibility in most tested cell lines, but with substantial inter-line variability linked to the underlying proteotype (Liu et al, 2019). For each nominated protein, we computed the Spearman correlation between its *Let7*-induced abundance change and the infection rate across the cell line panel (Fig. 3C). All methods yielded modest median correlations (*|*ρ*|*<0.12), consistent with the distinct molecular origins of the two sources of variation: the baseline proteome comparison captures constitutive, genetic drift-driven proteotype differences, whereas the *Let7* response reflects an acute miRNA perturbation. Within this context, SECAT “assembled abundance” features showed the highest positive median correlation (ρ=0.09), while SECAT “interactor ratio” features showed a significantly more negative distribution (ρ=−0.11; p=0.026 compared to protein abundance, Wilcoxon rank-sum test). This divergence is biologically interpretable: proteins identified by altered complex stoichiometry between cell lines are buffered against the acute *Let7* perturbation, consistent with the structural shielding mechanism established in the preceding section. The substantially larger effect size in the DepMap analysis (Fig. 3A) compared with the more modest Let7 correlations reflects these distinct molecular endpoints: essentiality scores directly probe the structural machinery that SECAT’s interactor ratio metric captures, whereas the Let7 correlation tests cross-perturbation generalizability.

Together, these three orthogonal validation axes of nominating functionally relevant proteins demonstrate that each molecular metric captures a distinct biological facet: the SECAT metric “interaction ratio” identifies essential structural machinery that is buffered against perturbation, “complex abundance” maps to coherent subcellular features relevant to the CCL2–Kyoto phenotypic divergence, and the VESPA metric “signaling activity” highlights specialized, non-essential regulatory rewiring. Critically, protein abundance changes, while readily detectable, are neither sufficiently predictive of perturbation responses, nor significantly enriched for gene dependencies, nor spatially resolved beyond nucleolar proteins. For example, the protein LIMK1, a cytoskeletal regulator central to the invasion phenotype, is detected as differentially active by VESPA but is undetected in the unfractionated total proteome dataset; proteasome subunits such as PSMB8 and PSMB9 are detected at altered complex stoichiometry by SECAT but are not differentially abundant in unfractionated total protein measurements (PSMB8: undetected by the original measurements; PSMB9: log_2_ FC = 0.43, FDR = 0.50). Similarly, the IQGAP2–CDC42 scaffolding complex is differentially abundant between cell lines by SECAT (Fig. 6G), yet neither protein changes significantly in unfractionated total abundance (IQGAP2: log_2_ FC = −0.32, FDR = 0.49; CDC42: log_2_ FC = 0.48, FDR = 0.49); and COP9 signalosome subunits are not significantly changed in unfractionated abundance (all FDR >0.1) while SECAT reveals differential DDB1/2 supercomplex stoichiometries and significant interactor ratio changes across most CSN subunits (Fig. 6H). In each case, the mechanistic insight (proteasome isoform switching (Fig. 6I), scaffolding-dependent GTPase activation, or supercomplex reconfiguration) is invisible to abundance analysis alone. These results establish that network-based inference of protein activity states captures complementary mechanistic information inaccessible from abundance data alone and that the different network types tested identify features active at complementary network layers.

### Synergies between differential kinase/phosphatase, transcription factor activities and protein complex dynamics of HeLa Kyoto vs. CCL2 cell lines

Having validated the robustness of these inferred functional features, we next investigated whether their respective multiomic profiles offer complementary and synergistic insights, surpassing individual contributions, to discern the biological variability between HeLa Kyoto and CCL2 cell lines.

**Figure 4:**
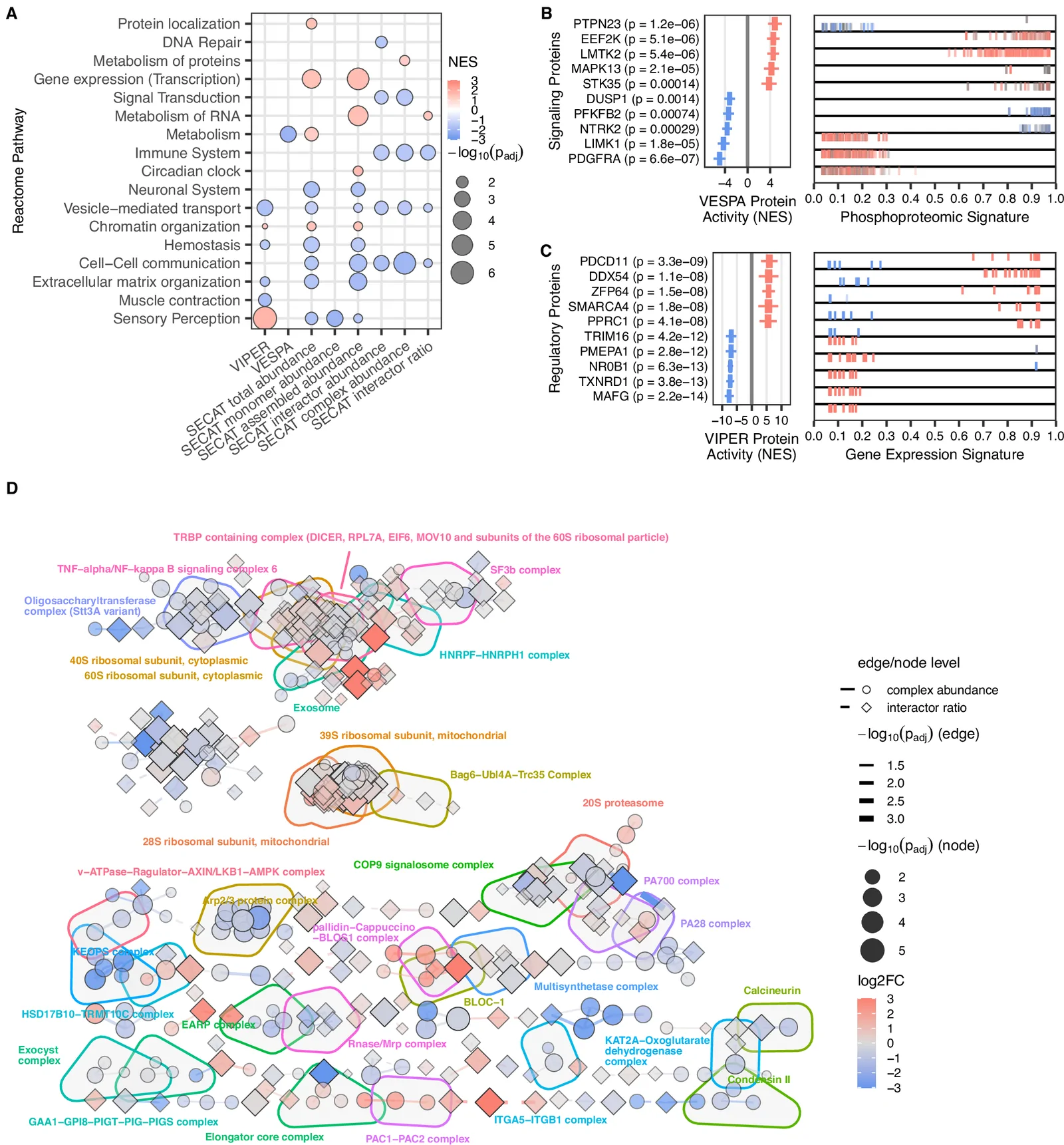
Differential kinase/phosphatase, transcription factor activities and protein complex dynamics of HeLa Kyoto vs. CCL2 cell lines. (**A**) Gene set enrichment analysis (GSEA; fgsea, default parameters) against the Reactome top pathways indicates differences between the cell line subtypes. Nominal enrichment *p* values were estimated using a gene-permutation-based test (10,000 permutations) and adjusted for multiple testing using the Benjamini–Hochberg (BH) method. Dot size indicates $-{\log }_{10}$(BH-adj. *p* value); dot fill indicates NES (blue, negative; salmon, positive). Only pathways with BH-adj. *p* value <0.05 are shown. SECAT: signed sco = sign(log_2_ fold-change) × (−log_10_ BH-adj. *p* value); msVESPA (${n}_{\mathrm{kinases}}=549$; six HeLa Kyoto vs. six HeLa CCL2 cell lines, each measured in triplicates) and msVIPER (${n}_{\mathrm{TF}/\mathrm{co}-\mathrm{TF}}=\mathrm{6,156}$; six HeLa Kyoto vs. ten HeLa CCL2 cell lines): NES. (**B**) The top ten signaling proteins with differential signaling activity between the two conditions are depicted. NES values and statistical significance are analytically estimated using a three-tailed enrichment test (R package viper) with nominal p-values adjusted across all tested kinases via the BH method. Boxplots: center line, median; box limits, first and third quartiles; whiskers, 1.5× IQR; points, individual leading-edge target NES values. (**C**) The top 10 regulatory proteins with differential gene regulatory activity between the two conditions are depicted. NES values and statistical significance are analytically estimated using a three-tailed enrichment test (R package viper) with nominal *p* values adjusted across all tested transcription factors via the BH method. Boxplots as in (**B**). (**D**) STRING-based protein–protein interaction network of proteins with significant changes at the complex abundance or interactor ratio level (BH-adj. *p* value <0.05). Node-level and interaction-level statistical significance (p-values) were estimated using SECAT’s semi-supervised classifier trained on reference complexes, followed by multiple-testing adjustment using the BH method. Node color indicates log_2_ (fold-change) (HeLa Kyoto vs. HeLa CCL2; blue: higher in HeLa CCL2, salmon: higher in HeLa Kyoto). Node size indicates statistical significance (−log_10_ BH-adj. *p* value). Node shape and edge line type distinguish complex abundance (circle/solid line) from interactor ratio (diamond/dashed line) features; CORUM-annotated complex hulls are labeled. SEC-SWATH-MS data: n=3 biological replicates per cell line. Source data are available online for this figure.

Within the msVESPA signaling profiles, Gene Set Enrichment Analysis (GSEA) using the normalized enrichment score (NES) identified metabolic kinases and phosphatases as the only significantly enriched gene set (adj. *p* value <0.05, Fig. 4A). The most differentially active enzymes included a cluster of receptor tyrosine kinases all showing lower activity in Kyoto cells (PDGFRA, NTRK2, NTRK3, TYRO3, MERTK, and FLT1), the cytoskeletal regulator LIMK1, metabolic kinases (EEF2K, PFKFB2, and PRKAA1/2), and the MAP kinase MAPK13 (Fig. 4B).

For the msVIPER regulatory profiles, due to the larger number of nominated proteins, GSEA identified additional statistically significant gene sets compared to the signaling network analysis. These included extracellular matrix organization, vesicle-mediated transport, muscle contraction, hemostasis, chromatin organization and sensory perception (Fig. 4A). Among the most differentially active proteins, the strongest signal converges on the oxidative stress and antioxidant response axis: MAFG (the obligate NRF2 heterodimerization partner) and the canonical NRF2 target genes TXNRD1 and GCLC are all more active in CCL2 cells, pointing to elevated antioxidant programming in the more invasive subline. Additional top regulators include WNT5A (Wnt signaling), RAB33B and RAB23 (vesicular transport), PPP1R12A (myosin phosphatase/cytoskeleton organization) and FOXC2 (extracellular matrix remodeling and epithelial-mesenchymal transition) (Fig. 4C).

Similarly, using the derived SECAT attributes for GSEA with top-level Reactome pathways indicates global protein network dysregulation on several levels, affecting cell-cell communication, extracellular matrix organization, vesicle-mediated transport, immune system, and signal transduction configurations (Fig. 4A). The STRING-based PPI network of complex abundance and interactor ratio features (Fig. 4D) reveals that many detected differential proteins belong to cytosolic and mitochondrial ribosomal subunits as well as to proteins involved in Complex I and respiratory electron transport. These represent large heteromeric macromolecular assemblies spanning a wide abundance range; however, most are not substantially different in complex abundance or connectivity between the HeLa CCL2 and Kyoto. Some changes affect well-defined protein complexes as a whole, e.g., multiple subunits of the EKC/Keops, Mediator or Arp2/3 Complexes are affected in both, abundance and/or interactor ratio. A focused view of proteins that changed specifically at the complex abundance or interactor ratio levels (Fig. 4D) highlights two additional coordinated assemblies: the cadherin-catenin adherens junction complex (CDH2, CTNNB1, CTNNA1, and JUP) shows uniformly higher complex abundance in HeLa CCL2, complementing the elevated msVIPER-inferred WNT5A activity and reflecting the more mesenchymal character of the invasive subline; in contrast, five subunits of the biogenesis of lysosome-related organelles complex 1 (BLOC-1; BLOC1S1, BLOC1S3, BLOC1S4, BLOC1S5, and DTNBP1) show consistently higher complex abundance and interactor ratios in HeLa Kyoto, linking the vesicle-mediated transport GSEA signal to differential endosomal sorting capacity between the two sub-lines. Conversely, certain proteins with detected differences in complex abundance or interactor ratio are part of larger networks, e.g., cell surface, extracellular space or cell-cell junctions, which illustrates the benefits of representing proteins, PPIs and protein complexes as part of protein networks.

**Figure EV3:**
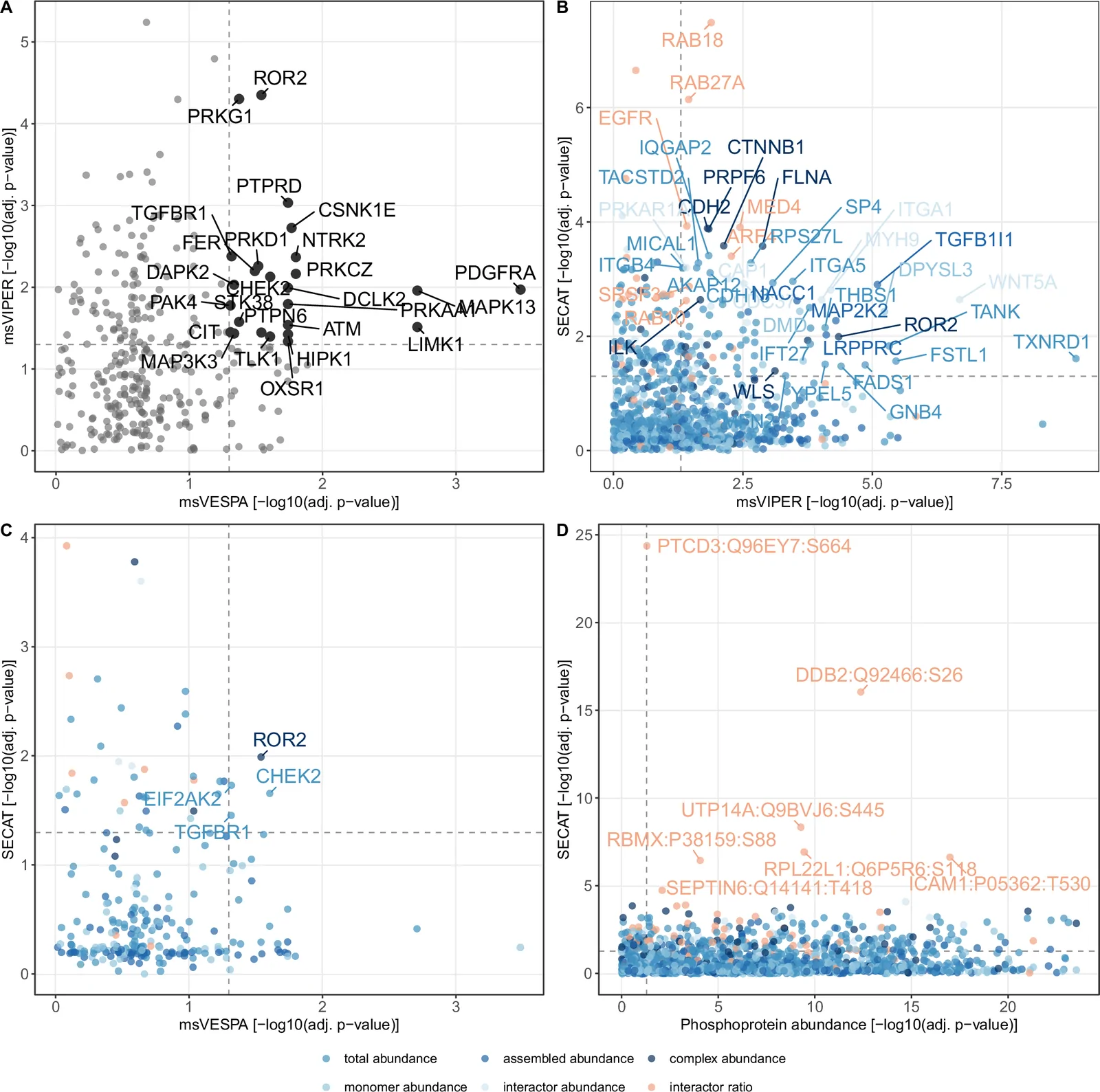
Cross-omics complementarity of differential protein activities between HeLa Kyoto and CCL2 cell lines. Scatter plots depict the pairwise comparison of statistical significance levels, represented as negative log10-transformed Benjamini–Hochberg (BH) adjusted *p* values (−log_10_(BH-adjusted *p* value)) (**A**) Pairwise correlation of msVESPA-inferred signaling activity (${n}_{\mathrm{kinases}}=549$, analytically estimated via a three-tailed enrichment test with nominal p-values BH-adjusted across all kinases) and msVIPER-inferred transcription factor activity (${n}_{\mathrm{TF}/\mathrm{co}-\mathrm{TF}}=\mathrm{6,156}$, similarly estimated via an analytical three-tailed enrichment test with BH adjustment). Doubly significant proteins (BH-adj. *p* value <0.05 in both) are labeled by gene symbol. (**B**) Correlation of msVIPER transcription factor activity and SECAT protein complex dynamics (estimated via SECAT’s semi-supervised peptide-level state classifier and adjusted using the BH method; best-performing state per protein is selected). Points are colored by SECAT attribution level. (**C**) Correlation of msVESPA signaling activity and SECAT protein complex dynamics. (**D**) Correlation of differential phosphopeptide abundance (calculated at the peptide level comparing HeLa Kyoto vs. CCL2 cell lines using a linear-modeling framework with the R package limma, with nominal *p* values BH-adjusted and the minimum adjusted *p* value selected per gene) and SECAT protein complex dynamics. Each point represents one protein; Spearman’s *ρ* is shown.

Comparing the sets of differentially active proteins identified by the three algorithms illustrates the heterogeneity of protein function and the complementary nature of the different omics layers and, therefore, the complexity of the molecular adaptation of the cells to the genetic drift (Fig. EV3). Minimal overlap was found between signaling (VESPA) and structural (SECAT) differentially active proteins (four proteins: CHEK2, ROR2, EIF2AK2, and TGFBR1), corresponding to 0.27% of the union, 5.48% of VESPA, and 0.28% of SECAT. In contrast, 191 out of 1414 differentially active SECAT proteins (13.51% of SECAT, 5.91% of the union, 9.51% of VIPER) were also identified as differentially active by VIPER. The intersection of differentially active proteins from VESPA and VIPER comprises 25 kinases and phosphatases (1.22% of the union, 34.25% of VESPA, and 1.24% of VIPER) with dual roles in signaling and gene regulation, such as PDGFRA, LIMK1 and MAPK13 (Fig. EV3A). The intersection of differentially active proteins between VIPER and SECAT (191 proteins) identifies proteins whose transcriptional regulation and complex dynamics are jointly altered, directly connecting functional layers. It includes N-cadherin (CDH2) and *β*-catenin (CTNNB1), linking the msVIPER-inferred WNT5A regulatory signal to the adherens junction complex abundance changes; Filamin-A (FLNA) and Myosin-9 (MYH9), connecting cytoskeletal gene regulatory activity to actin network complex remodeling; and NF*κ*B2 (NFKB2), whose transcriptional dysregulation coincides with differential SCF-mediated complex stoichiometry (Fig. EV3B). This confirms the complementarity and cross-layer convergence at the cellular state level of these analyses.

Out of the 549 kinases and phosphatases covered by VESPA, 313 were detected in the SECAT dataset. The majority of these (71.2%) were not differentially active between HeLa Kyoto and CCL2 by VESPA or SECAT analysis (Fig. EV3C). Since most proteins covered by SECAT are not kinases or phosphatases, we further assessed the relation between differential phosphosite abundance and protein complex dynamics (Fig. EV3D). Phosphorylation is frequently a core determinant of protein structural state and thus can also affect protein complex formation. Several proteins with differential protein complex dynamics, such as the DNA damage-binding protein 2 (DDB2), part of the UV-damaged DNA binding protein complex (UV-DDB), have been found to be differentially phosphorylated in the HeLa cells tested. In the case of DDB2, phosphosite S24 has also previously been found to be regulated by PRP4, and it has been suggested to be essential for cancer cell growth (Gao et al, 2013).

In summary, although SECAT, msVIPER and msVESPA provide functional protein attributes based on different molecular profiles with only partially overlapping proteome coverage, in general, the same perturbed biological processes are identified, suggesting that their complementarity could support integrative analysis based on the inferred PPI and gene regulatory networks.

### Network diffusion elucidates phenotype-associated molecular mechanisms

**Figure 5:**
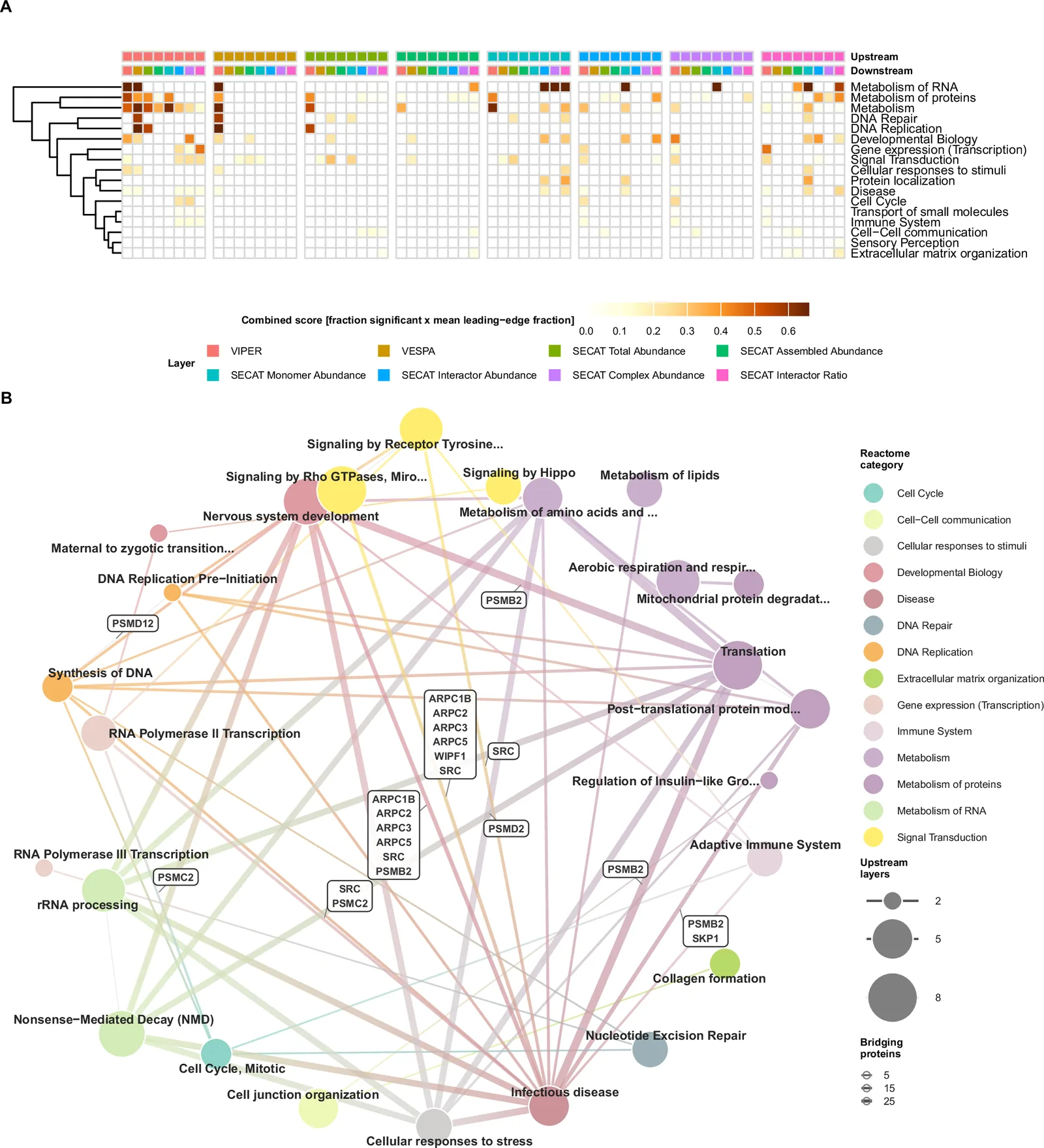
Network diffusion analysis of cross-layer pathway activation in HeLa CCL2 vs. Kyoto. (**A**) GSEA activation heatmap summarizing Reactome pathway activity across all 64 upstream ×  downstream layer combinations. Pathway enrichment for each layer combination was performed using the R package fgsea, employing a gene-permutation-based enrichment test (10,000 permutations) against Reactome L1 pathways, with nominal *p* values adjusted via the Benjamini–Hochberg (BH) method. Rows indicate Reactome top-level (L0) categories (hierarchically clustered); columns are grouped by upstream omics layer (eight blocks). Cell color represents a combined score, defined as the product of the fraction of significant L1 subpathways (BH-adj. *p* value <0.05) and the mean leading-edge fraction across those significant pathways, capped at the 95th percentile. Column annotation (top bar) indicates the upstream omics layer. Appendix Figs. S1–S8 and Dataset EV5 provide per-upstream-layer NES heatmaps and underlying data. (**B**) Reactome L1 pathway community network derived from cross-layer leading-edge overlap. Nodes represent Reactome L1 pathways with significant GSEA signal in at least one upstream ×  downstream combination (n=25); node color indicates the Reactome L0 top-level category; node size is proportional to the number of upstream omics layers activating the pathway. Edges connect pathway pairs whose leading-edge proteins overlap across different upstream layers (Jaccard index >0.05; n=57 edges); edge color interpolates between the two endpoint node category colors; edge width is proportional to the number of bridging proteins; gene symbols of up to four specificity-ranked bridging proteins are annotated on the highest-weight edges. Circular arc layout with nodes arranged within arcs by L0 category. Source data are available online for this figure.

Genetic or gene regulatory variability often manifests in distinct phenotypes through the proteotype. Therefore, our goal was to assess whether the context-specific PPI networks and SECAT’s six protein-level metrics (total, assembled, monomer, complex, and interactor abundance, and interactor ratio) could be used to elucidate molecular mechanisms differentiating CCL2 and Kyoto HeLa cells. To identify the effect of upstream signaling or gene regulatory activity and protein complex state changes on downstream layers, and to identify differential network modules between the two subtypes, we used diffuStats (Picart-Armada et al, 2018), a network diffusion approach. As upstream and downstream inputs, we used all proteins that showed a statistically significant difference between CCL2 and Kyoto cells based on each data modality, yielding eight upstream layers (the six SECAT metrics, msVESPA signaling activity, and msVIPER gene regulatory activity), as connected by the context-specific gene regulatory and PPI networks generated above (Methods). Note that SECAT’s “total abundance” metric serves as the SEC-based protein abundance reference within this framework; conventional protein abundance was not included as a separate diffusion layer, as it was measured under different experimental conditions and cannot be directly mapped onto the same context-specific co-fractionation PPI network. Affected pathways between different layers were then identified by GSEA using the first two levels of the Reactome pathway taxonomy. For each Reactome top-level category and each of the 64 upstream × downstream layer combinations, a combined score was computed as the product of the fraction of significant Reactome L1 subpathways and their mean leading-edge fraction, summarizing both the breadth and depth of pathway activation (Fig. 5A; Appendix Figs. S1–S8; Dataset EV5, Methods).

The pathway community network in Fig. 5B provides a map connecting these summary-level results to individual molecular players. Edge labels identify the proteins through which different Reactome L1 pathways share cross-layer diffusion signal, and two major biological themes emerge. First, a cytoskeletal remodeling and invasion cluster links the categories “Signaling by Rho GTPases”, “Infectious disease and nervous system development” through Arp2/3 subunits (ARPC1B/2/3/5) and WIPF1, proteins central to invadopodium formation and bacterial internalization. Second, a protein turnover and immune adaptation cluster connects the categories “Post-translational protein modification”, “Disease and immune system” through proteasome subunits (PSMB2, PSMC2, and PSMD2/12) and ubiquitin-pathway components. We used these two themes as a systematic guide to select the following examples discussing interactions between different data modalities (Fig. 5A,B; Appendix Figs. S1–S8).

#### Effects of kinase activity on protein complexes

**Figure 6:**
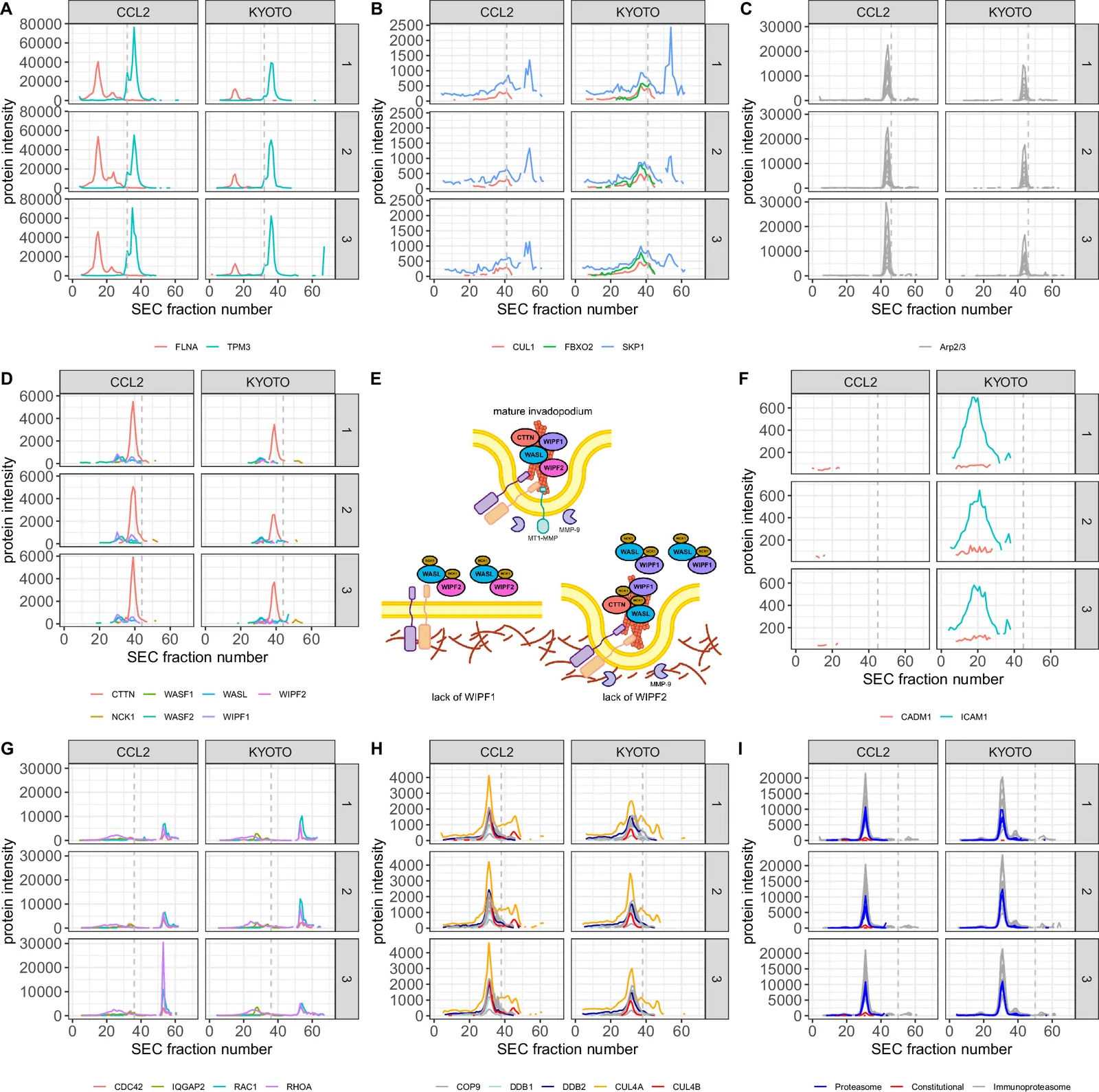
Protein-level SEC-SWATH profiles of HeLa CCL2 and Kyoto cell lines. Lines indicate different protein subunits, whereas the dashed line indicates the highest monomer threshold per group. All profiles show the mean of the three most intense peptide precursors per protein across n=3 biological replicates per cell line; shaded ribbons indicate ±1 standard deviation. (**A**) Protein complex abundance changes involving Filamin-A (FLNA) and the Tropomyosin alpha-3 chain (TPM3) between the two cell lines. (**B**) Differential configuration of the SKP1-CUL1-F-box (SCF) complex with the FBXO2 substrate recognition subunit. (**C**) Differential abundance of the actin-related protein complex 2/3 subunits. (**D**) Wiskott–Aldrich Syndrome family proteins and their involvement in different subcomplexes. (**E**) The molecular mechanism underlying WASP-dependent ECM degradation. Adapted from (García et al, 2016). (**F**) Differential abundance of ECM proteins. (**G**) Rho-GTPase activity mediated by IQGAP2 scaffolding. (**H**) Differential complex stoichiometry of COP9 Signalosome-CUL4-DDB1/2 supercomplexes. (**I**) Constitutive and Immunoproteasome abundance differences. Profiles are shown for qualitative visual comparison; no statistical test is applied to the SEC traces themselves. Source data are available online for this figure.

Among the most strongly differentially active signaling proteins, as assessed by VESPA, the analysis nominated the LIM domain kinase 1 (LIMK1), showing significantly lower activity in Kyoto compared to CCL2 cells (Fig. 4). LIMK1 was found to be a critical regulator of cytoskeleton dynamics, controlling matrix protein degradation and invadopodia (Scott et al, 2010). As part of the “Signaling by Rho GTPases, Miro GTPases and RHOBTB3” pathway (Fig. 5A; Appendix Fig. S2), network diffusion analysis identified Filamin-A (FLNA) and Tropomyosin alpha-3 chain (TPM3) to be affected at the level of protein complex abundance (Fig. 6A), in line with potentially reconfigured cytoskeleton structures and the previously observed lower *Salmonella* Typhimurium infection susceptibility of HeLa Kyoto (Liu et al, 2019).

#### Effects of transcription factor activity on protein complexes

**Figure EV4:**
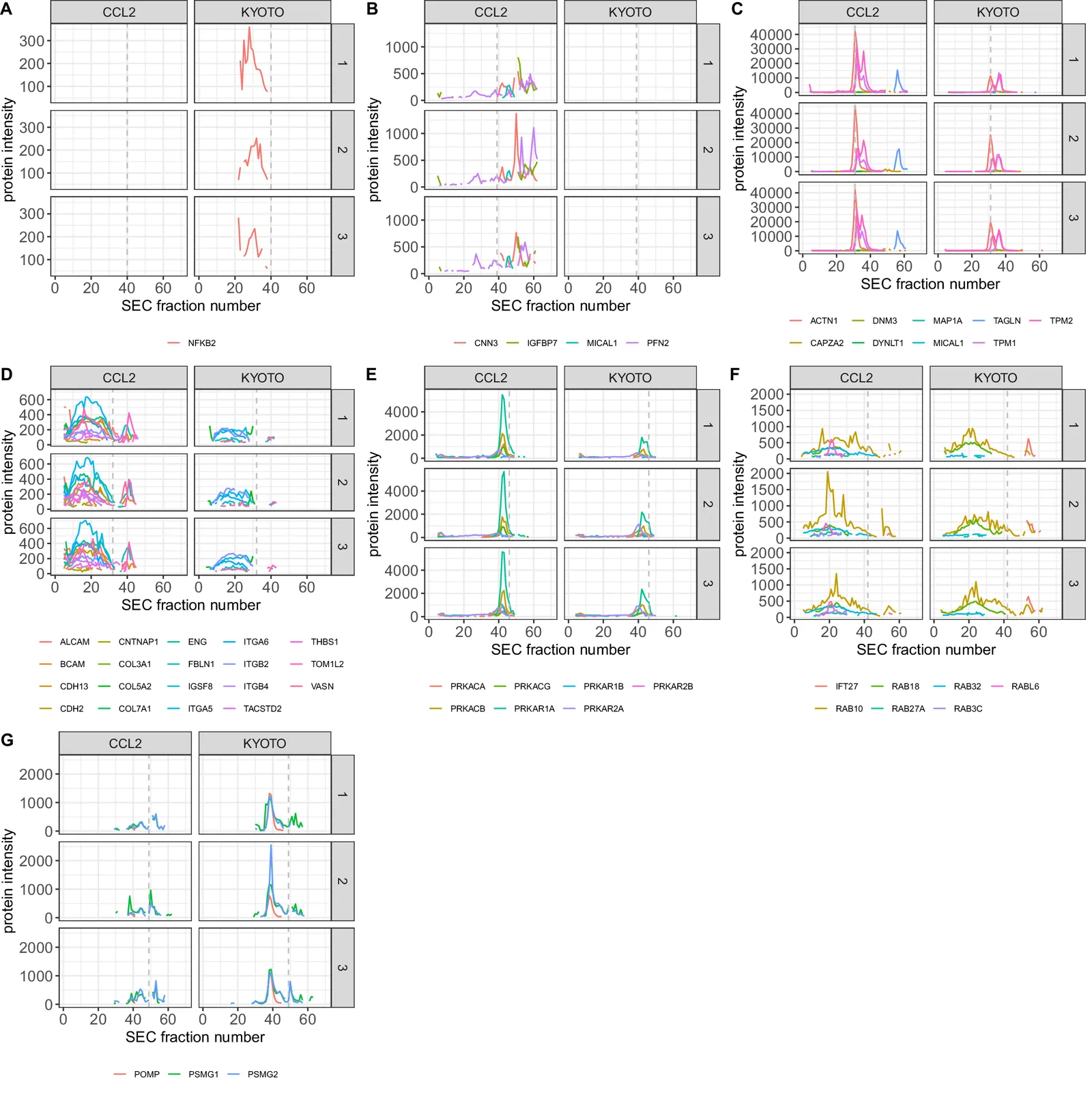
Supplemental protein-level SEC-SWATH profiles of HeLa CCL2 and Kyoto cell lines. Lines indicate different protein subunits, whereas the dashed line indicates the highest monomer threshold per group. All profiles show the mean of the three most intense peptide precursors per protein across n=3 biological replicates per cell line; shaded ribbons indicate ±1 standard deviation. (**A**) NF*κ*B (NFKB2) protein-level SEC-SWATH profiles. (**B**,** C**) Cytoskeleton-associated protein-level SEC-SWATH profiles. (**D**) ECM- and cell surface-associated protein-level SEC-SWATH profiles. (**E**) Protein kinase A complex protein-level SEC-SWATH profiles. (**F**) Rab-GTPase protein-level SEC-SWATH profiles. (**G**) Proteasome maturation protein and Proteasome assembly chaperone SEC-SWATH profiles.

Connecting VIPER gene regulatory activity with its downstream regulated targets, nominated protein complexes involved in extracellular matrix organization and in other signal transduction pathways as especially affected (Fig. 5A; Appendix Fig. S1). For instance, the Ras-related protein Rab-31 (RAB31), Tumor necrosis factor-inducible gene 6 protein (TNFAIP6) and integrin alpha-V (ITGAV) have all significantly lower activity in Kyoto vs. CCL2 cells. Moreover, network diffusion analysis identified the S-phase kinase-associated protein 1 (SKP1), part of the SKP1-CUL1-F-box (SCF) ubiquitin ligase complex, to be implicated via the innate immune system and associated processes (Cardozo and Pagano, 2004). The F-box only protein 2 (FBXO2), one of several substrate recognition components of this complex, was only detected as differentially active in Kyoto cells, suggesting reconfigured mediation of ubiquitin-based degradation (Cardozo and Pagano, 2004) (Fig. 6B). Interestingly, the master regulator NF*κ*B (NFKB2) was only detectable as differentially active in Kyoto cells. This represents an established recognition substrate for a different F-box protein, the F-box/WD repeat-containing protein 1A (BTRC) (Cardozo and Pagano, 2004), which is not covered by our dataset, suggesting that SCF complex reconfiguration may affect NF*κ*B protein abundance (Fig. EV4A).

#### Effects of monomeric proteins on protein complexes

Changes in upstream monomeric proteins mainly propagate to downstream protein complexes, with comparatively limited effects on other individual components in our dataset (Fig. 5A; Appendix Fig. S5). This may reflect the fact that many of the monomers showing the strongest differential activity between the HeLa lines are signaling or regulatory proteins, which can exert broad downstream effects. For example, the Insulin-like growth factor-binding protein 7 (IGFBP7) (Oh et al, 1996) stimulates cell adhesion and is only detectable in CCL2 cells (Fig. EV4B). Likewise, Profilin-2 (PFN2), Calponin-3 (CNN3), and F-actin-monooxygenase (MICAL1) are involved in actin binding and cytoskeletal organization, providing plausible links between changes in individual proteins and alterations in higher-order macromolecular complexes (Fig. EV4B).

Dysregulated cell-cell communication mechanisms represent an important component of carcinogenesis and metastasis, affecting the local tumor microenvironment and cell regulatory processes (Brücher and Jamall, 2014; Poltavets et al, 2018). In our previous multi-omics study (Liu et al, 2019), we found relevant differences in CCL2 vs. Kyoto cells, affecting cell doubling time, texture contrast of actin structures, response to perturbation with the tumor suppressor miRNA Let7, and *Salmonella* Typhimurium infection susceptibility. These phenotypes are connected to proteotypic differences in HeLa cells, such as the previously reported differential abundance of Actin-related protein 2/3 (Arp2/3) complex subunits. Arp2/3 has an established role in bacterial internalization by host cells through initiation of membrane ruffles (Criss and Casanova, 2003; Kreibich et al, 2015; Misselwitz et al, 2011), also referred to as invadopodia. Consistently, SECAT identified the subunits of Arp2/3 to be differentially abundant between the two cell lines (Fig. 6C). The subunits of the heteromeric complex are exclusively present in complex-bound conformation, indicating synchronized co-regulation. Arp2/3 is more abundant in HeLa CCL2, and similarly, other actin or cytoskeleton-related protein levels are more abundant or only detectable in this cell line (Fig. EV4C).

#### Protein complex dynamics

The Arp2/3 complex itself is activated by the Wiskott–Aldrich Syndrome family proteins that include WASP, N-WASP (WASL) and WAVE1/2 (WASF1/2) (Spiering and Hodgson, 2011). While the abundance levels and interactions between WASL and WASF1/2 are not substantially different between the two cell lines, the WASP/N-WASP interacting proteins 1 and 2 (WIPF1/2) are different in abundance and interactions. WIPF1 is more abundant in HeLa CCL2, whereas WIPF2 is only detectable in HeLa Kyoto cell lines (Fig. 6d). The overexpression of WIPF1 has been linked to increased actin polymerization mediated by the WASL-WIPF1 complex (Moreau et al, 2000; Ramesh et al, 1997), which, together with cortactin (CTTN), leads to invadopodium initiation and assembly. WIPF2 is further required to give rise to a mature invadopodium that can degrade the extracellular matrix via active matrix metalloproteinases (García et al, 2016). However, the ratio between WIPF1 and WIPF2 is critical for invadopodium initiation: higher levels of WIPF2 and lower levels of WIPF1 are ascribed to prohibit this step (García et al, 2016) (Fig. 6e). The high ratio of WIPF2 vs. WIPF1 in HeLa Kyoto thus suggests an immature status of the invadopodia which is further supported by the extracellular-matrix protein measurements, including the intercellular adhesion molecule 1 (ICAM1) and the cell adhesion molecule 1 (CADM1), which are less abundant in HeLa CCL2 due to their breakdown by active matrix metalloproteinases (García et al, 2016) but remain intact in HeLa Kyoto (Fig. 6F). Cancer cells frequently have modified surface markers (e.g., antigens and cell adhesion proteins) parts of a crucial process to evade the immune system. They are also differentially configured in HeLa Kyoto vs. HeLa CCL2 cell lines (Fig. EV4D).

Upstream regulators of WASP family proteins are primarily part of the Rho-GTPase protein family. This includes RhoA, Rac1 and CDC42 (Spiering and Hodgson, 2011). While we found these proteins to not be differently abundant between the two cell lines, SECAT identified the Ras GTPase-activating-like protein IQGAP2 in complex with CDC42 to be differently abundant between cell lines (Fig. 6G). Although Rac1 is also a known interactor of IQGAP2, its low measured abundance in complex-bound conformation limits assessment of its PPI. IQGAP2 in complex with CDC42 is more abundant in the HeLa CCL2 cell line, which is in line with its proposed scaffolding function to establish CDC42 and Rac1 activation by inducing a conformational change which mediates GTP binding (Sila Ozdemir et al, 2018). Rho GTPases themselves are targeted by upstream kinases, including Src, PKC, and PKA (Spiering and Hodgson, 2011). In addition to higher monomeric subunit levels of Src and PKC isomers in HeLa CCL2, SECAT found several proteins part of the protein kinase A complex to be more abundant in HeLa CCL2 (Fig. EV4E).

The structural rearrangements of invadopodia formation require elaborate logistics to transport the products from the endoplasmic reticulum via membrane vesicles to their destination. This process is primarily orchestrated by a network of proteins belonging to the Rab-GTPase superfamily (Hutagalung and Novick, 2011). Among the 74 superfamily members, 44 are covered by our dataset, and of those, 17 are differentially abundant between the cell lines (Fig. EV4F).

It was recently found that *Salmonella* Typhimurium produces the cysteine protease GtgE, which exclusively targets the inactivated form of Rab32, rendering it permanently inactive and thus preventing cells from delivering antimicrobial factors to limit infection (Wachtel et al, 2018). Rab32 is more abundant and has higher protein gene regulatory activity in HeLa CCL2, consistent with a vesicular transport system that is more actively engaged in antimicrobial defense. However, this elevated intracellular defense capacity is evidently insufficient to compensate for the increased bacterial entry facilitated by the Arp2/3-driven invadopodia machinery, which determines the higher infection susceptibility of CCL2 cells.

An essential part of the cellular immune system is protein degradation. The COP9 signalosome (CSN) is a heteromeric protein complex primarily involved in post-translational protein regulation of Cullin-RING ubiquitin ligases (CRLs), however, recent studies also implicated involvement in cancer-associated mechanisms (Salmena and Hakem, 2013). We found CSN subunit levels to be similar between the two cell lines, but we found differential stoichiometries of associated complex-bound DNA damage-binding proteins 1/2 (DDB1/2) (Fig. 6H). In response to DNA damage, it was proposed that CSN forms complexes with CRLs, acting on chromatin to promote DNA repair and checkpoint control (Hannß and Dubiel, 2011). The observed differential DDB1/2 complex stoichiometries might originate from subcomplexes which are not separable by SEC. It was proposed that different supercomplexes binding DDB1/2 have specific roles and mechanisms in tumor checkpoint control, however the exact mechanisms are not yet understood (Hannß and Dubiel, 2011). A similar adaptation can be observed in the related ubiquitin-proteasome system: In HeLa Kyoto, only subunits of the heteromeric constitutive proteasome complex are observed above the limit of detection (Fig. 6I). In HeLa CCL2 however, the observed peak can be attributed to both constitutive and immunoproteasome, a variant of the proteasome with increased activity during inflammation, also supporting antigen presentation of different peptide fragments by MHC class I molecules (van Deventer and Neefjes, 2010). Interestingly, the proteasome assembly chaperones 1/2, and the proteasome maturation protein are more abundant in HeLa Kyoto, which might indicate a preferred association with the constitutive proteasome (Fig. EV4G).

**Figure 7:**
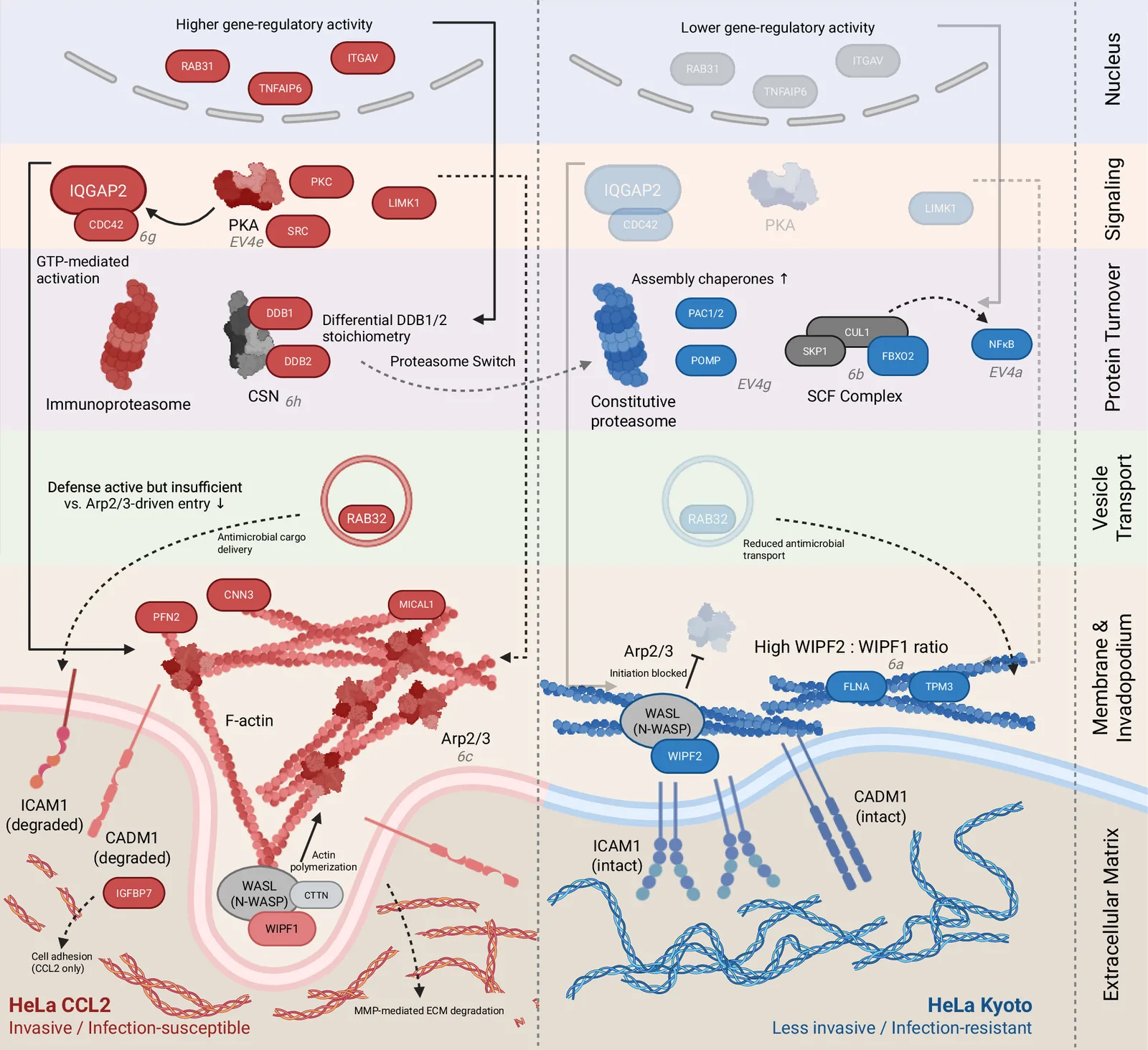
Molecular landscape of phenotypic divergence between HeLa CCL2 and HeLa Kyoto. This schematic summarizes the key molecular mechanisms driving the differential phenotypes (invasiveness/infection susceptibility) identified by our multi-omics analysis. The figure is organized in five zones shared between a HeLa CCL2 (Coral Red) half and a HeLa Kyoto (Teal Blue) half. (Bottom) High stoichiometry of the Arp2/3 complex and WIPF1 promotes the formation of invadopodia in CCL2, facilitating actin polymerization and ECM degradation (fragmented ICAM1/CADM1); in Kyoto, high levels of WIPF2 block invadopodium initiation, maintaining a stable cortical actin network (FLNA, TPM3) and intact ECM. (Lower middle) Rab32-positive transport vesicles deliver antimicrobial cargo more actively in CCL2, yet this elevated defense is insufficient against bacterial entry driven by invadopodia. (Middle) Distinct protein turnover machineries are observed: CCL2 utilizes the Immunoproteasome and shows differential COP9 signalosome–DDB1/2 stoichiometry, while Kyoto relies on the constitutive Proteasome and specific SCF complex configurations (FBXO2). (Upper middle) Upstream signaling via Rho GTPases (IQGAP2–CDC42) and kinases (LIMK1 and PKA) supports the invasive cytoskeletal state in CCL2. (Top) Gene regulatory networks reinforce the divergent cell states.

In summary, these findings support the differential and coordinated adaptation to genetic drift of the cellular immune system, cytoskeleton, cell surface and extracellular matrix in HeLa Kyoto vs. CCL2 starting from signaling pathways to vesicle-mediated transport of structural proteins and the ECM by covering the involved protein complexes (Fig. 7).

## Discussion

The measurement of comprehensive, multiomic (genome, transcriptome, proteome, and metabolome) molecular profiles across large cohorts has become accessible for many study types due to recent technological advances. Even though proteins are more closely associated with biological function than genes, transcripts or metabolites, their measured molecular abundance frequently does not directly correlate with the protein’s activity, thus impeding the association of protein abundance with individual phenotypes. This divergence of abundance and function is caused by several mechanisms acting on proteins, including post-translational modifications, three-dimensional structure, association with other biomolecules to form complexes and combinations thereof, that modulate the inherent activities and functions of proteins (Bludau and Aebersold, 2020). In essence, we hypothesize that these mechanisms enable proteins to adapt their functional states to changing cellular conditions, in turn affecting the cellular state. Here, we demonstrate that inference of functionally relevant protein attributes, specifically signaling or gene regulatory activity and protein complex state, can be more informative than mere protein abundance and provide direct mechanistic insights associated with phenotypes.

To test this hypothesis, we conducted a study that compared at a high level of detail two phenotypically diverse instances of a panel of HeLa cells that have diversified by genetic drift. For this purpose, we acquired protein correlation profiles of the cell lines in exponential growth, combined them with previously acquired transcriptomic, protein abundance, and phosphoproteomic profiles, and differentially quantified the protein network state by SECAT, msVESPA, and msVIPER at the level of PPI networks, signaling and gene regulation levels, respectively. We could then use this representation of molecular interactions by network diffusion analysis to assess the mechanistic effects of signaling activity, gene regulatory activity and protein complex states on each other. Further, quantitative assessment supported our previously raised (Liu et al, 2019) hypothesis of “protein complex buffering”, by demonstrating that proteins in assembled configurations are not as directly affected by CNV and mRNA abundance changes as their monomeric counterparts.

Our application to the HeLa Kyoto vs. CCL2 cell line comparison further illustrates the potential of protein correlation profiling for mechanistic investigations, requiring only three replicates per condition to identify many differentially connected modules. In addition to distinct, well-defined protein complexes such as the proteasome (Fig. 6I), differential protein complex engagement using non-Gaussian protein complex profiles (e.g., WASP-associated proteins, Fig. 6D) can be assessed to permit an unbiased overview of functional proteome changes between experimental conditions. This enabled us to extend our previous study on the differences of these two cell line types and identified adaptations regarding the cell surface and immune system, supporting previous findings on the involved molecular mechanisms.

While the present work employs HeLa cells as a model, their extensive characterization across genomic, transcriptomic, proteomic, and phenotypic layers (Liu et al, 2019) makes this system uniquely well-suited for the further interrogation of multifaceted protein attributes such as complex assembly states and regulatory network activities beyond protein expression levels. Importantly, our integrative framework demonstrates how such multifaceted attributes, derived from both measured complex adaptation and inferred activities, can more effectively connect molecular perturbations across molecular modes with emergent phenotypic outcomes. Several of the nominated mechanisms are directly testable: for instance, the predicted differential LIMK1 signaling activity could be confirmed by in vitro kinase assays with cofilin substrates, the inferred SCF complex reconfiguration involving FBXO2 by co-immunoprecipitation and ubiquitination assays, and the WIPF1/WIPF2 stoichiometry shift by live-cell imaging of invadopodium dynamics after targeted knockdown. Similarly, the predicted immunoproteasome switch (Fig. 6I) could be validated by activity-based profiling of proteasome catalytic subunits. These experiments would complement the computational predictions presented here and are facilitated by the well-characterized HeLa model system. Beyond HeLa, we envision our approach presented here as a general paradigm for studying proteins as adaptive agents whose diverse attributes jointly shape cellular states within complex biological systems.

Several limitations of the present study should be noted. Our analysis is based on a single, well-characterized cell line panel. While VIPER and VESPA have been successfully applied to primary tissues or patient-derived samples, the extension of SEC-SWATH-MS and SECAT to such systems, where material availability and sample complexity pose additional challenges, remains to be demonstrated. More generally, the SEC-SWATH-MS approach still requires substantial material input and instrument time, which currently limits throughput and precludes single-cell resolution. Recent technical advances, including the use of blue native electrophoresis instead of SEC chromatography and the availability of more sensitive and faster scanning mass spectrometers, have significantly reduced both, the amount of sample and time required to acquire the data (Uliana et al, 2023; Frommelt et al, 2024; Blasco Tavares Pereira Lopes et al, 2026; Doron-Mandel et al, 2025). Although our framework mitigates dependence on generic prior knowledge by constructing context-specific networks from the measured data itself, the underlying reference databases (STRING, Reactome, and kinase-substrate annotations) remain biased toward well-studied processes and may underrepresent novel or poorly characterized mechanisms. Furthermore, the “natural perturbation” model captures variation generated by genetic drift, which may not recapitulate directed perturbations (e.g., drug treatments or CRISPR knockouts) encountered in many experimental or clinical settings. Future work incorporating targeted CRISPR or pharmaceutical perturbations alongside multi-omics profiling would enable causal validation of the nominated mechanisms and extend the framework beyond observational comparisons.

In conclusion, the study demonstrates that integrating protein complex dynamics, signaling activity, and gene regulatory activity within their respective network contexts provides mechanistic insights into the molecular processes associated with or causative of phenotypes that go beyond what is accessible from protein, or for that matter, transcript abundance data alone. This framework further shows that network diffusion across these multiple molecular layers enables the systematic identification of cross-layer pathway activation and specific functional drivers. Collectively, this mechanistic approach complements both abundance-centric analyses and latent factor models (e.g., MOFA (Argelaguet et al, 2018)) by constructing context-specific molecular interaction networks from the measured data and mapping diverse molecular profiles onto them, thereby identifying specific functional states rather than abstract variance factors. Although the individual network contexts operate differently with only partial overlap, their joint interpretation at the level of individual protein subunits makes data integration and cross-layer inference tractable and can provide complementary and independent insights with relevance for the understanding of molecular mechanisms. We expect that the application and further development of the presented approach will substantially contribute to the development of artificial intelligence virtual cell (AIVC) models capable of providing adaptive, mechanism-based predictions of cell state.

## Methods

**Table:** Reagents and tools table

Reagent/resource	Reference or source	Identifier or catalog number
**Experimental models**		
HeLa CCL2 (HeLa 14)	Liu et al, 2019	—
HeLa Kyoto (HeLa 8)	Liu et al, 2019	—
**Chemicals, enzymes and other reagents**		
HEPES (50 mM, pH 7.5)	—	—
NaCl (150 mM)	—	—
NP-40 (0.5%)	—	—
TCEP (5 mM)	—	—
Urea (8 M)	—	—
Ammonium bicarbonate (AMBIC, 20 mM)	—	—
Iodoacetamide (IAA, 50 mM)	—	—
Formic acid (0.1%)	Fisher Scientific AG	—
Acetonitrile	Fisher Scientific AG	—
2-Propanol	—	—
Trypsin (MS grade, 1 μg/fraction)	Promega	—
Lysyl Endopeptidase (MS grade, 0.3 μg/fraction)	FUJIFILM Wako	—
iRT-Kit peptides	Biognosys AG	—
Pierce BCA Protein Assay Kit	Thermo Scientific	—
AcroPep Advance Filter Plates (Omega 10 K MWCO, 96-well)	Pall Corporation	—
SRT-C-SEC 500 column (300 × 21.2 mm, 500 Å, 5 μm)	Sepax-Tech	—
30 kDa MWCO membrane (buffer exchange)	—	—
Evotips (C18 stage tips)	Evosep Biosystems	—
C18 nanoConnect LC column (8 cm, 100 μm ID, 3 μm Reprocil)	PepSep	—
Nano Source Emitter Stainless Steel	Fisher Scientific AG	—
Column Performance Check Standard, Aqueous SEC 1	Phenomenex	AL0-3042
**Software**		
Spectronaut (rev. 12.0.20491.11.25470)	Biognosys AG	https://biognosys.com/software/spectronaut/
Analyst TF (1.7.1)	Sciex	—
Chronos	Axel Semrau GmbH	—
SECAT (1.0.6)	Rosenberger et al, (Rosenberger et al, 2020)	https://pypi.org/project/secat/
PyProphet (2.1.5)	Rosenberger et al. (Rosenberger et al, 2017)	https://pypi.org/project/pyprophet/
mapDIA	—	https://sourceforge.net/projects/mapdia/
VIPER/msVIPER (1.20; SECAT dep. / 1.44.0; msVIPER, msVESPA)	Alvarez et al. (Alvarez et al, 2016)	Bioconductor: viper
VESPA/msVESPA (1.0.2)	Rosenberger et al, (Rosenberger et al, 2024)	https://github.com/califano-lab/vespa
dVESPA/vespa.networks (1.0.0)	Rosenberger et al, (Rosenberger et al, 2024)	https://github.com/califano-lab/vespa
fgsea (1.36.2)	Sergushichev (Sergushichev, 2016)	Bioconductor: fgsea
diffuStats (1.30.0)	Picart-Armada et al, (Picart-Armada et al, 2018)	Bioconductor: diffuStats
limma (3.66.0)	—	Bioconductor: limma
org.Hs.eg.db (3.22.0)	—	Bioconductor: org.Hs.eg.db
ARACNe networks (1.36.0)	Lachmann et al, (Lachmann et al, 2016)	Bioconductor: aracne.networks
preprocessCore (1.72.0)	—	Bioconductor: preprocessCore
pheatmap (1.0.13)	—	CRAN: pheatmap
ggraph (2.2.2)	—	CRAN: ggraph
tidygraph (1.3.1)	—	CRAN: tidygraph
ggplot2 (4.0.2)	—	CRAN: ggplot2
R	R Foundation	https://www.r-project.org/
STRING (v11.0)	Szklarczyk et al, (Szklarczyk et al, 2019)	https://string-db.org/
CORUM (v3.0)	Giurgiu et al, (Giurgiu et al, 2019)	https://mips.helmholtz-muenchen.de/corum/
PrePPI (2016)	Garzón et al, (Garzón et al, 2016)	https://bhapp.c2b2.columbia.edu/PrePPI/
Reactome Pathway Database (v95)	Fabregat et al, (Fabregat et al, 2018)	https://reactome.org/
DepMap (Public 25Q3)	Tsherniak et al, (Tsherniak et al, 2017)	https://depmap.org/portal/
Human Protein Atlas	Thul et al, (Thul and Lindskog, 2018)	https://www.proteinatlas.org/
IID	Kotlyar et al, (Kotlyar et al, 2019)	https://iid.ophid.utoronto.ca/
BioPlex	Huttlin et al, (Huttlin et al, 2017)	https://bioplex.hms.harvard.edu/
UniProt Swiss-Prot (human, 2019-04-02)	UniProt Consortium	https://www.uniprot.org/
**Other**		
Evosep One LC system	Evosep Biosystems	—
SCIEX TripleTOF 6600	SCIEX	—
NanoSpray III ion source	SCIEX	—

### Protein complex dynamics of HeLa Kyoto vs. CCL2 cell lines

#### Sample preparation

HeLa CCL2 (HeLa 14) and Kyoto (HeLa 8) cell lines were obtained and cultured as described in our previous study (Liu et al, 2019). HeLa cells were tested for mycoplasma contamination using the GATC Biotech service (Germany) and confirmed to be negative.

#### SEC protein complex fractionation

Protein complex fractionation was performed as previously described (Heusel et al, 2019). HeLa CCL2 cells and HeLa Kyoto cells were thawed and lysed in mild conditions by homogenization with a lysis buffer composed of 0.5% NP-40 detergent and protease and phosphatase inhibitors (50 mM HEPES pH 7.5, 150 mM NaCl, 0.5% NP-40). Cell debris and membranes were removed by 15 min of ultracentrifugation (50,000×*g*, 4 °C), and the detergent was removed by a 30 kDa molecular weight cut-off membrane and exchanged with the SEC buffer (50 mM HEPES, pH 7.5, 150 mM NaCl). After 5 min of centrifugation at 16,900×*g* at 4 °C, the supernatant was directly injected into an SRT-C-SEC 500 column (dimensions 300 × 21.2 mm, pore size 500 Å, particle size 5 μm, Sepax-Tech, DE, USA). Per SEC run, 2.6 mg of native proteome extract (estimated by Pierce BCA Protein Assay Kit, Thermo Scientific) was injected and fractionated at a 2 ml/min flow rate on ice (4 °C), collecting 90 fractions at 0.4 min per fraction from 18 to 54 min post-injection. According to the protein elution window, fractions 1–70 (18– 46 min elution time) were collected for proteomic analysis. The calibration curve for SEC fractionation was obtained by measuring a protein standard mix (Column Performance Check Standard, Aqueous SEC 1, AL0-3042, Phenomenex, CA, USA) before each sample.

#### Sample preparation for mass spectrometry

Sample processing for bottom-up analysis of SEC fractions was performed on a 96-well plate MWCO filters (AcroPep Advance Filter Plates for Ultrafiltration 1 mL Omega 10 K MWCO; Pall Corporation, USA) (Potriquet et al, 2017). Prior to usage, the filters were washed twice with 200 μl of water that was successively removed by centrifugation at 1800×*g* for 30 min. Seventy fractions for each sample were loaded and concentrated on the filters through centrifugation, until complete removal of the SEC buffer. Protein denaturation and reduction was obtained after incubating the samples at 37 °C for 30 min with 5 mM of TCEP in 8 M Urea/20 mM AMBIC (pH 8.8). Alkylation of cysteine residues was performed by adding a final concentration of 50 mM IAA/20 mM AMBIC and incubating in the dark at room temperature for 1 h. After the reaction, the plates were centrifuged to remove the urea buffer and washed three times with 20 mM AMBIC. Protein digestion was carried out at 37 °C for 16 h, adding to each fraction 1 μg of trypsin (Promega, Switzerland) and 0.3 μg of Lysyl Endopeptidase (Mass Spectrometry grade, FUJIFILM Wako Pure Chemical Industries, Japan). The resulting peptides were collected by centrifugation and further washing of the filters with 20 mM AMBIC.

#### Mass spectrometry

LC-SWATH-MS analysis of the peptide fractions was performed on the Evosep One system (Evosep Biosystems, Denmark) (Bache et al, 2018) coupled to a SCIEX TripleTOF 6600 instrument (SCIEX, MA, USA) equipped with a NanoSpray III ion source (SCIEX). Due to the heterogeneity of the SEC protein fraction, we normalized the injections according to the fraction volume and to the amount of protein fractionated, corresponding to a total amount of 325 μg of proteins.

#### Sample loading

The samples were loaded in Evotips (Evosep Biosystems, Denmark), after resuspension in solvent A (0.1% FA water solution; Fisher Scientific AG, Switzerland) and the addition of iRT-Kit peptides (Biognosys AG, Switzerland). Prior to loading, the C18 stage tips (Evotips) were soaked with 100 μl of 2-propanol during the activation and the conditioning steps. The activation step consisted of washing with 20 μl of solvent B (0.1% FA in ACN, Fisher Scientific AG, Switzerland), followed by conditioning with 20 μl of solvent A. Prior to the sample loading step, 10 μl of solvent A was added on top of the tips, ensuring that the tips remained wet during the loading step. For all the steps, the tips were centrifuged for 1 min at a speed of 700×*g*. The last step (i.e., washing step) was performed using 100 μl of solvent A, and the loaded tips were added with 200 μl of solvent A for preserving the samples soaked during the entire injection of the batch.

#### Evosep-ESI-DIA-SWATH

The separation of peptides was performed selecting the “60 samples per day” method, consisting of 24 min of total cycle time, for 21 min of gradient length, 3 min of overhead time at a flow rate of 1 μl/min. A partial gradient was applied (0–35% solvent B) in order to elute the peptides from the Evotips by a couple of low-pressure pumps. The peptides were then pushed in a C18 nanoConnect LC column (8 cm column, ID 100-μm packed with 3-μm Reprocil, PepSep, Denmark) using a high-pressure pump and solvent A (Bache et al, 2018). The ESI coupling was obtained using a Nano Source Emitter Stainless Steel (Fisher Scientific AG). The ESI tuning parameters were the following: spray voltage, 2800 V; ion source gas flow (GS1), 16; curtain gas flow (CUR), 35; interface heater temperature (IHT), 100 °C; and declustering potential, 100. The Evosep system was controlled by the Axel Semrau Chronos software (Axel Semrau GmbH, Germany), while the mass spectrometer acquisition software was Analyst TF 1.7.1 (Sciex). Data-independent acquisition mass spectrometry was performed for the quantitative analysis of the 420 SEC fractions (70 fractions per sample) obtained from the 6 SEC runs (3 biological replicates × 2 cell lines). SWATH scans were performed using an updated scheme of 64 variably sized precursor co-isolation windows (Collins et al, 2017), covering similar precursor densities (in terms of number and intensity) within all SWATH windows. The SWATH windows covered the precursors ions in the range of 350–1500 m/z and 350–1500 in the MS2 SWATH scans, the accumulation time was 100 ms for the MS1 and 20 ms for each SWATH window, resulting in a cycle time of 1.38 s. For fragmentation, a rolling collisional energy with a collisional energy spread of 15 eV was applied.

#### Primary data analysis

The mass spectrometric data were analyzed with Spectronaut (Bruderer et al, 2015) (Biognosys AG, Schlieren, Switzerland; software revision 12.0.20491.11.25470) and the software-provided version of our previously published combined human spectral library (Rosenberger et al, 2014). The vendor-suggested parameters have been used, except for the normalization of peptide abundances, which has been deactivated. All mass spectrometry raw data, the exact used set of parameters and the processed primary data analysis results are available on the ProteomeXchange repository.

#### Data analysis

SECAT (Rosenberger et al, 2020) (version 1.0.6), PyProphet (Rosenberger et al, 2017; Teleman et al, 2015) (version 2.1.5) and VIPER (Alvarez et al, 2016) (version 1.20) were used for all data analyses with STRING (version 11.0 (Szklarczyk et al, 2019; Data ref: Szklarczyk et al, 2019)), CORUM (version 3.0 (Giurgiu et al, 2019; Data ref: Giurgiu et al, 2019)) and PrePPI (version 2016 (Garzón et al, 2016; Data ref: Garzón et al, 2016)) and default parameters if not otherwise specified. The raw profiles were normalized within SECAT as described previously (Rosenberger et al, 2020) (Appendix Figs. S9 and S10). Semi-supervised learning was conducted using CORUM as a positive network and CORUM-inverted as a negative network. All input data and parameters are provided on the Zenodo repository. CORUM-inverted was generated as described before (Rosenberger et al, 2020) by using the inverted set of PPI (i.e., all possible PPI that are not covered by CORUM) and removing all PPI in this set covered by STRING (Szklarczyk et al, 2019; Data ref: Szklarczyk et al, 2019), IID (Kotlyar et al, 2019; Data ref: Kotlyar et al, 2019), PrePPI or BioPlex (Huttlin et al, 2017; Huttlin et al, 2021 Data ref: Huttlin et al, 2021). Appendix Figs. S9 and S10 were generated as described previously (Rosenberger et al, 2020).

### Quantitative effects of copy number variation and mRNA abundance on protein complex assembly states

The multiomic data have been obtained from the original publication (Liu et al, 2019). The SECAT quantitative values have been filtered to ensure accurate measurements between replicates: The standard deviation was computed for the three replicates of both biological conditions separately and aggregated to the maximum of the two conditions. For each protein, the maximum of the two within-condition variances (across three replicates) was computed; only proteins with this value below the 75th percentile at the total level were retained for comparison.

Spearman’s *ρ* correlation coefficients are reported. Statistical tests between the correlation coefficients were conducted as proposed using Fisher’s transformation (Myers and Sirois, 2006).

#### Analysis of the essentiality of protein states

To assess the functional importance of buffering, we classified proteins into three groups based on their detection in SEC-SWATH-MS: “Paired” (detected in both monomer and assembled fractions), “Single monomer” (detected only in monomer fraction), and “Single assembled” (detected only in assembled fraction). We then mapped these proteins to CRISPR gene dependency scores from the DepMap portal (25Q3 release) (Tsherniak et al, 2017; Data ref: Tsherniak et al, 2017), specifically filtering for cervical cancer cell lines. Pairwise Wilcoxon rank-sum tests were used to compare the essentiality distributions between these groups.

### Multi-omic inference of differential protein activities and complex states

#### Differential phosphopeptide abundance analysis

Phosphoproteomic profiles were obtained from our previous study (Wu et al, 2021). Phosphopeptides were mapped to separate sites, and intensities were log2-transformed. The LIMMA R package (version 3.66.0) was used to conduct differential abundance analysis at the phosphopeptide level. Multiple-testing corrected *p* values were used to generate the correlation plots (Fig. EV3).

#### Inference of signaling activity

##### Preprocessing

Phosphoproteomic profiles were obtained from our previous study (Wu et al, 2021). Phosphopeptides were mapped to separate sites, and intensities were log2-transformed. Missing values were imputed row-wise as described previously (Rosenberger et al, 2024). All values were rank-normalized (Paull et al, 2021).

##### Signaling network generation

The dVESPA (Rosenberger et al, 2024) module (vespa version 1.0.0, vespa.networks version 1.0.0, vespa.db version 1.0.0) was used with default parameters for signaling network generation, as described previously. The preprocessed HeLa phosphoproteomic profiles were used to select the most representative signalons from a collection of 10 CPTAC cancer datasets (COAD (Vasaikar et al, 2019), OV (Hu et al, 2020; McDermott et al, 2020), BRCA (Krug et al, 2019), UCEC (Dou et al, 2020), CCRCC (Clark et al, 2019), LUAD (Gillette et al, 2020), PBT (Petralia et al, 2020), GBM (Wang et al, 2021), HCC (Gao et al, 2019), HNSCC (Huang et al, 2021)). HSM/P1-constrained substrate-level signalons were used for all the following steps.

##### Differential Signaling activity inference

msVESPA (Rosenberger et al, 2024) (vespa version 1.0.2, viper version: 1.44.0) was then used to compare HeLa Kyoto vs. HeLa CCL2 cell lines. msVESPA was configured with 1000 bootstraps (averaged by mean) and the parametric approach with the meta-signalons generated from above, adaptively restricted to the top 50 substrates. Pleiotropic cross-talk correction was enabled as described previously (Rosenberger et al, 2024). Results are reported with BH-adjusted *p* values; UniProt accessions are recovered directly from the FASTA gene-name annotations used during regulon construction, with supplementary Entrez and Ensembl identifiers mapped via Bioconductor/org.Hs.eg.db: 3.22.0.

#### Inference of protein gene regulatory activity

##### Preprocessing

Transcriptomic profiles were obtained from our previous study (Liu et al, 2019). Transcript RPKM values were averaged per Entrez identifier, log2-transformed, and rank-normalized as described previously (Paull et al, 2021).

##### Gene regulatory network generation

All TCGA-based ARACNe (Lachmann et al, 2016; Margolin et al, 2006) networks (Paull et al, 2021) were used to generate an optimal set of regulons by the metaVIPER approach, as described previously (Ding et al, 2018).

##### Differential TF activity inference

msVIPER (Alvarez et al, 2016) (viper version: 1.44.0) was then used with 1000 bootstraps (averaged by mean) and the parametric approach with the meta-regulon generated above to compare HeLa Kyoto vs. HeLa CCL2 cell lines. Results are reported with BH-adjusted *p* values and with Ensembl and UniProt identifiers, respectively, mapped using Bioconductor/org.Hs.eg.db: 3.22.0.

#### DepMap gene essentiality analysis

To test whether the highest-scoring candidates were enriched for cell-essential genes in cervical cancer, the top 100 proteins ranked by statistical significance (−log_10_ (BH-adj. *p* value)) were selected from each omics layer. CRISPR-based gene dependency scores from the DepMap Public 25Q3 release (Tsherniak et al, 2017; Data ref: Tsherniak et al, 2017) were restricted to cervical cancer cell lines (OncotreeLineage = “Cervix”; n=19 cell lines with CRISPR dependency data), and median dependency scores were computed per gene. For each method, the dependency score distribution of the top 100 nominated proteins was compared to the total protein abundance control using pairwise Wilcoxon rank-sum tests.

#### HPA subcellular localization analysis

Subcellular localization enrichment was assessed using fast gene set enrichment analysis (fgsea, v1.36.2) (Sergushichev, 2016). Gene sets were constructed from the Human Protein Atlas (HPA) (Thul and Lindskog, 2018; Data ref: Thul and Lindskog, 2018) subcellular location annotation, using the “Main location” field only. Activity scores were converted from UniProt accessions to Ensembl identifiers using the DepMap gene annotation table. For each omics layer, a ranked score vector was submitted to fgsea with 10,000 permutations, and enrichment results were filtered for adjusted *p* value <0.05 in at least one method.

#### Let7 perturbation correlation analysis

To validate the phenotypic relevance of the identified candidates, per-protein Spearman correlations were computed between activity scores and the *Salmonella* infection rate measured across 10 HeLa sub-cell lines from the Let7 miRNA perturbation experiment (Liu et al, 2019). Protein scores were derived from four omics layers: (i) total protein abundance from mapDIA-normalized DIA-MS data, (ii) SECAT assembled and monomer abundance log2 fold-changes, (iii) VESPA integrated kinase activity NES values, and (iv) VIPER TF activity NES values. For VESPA, gene symbols were mapped back to UniProt accessions using the DepMap gene annotation table; SECAT and VIPER outputs are indexed by UniProt accession directly. Only proteins present in both the activity score table and the phenotypic data were included. The distribution of per-protein correlation coefficients was compared across methods.

### Synergies between differential kinase/phosphatase, transcription factor activities, and protein complex dynamics

#### Gene set enrichment analysis

The gene set enrichment analysis in Fig. 4A was generated using fgsea (version 1.36.2) (Sergushichev, 2016) with 10,000 permutations (nPermSimple = 10,000). For SECAT, signed scores on all levels were computed as sign(log_2_ (fold-change)) × (−log_10_ (BH-adj. *p* value)); for msVESPA and msVIPER, NES scores were used directly. The Reactome Pathway Database (Fabregat et al, 2018; Data ref: Fabregat et al, 2018) (version 95; only top-levels) was used. Opacity in the figure indicates significance (BH-adjusted *p* value <0.05). Figure 4b,c depict the top ten master regulators (five most activated and five most repressed, ranked by NES) from msVESPA and msVIPER, respectively. For each regulator, bootstrap NES distributions (1000 bootstraps) are shown as boxplots (lower and upper hinges: first and third quartiles; center line: median; whiskers: 1.5 × IQR) using the ggplot2 (4.0.2) “geom_boxplot” defaults. Bars alongside each boxplot represent the leading-edge target signatures, colored by direction of regulation.

#### Protein–protein interaction network

To annotate and visualize differential proteins in Fig. 4D between the HeLa CCL2 and HeLa Kyoto cell lines identified by SECAT, we constructed the STRING-based PPI network using igraph and visualized it with ggraph (version 2.2.2) in R. CORUM (version 5.2) complex annotations were used to draw convex hull overlays around co-complex subunits.

### Network diffusion elucidates phenotype-associated molecular mechanisms

#### Network diffusion analysis

A unified, undirected protein interaction network was assembled from three complementary sources: SECAT protein–protein interaction edges (extracted from the STRING-prior run at 5% global *q* value cutoff), VESPA regulatory edges (linking kinases to their phosphorylation targets from the substrate regulon), and VIPER regulatory edges (TF-target edges from the ARACNe regulons, filtered to regulators with BH-adj. *p* value <0.01 in the msVIPER result and edges with a likelihood score >0.9). All three edge sets were merged, and multi-edges and self-loops were removed, providing an integrated network topology for the diffusion analysis.

For each of the eight molecular layers (VIPER TF activities, VESPA kinase activities, and the six SECAT abundance/stoichiometry metrics), a seed vector was constructed from proteins meeting a statistical significance threshold (BH-adj. *p* value <0.05). Seed values were set to their absolute activity or abundance score (|NES| or |log_2_ (fold-change)|). Proteins not present in the network were excluded, and duplicate UniProt accessions were resolved by retaining the entry with the highest absolute score.

Random-walk-based network diffusion was performed using the diffuStats package (Bioconductor/diffuStats: 1.30.0) (Picart-Armada et al, 2018) with the z-score method (diffuse(method = “z”)). All eight seed vectors (VIPER, VESPA, and the six SECAT abundance levels) were submitted simultaneously as a single heat matrix (nodes × layers), and diffusion z-scores were computed in one pass over the combined network. The z-score method normalizes propagated scores against the expected background diffusion, making scores comparable across layers. To capture cross-layer signal propagation, 64 pairwise upstream × downstream combinations of the eight layers were evaluated. For each pair, a combined score per protein was defined as the product of the two diffusion z-scores. Gene set enrichment analysis was then run for each of the 64 layer combinations against Reactome L1 pathways (second level of the Reactome taxonomy) using fgsea (version 1.36.2) (Sergushichev, 2016) with a minimum gene set size of 5.

The overview heatmap (Fig. 5A) summarizes pathway activation across all 64 combinations. Each cell shows a combined score for a Reactome L0 top-level category (rows, hierarchically clustered) and an upstream × downstream layer combination (columns, grouped by upstream layer). The combined score is the product of two quantities: the fraction of tested L1 subpathways that are significant (BH-adj. *p* value <0.05) and the mean leading-edge fraction (ratio of leading-edge proteins to pathway size) across those significant pathways. Values were capped at the 95th percentile. Appendix Figs. S1–S8 provides per-upstream-layer NES heatmaps at L1 resolution.

The pathway community network (Fig. 5B) visualizes cross-layer convergence. Nodes represent Reactome L1 pathways significant in at least one upstream × downstream combination (n=25); node size encodes the number of activating upstream layers and node color the L0 category. An edge connects two pathways whose leading-edge proteins, drawn from different upstream layers, share a Jaccard overlap >0.05 (n=57 edges); edge width is proportional to the number of bridging proteins, with up to four specificity-ranked gene symbols annotated on the highest-weight edges. A circular arc layout groups nodes by L0 category. All panels were generated using pheatmap (version 1.0.13), ggraph (version 2.2.2) and tidygraph (version 1.3.1).

Visualization of protein-level SEC-SWATH-MS profiles in Figs. 6 and EV4 was conducted using the R package “ggplot2” by averaging the three most intense peptide precursors per protein.

### Use of AI tools

GitHub Copilot (GitHub/Microsoft) was used to improve the style and readability of the manuscript text and to improve the quality, portability, and reproducibility of the analysis code. All AI-assisted outputs were reviewed, verified, and approved by the authors, who bear full responsibility for the content of this work.

## Supplementary information

Appendix

Peer Review File

Dataset EV1

Dataset EV2

Dataset EV3

Dataset EV4

Dataset EV5

Source data Fig. 2

Source data Fig. 3

Source data Fig. 4

Source data Fig. 5

Source data Fig. 6

Expanded View Figures

## Data Availability

The datasets and computer code produced in this study are available in the following databases: SEC-SWATH-MS raw and processed proteomics data: ProteomeXchange Consortium via the MASSive (Vizcaíno et al, 2013) partner repository (https://proteomecentral.proteomexchange.org/cgi/GetDataset?ID=PXD067680), dataset identifier PXD067680. Analysis code: GitHub (https://github.com/grosenberger/HeLa-MPX). Processed data and Dataset EV files: Zenodo (https://doi.org/10.5281/zenodo.16985687). The source data of this paper are collected in the following database record: biostudies:S-SCDT-10_1038-S44320-026-00228-3.
