# Supplementary material for: Complex assembly and activity states as multifaceted protein attributes explaining phenotypic variability
Source: Mol Syst Biol. 2026 Jul 22;22(10):1551–77. doi: 10.1038/s44320-026-00228-3 (PMC13639060; doi:10.1038/s44320-026-00228-3)
Supplement: Supplementary file 1 [file 44320_2026_228_MOESM13_ESM.pdf]

Expanded View Figures

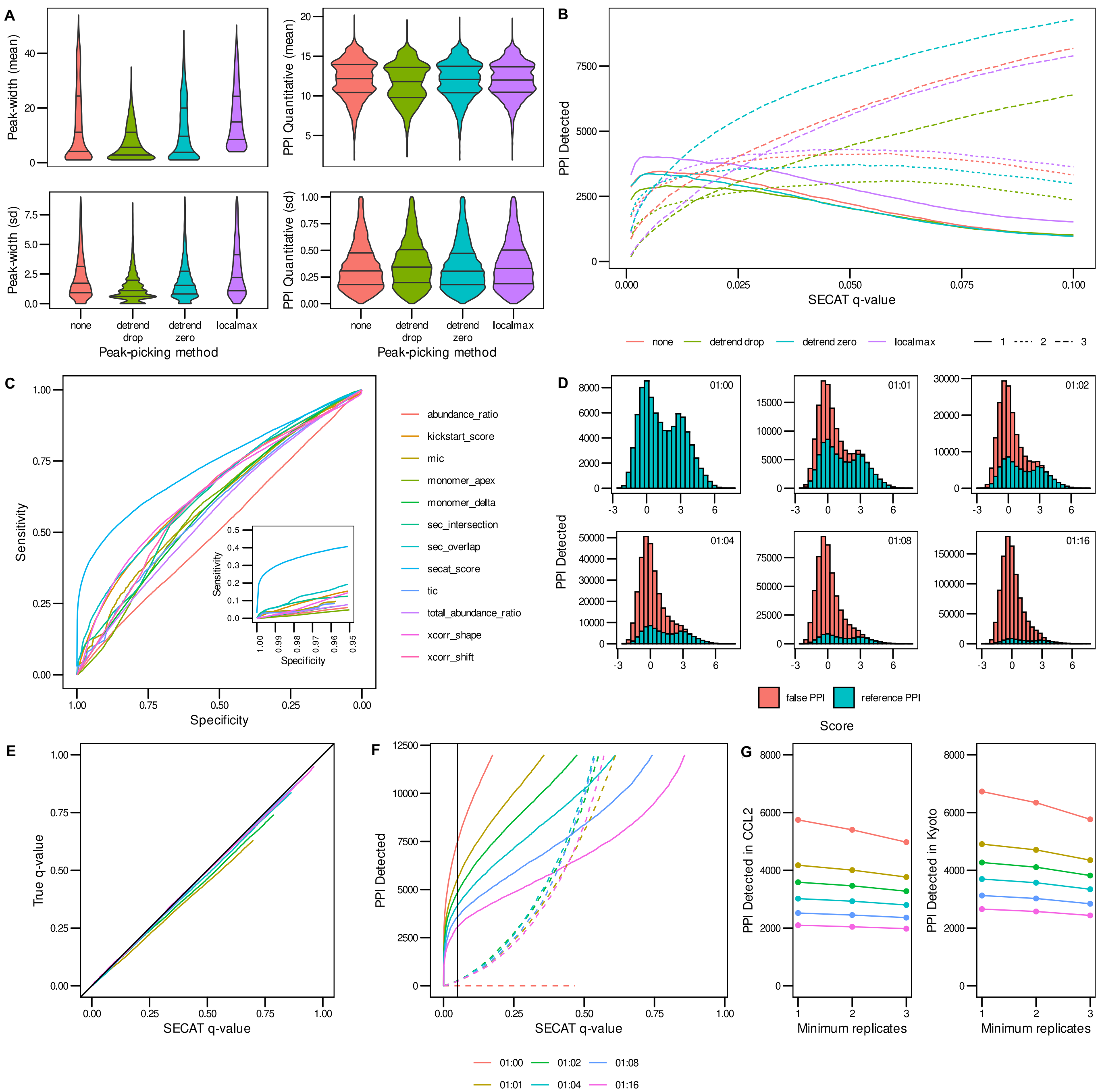

Figure EV1. SECAT signal processing and PPI detection performance.

(**A**) The effect of different peak-picking methods for signal processing on peak-width and PPI quantification within replicates of the same experimental condition is depicted in boxplots (center line, median; box limits, first and third quartiles; whiskers, 1.5× IQR). (**B**) Sensitivity of PPI detection vs. SECAT *q* value for peak-picking options. The PPI were filtered to a global context q-value < 0.05. Colors indicate different peak-picking options; line types indicate the number of detections with the three replicates per condition. (**C**) The receiver-operating characteristic (ROC) illustrates the sensitivity vs. specificity of the different SECAT subscores, the kickstart score and the integrated SECAT score. (**D**) The SECAT score histograms depict the dilution of the reference PPI network (true: CORUM) with false PPI (false: CORUM-inverted) for the benchmark (Methods). (**E**) The SECAT estimated q-value is accurate as evaluated against the ground truth for all levels of reference PPI network accuracy. (**F**) The sensitivity of PPI detection in dependency of SECAT *q* value for different levels of reference PPI network accuracy is depicted. (**G**) Consistency of PPI detection between replicates of the same conditions. All panels: $n = 3$ biological replicates per cell line (HeLa CCL2 and HeLa Kyoto).

 © The Author(s)

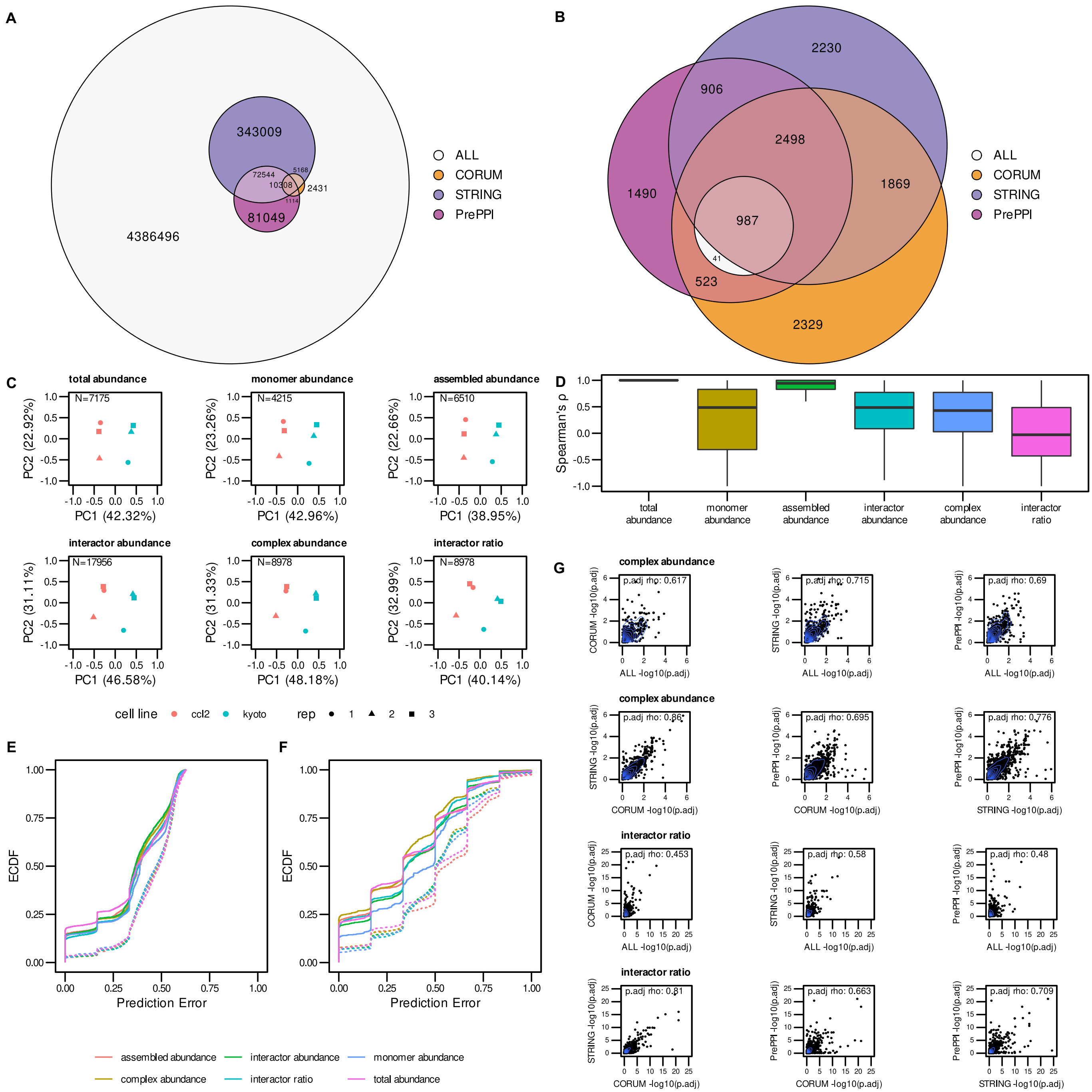

Figure EV2. SECAT PPI quantification and Network integration.

(**A**) The Euler diagram indicates the overlap of STRING, PrePPI and CORUM reference PPI networks, resulting in at least a partial overlap of the interactors in any of the replicates. (**B**) Overlap of confident (*q* value <0.05) PPI detections after SECAT analysis with different reference PPI networks. (**C**) Quantitative SECAT features can be used to separate experimental conditions and replicates. (**D**) Spearman's correlation of features (PPI: summarized by averaging) to total-level features is depicted in individual boxplots (center line, median; box limits, first and third quartiles; whiskers, 1.5× IQR). (**E**) Predictive error for feature-wise leave-one-out cross-validated logistic regression classification of replicates to biological conditions. (**F**) Predictive error for group-wise leave-one-out cross-validated logistic regression classification of replicates to biological conditions. Groups represent several features connected to a core feature based on the PPI network. (**G**) Correlation of node-level integrated BH-adj. p-values estimated using different reference PPI networks. Spearman's rank correlation coefficient (ρ) is depicted, with statistical significance evaluated using a two-sided Spearman's rank correlation test. All panels: $n = 3$ biological replicates per cell line (HeLa CCL2 and HeLa Kyoto).

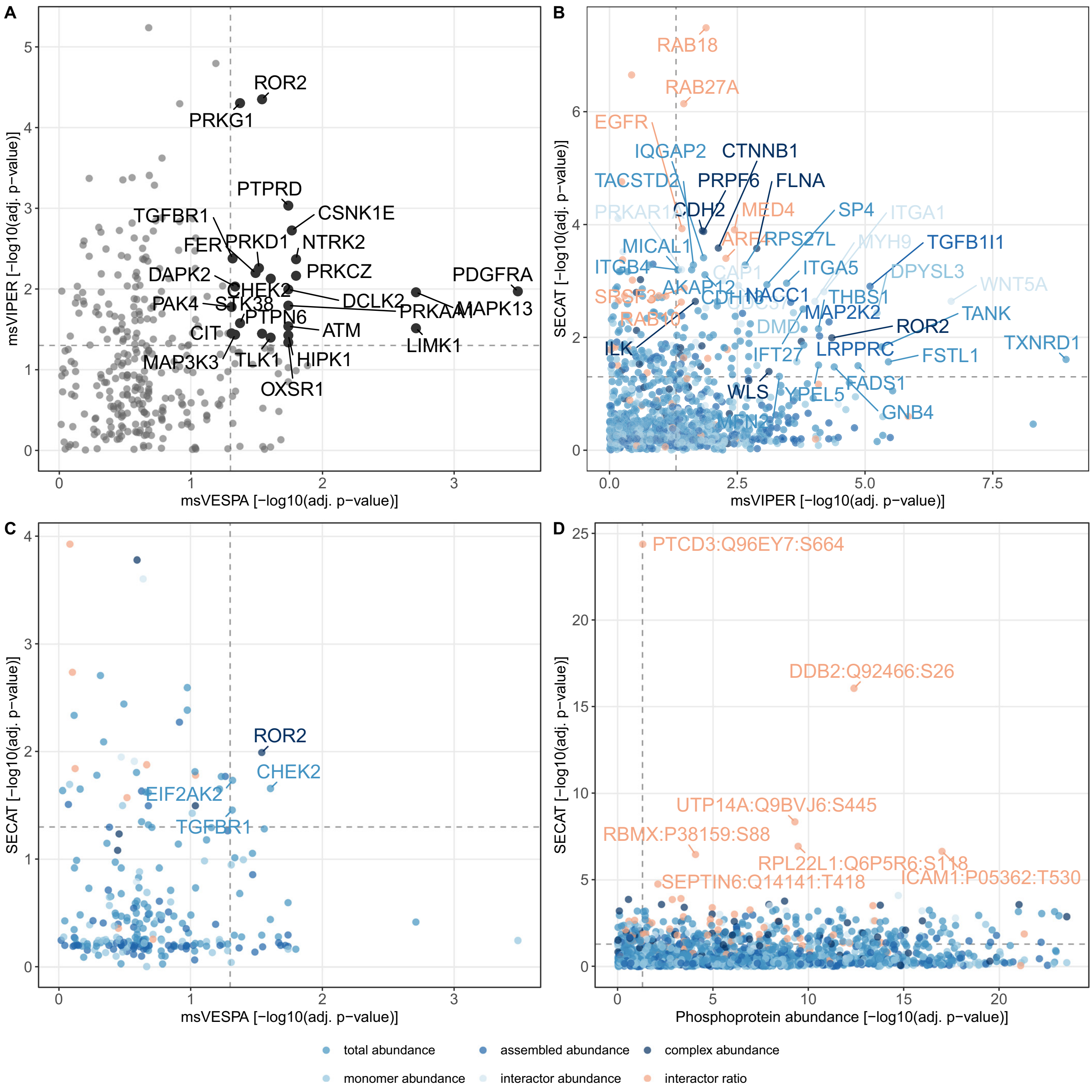

Figure EV3. Cross-omics complementarity of differential protein activities between HeLa Kyoto and CCL2 cell lines.

Scatter plots depict the pairwise comparison of statistical significance levels, represented as negative log10-transformed Benjamini–Hochberg (BH) adjusted *p* values ($-\log_{10}$(BH-adjusted *p* value)) (**A**) Pairwise correlation of msVESPA-inferred signaling activity ($n_{\text{kinases}} = 549$, analytically estimated via a three-tailed enrichment test with nominal p-values BH-adjusted across all kinases) and msVIPER-inferred transcription factor activity ($n_{\text{TF/co−TF}} = 6{,}156$, similarly estimated via an analytical three-tailed enrichment test with BH adjustment). Doubly significant proteins (BH-adj. *p* value <0.05 in both) are labeled by gene symbol. (**B**) Correlation of msVIPER transcription factor activity and SECAT protein complex dynamics (estimated via SECAT's semi-supervised peptide-level state classifier and adjusted using the BH method; best-performing state per protein is selected). Points are colored by SECAT attribution level. (**C**) Correlation of msVESPA signaling activity and SECAT protein complex dynamics. (**D**) Correlation of differential phosphopeptide abundance (calculated at the peptide level comparing HeLa Kyoto vs. CCL2 cell lines using a linear-modeling framework with the R package limma, with nominal *p* values BH-adjusted and the minimum adjusted *p* value selected per gene) and SECAT protein complex dynamics. Each point represents one protein; Spearman's ρ is shown.

 © The Author(s)

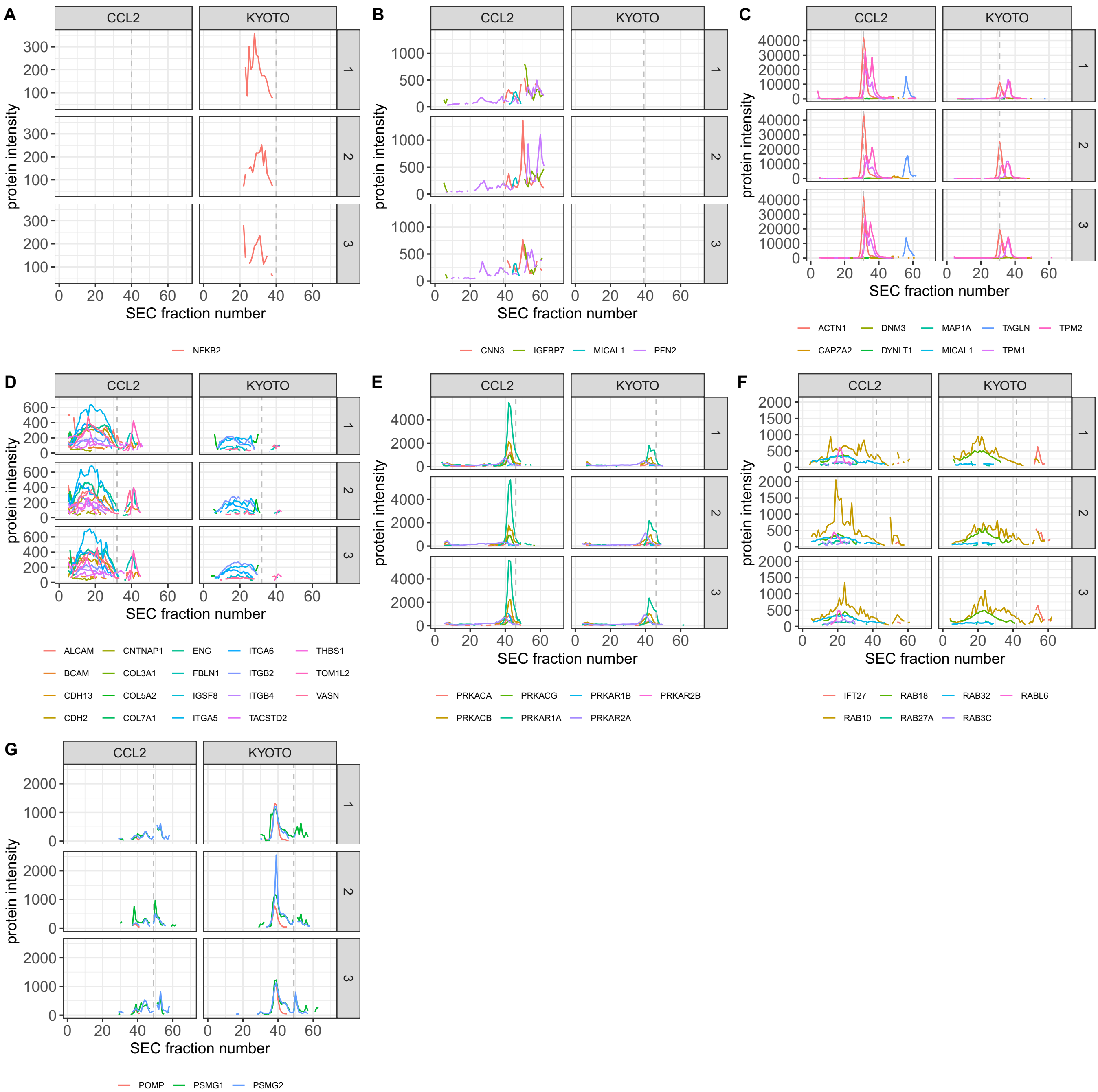

Figure EV4. Supplemental protein-level SEC-SWATH profiles of HeLa CCL2 and Kyoto cell lines.

Lines indicate different protein subunits, whereas the dashed line indicates the highest monomer threshold per group. All profiles show the mean of the three most intense peptide precursors per protein across $n = 3$ biological replicates per cell line; shaded ribbons indicate ±1 standard deviation. (**A**) NFκB (NFKB2) protein-level SEC-SWATH profiles. (**B**, **C**) Cytoskeleton-associated protein-level SEC-SWATH profiles. (**D**) ECM- and cell surface-associated protein-level SEC-SWATH profiles. (**E**) Protein kinase A complex protein-level SEC-SWATH profiles. (**F**) Rab-GTPase protein-level SEC-SWATH profiles. (**G**) Proteasome maturation protein and Proteasome assembly chaperone SEC-SWATH profiles.
