# Supplementary material for: Complex assembly and activity states as multifaceted protein attributes explaining phenotypic variability
Source: Mol Syst Biol. 2026 Jul 22;22(10):1551–77. doi: 10.1038/s44320-026-00228-3 (PMC13639060; doi:10.1038/s44320-026-00228-3)
Supplement: Supplementary file 2 [file 44320_2026_228_MOESM1_ESM.pdf]

Appendix

Complex Assembly and Activity States as Multifaceted Protein Attributes Explaining Phenotypic Variability

Table of Contents

- Appendix Figure S1. Downstream pathway NES heatmap – upstream VIPER layer.....2
- Appendix Figure S2. Downstream pathway NES heatmap – upstream VESPA layer.....3
- Appendix Figure S3. Downstream pathway NES heatmap – upstream SE-CAT total abundance layer.....4
- Appendix Figure S4. Downstream pathway NES heatmap – upstream SE-CAT assembled abundance layer.....5
- Appendix Figure S5. Downstream pathway NES heatmap – upstream SE-CAT monomer abundance layer.....6
- Appendix Figure S6. Downstream pathway NES heatmap – upstream SE-CAT interactor abundance layer.....7
- Appendix Figure S7. Downstream pathway NES heatmap – upstream SE-CAT complex abundance layer.....8
- Appendix Figure S8. Downstream pathway NES heatmap – upstream SE-CAT interactor ratio layer.....9
- Appendix Figure S9. Raw peptide precursor intensities over the SEC profile.....10
- Appendix Figure S10. Normalized peptide precursor intensities over the SEC profile.....11

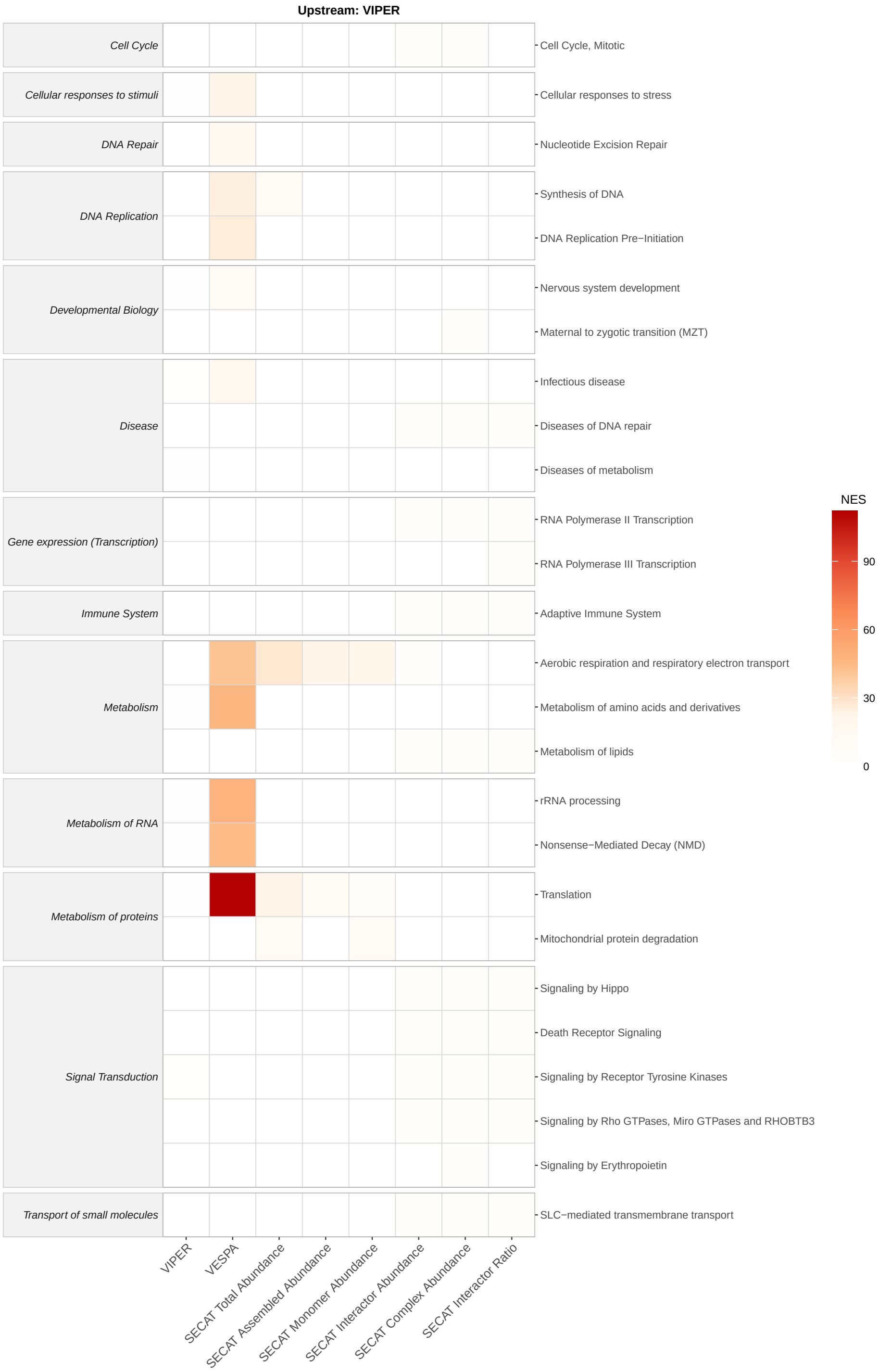

Appendix Figure S1. Downstream pathway NES heatmap for network diffusion with upstream VIPER (transcription factor activity) layer. Rows: all Reactome L1 pathways significant (BH-adj. p-value < 0.05) in at least one upstream $\times$ downstream combination for this upstream layer, grouped by Reactome L0 top-level category. Columns: the eight downstream omics layers. Cell colour: normalised enrichment score (NES), capped at the per-upstream-layer maximum.

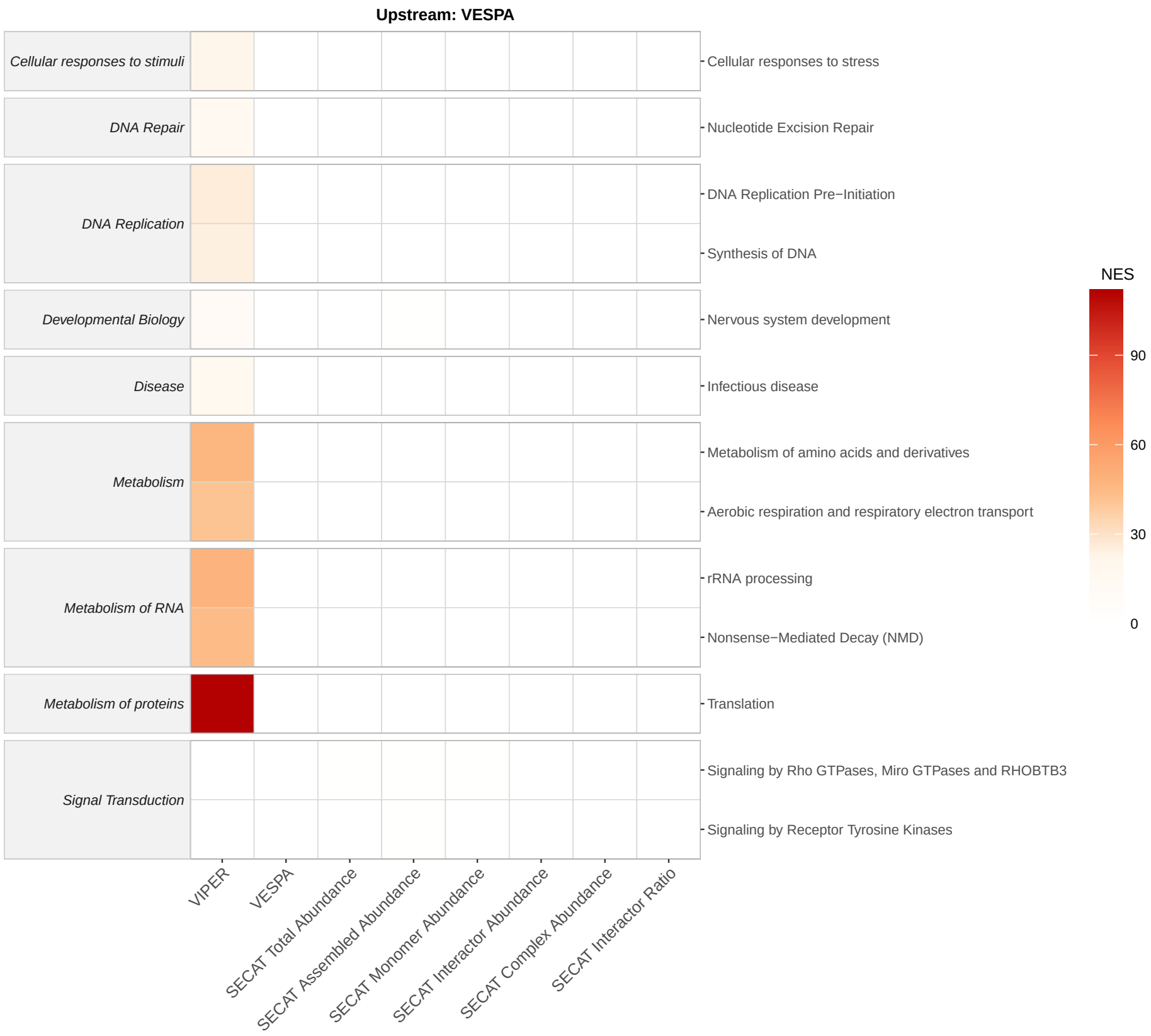

Appendix Figure S2. Downstream pathway NES heatmap for network diffusion with upstream VESPA (kinase/phosphatase signaling activity) layer. Rows: all Reactome L1 pathways significant (BH-adj. p-value < 0.05) in at least one upstream $\times$ downstream combination for this upstream layer, grouped by Reactome L0 top-level category. Columns: the eight downstream omics layers. Cell colour: normalised enrichment score (NES), capped at the per-upstream-layer maximum.

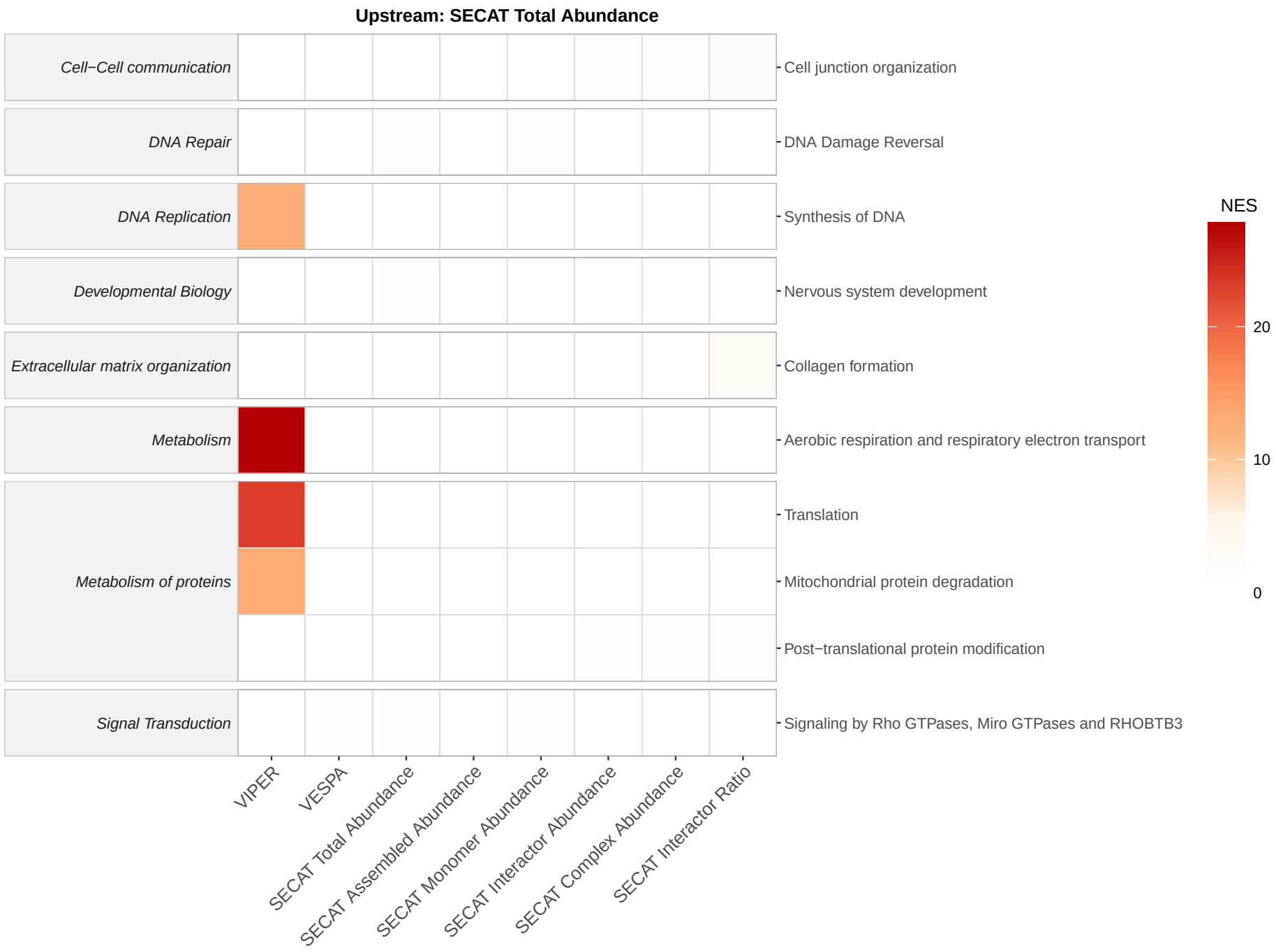

Appendix Figure S3. Downstream pathway NES heatmap for network diffusion with upstream SECAT total protein abundance layer. Rows: all Reactome L1 pathways significant (BH-adj. p-value < 0.05) in at least one upstream $\times$ downstream combination for this upstream layer, grouped by Reactome L0 top-level category. Columns: the eight downstream omics layers. Cell colour: normalised enrichment score (NES), capped at the per-upstream-layer maximum.

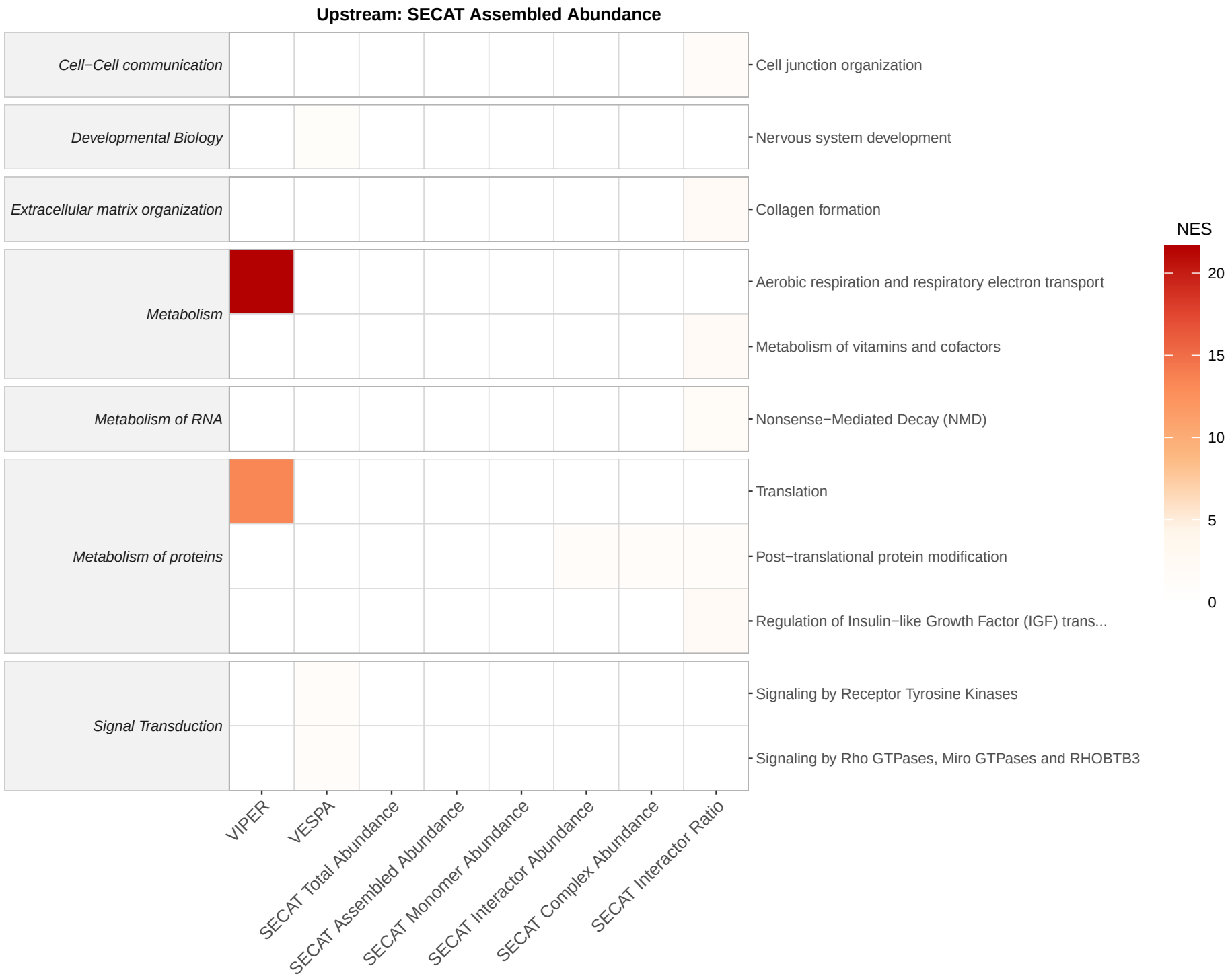

Appendix Figure S4. Downstream pathway NES heatmap for network diffusion with upstream SECAT assembled protein abundance layer. Rows: all Reactome L1 pathways significant (BH-adj. p-value < 0.05) in at least one upstream $\times$ downstream combination for this upstream layer, grouped by Reactome L0 top-level category. Columns: the eight downstream omics layers. Cell colour: normalised enrichment score (NES), capped at the per-upstream-layer maximum.

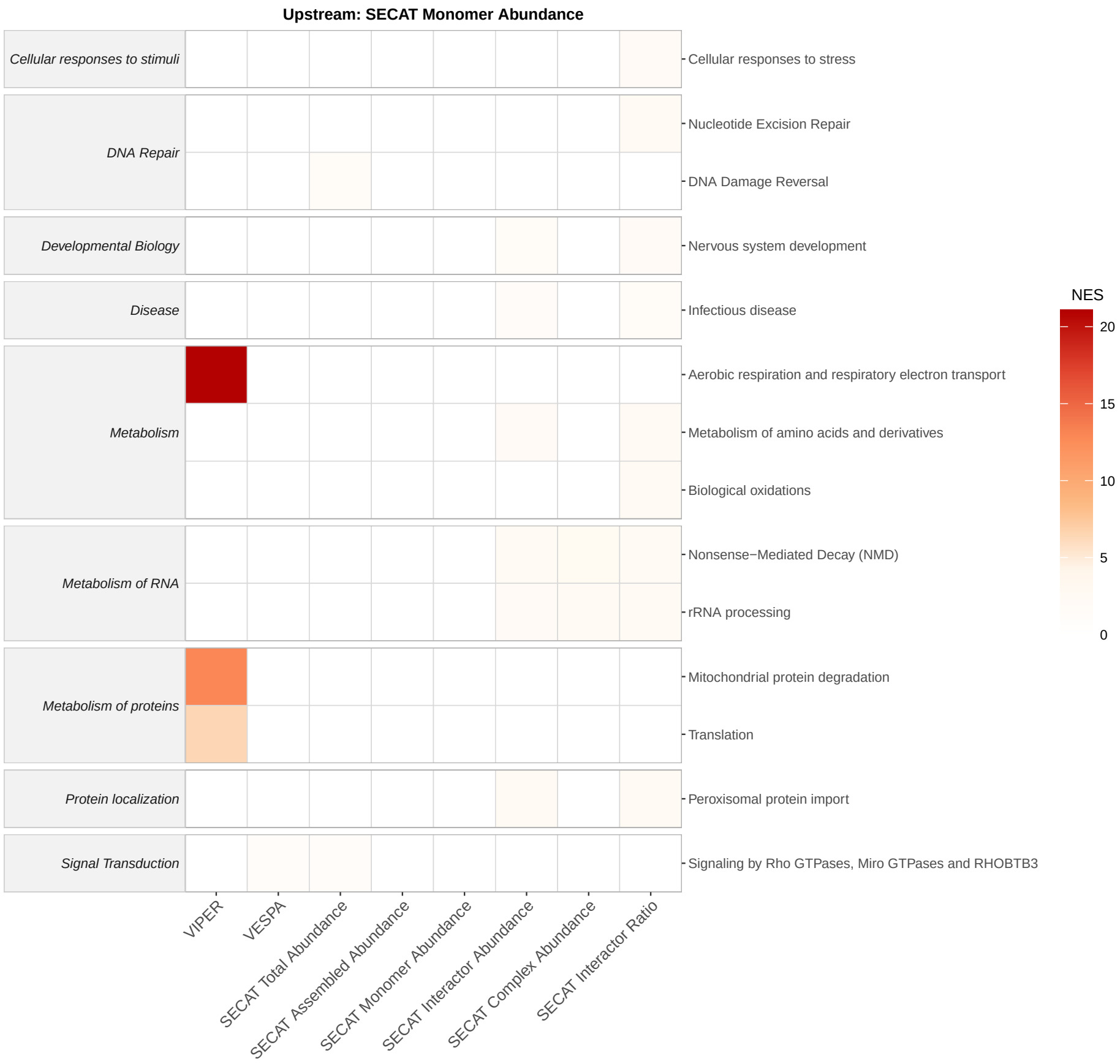

Appendix Figure S5. Downstream pathway NES heatmap for network diffusion with upstream SECAT monomer protein abundance layer. Rows: all Reactome L1 pathways significant (BH-adj. p-value < 0.05) in at least one upstream $\times$ downstream combination for this upstream layer, grouped by Reactome L0 top-level category. Columns: the eight downstream omics layers. Cell colour: normalised enrichment score (NES), capped at the per-upstream-layer maximum.

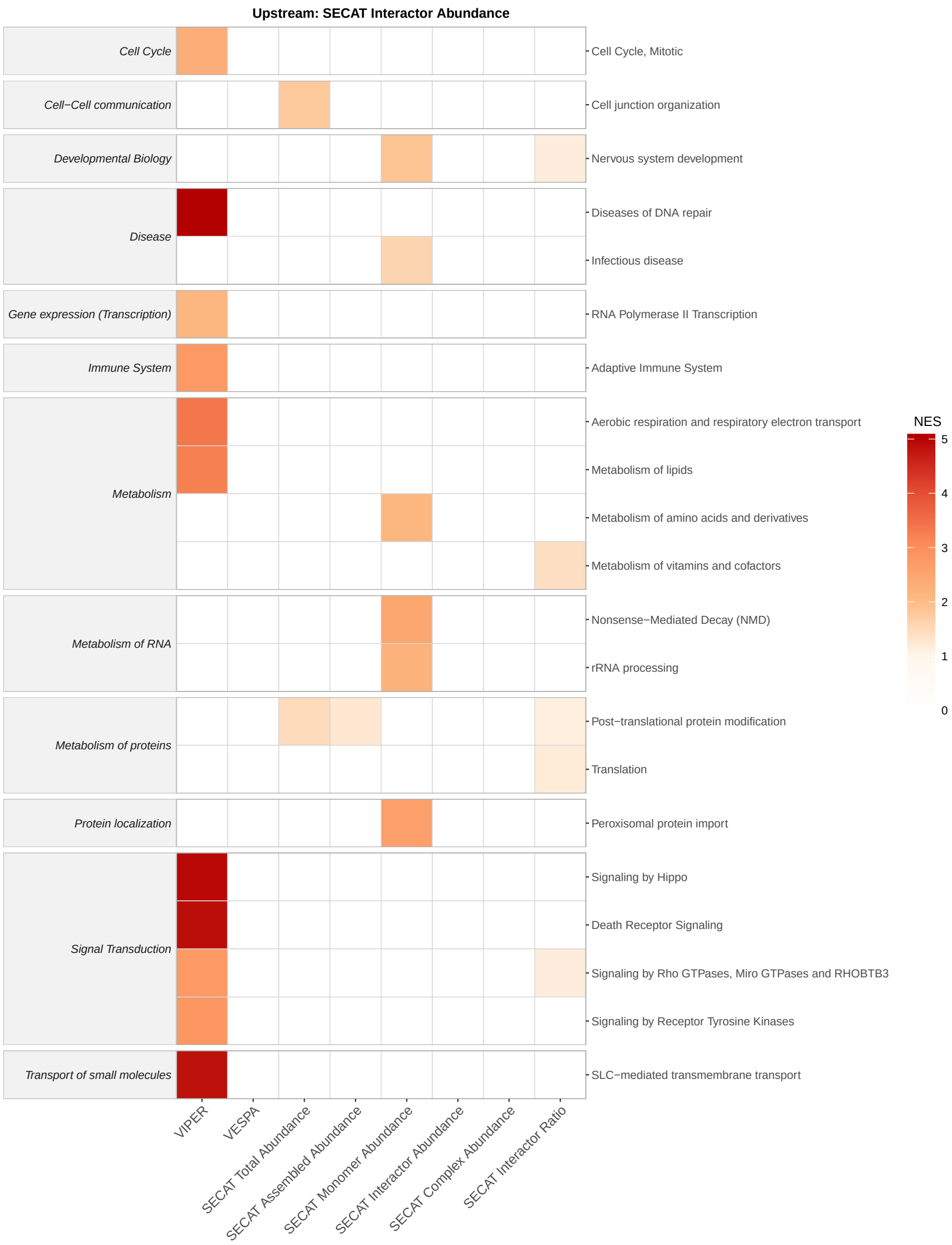

Appendix Figure S6. Downstream pathway NES heatmap for network diffusion with upstream SECAT interactor protein abundance layer. Rows: all Reactome L1 pathways significant (BH-adj. p-value < 0.05) in at least one upstream $\times$ downstream combination for this upstream layer, grouped by Reactome L0 top-level category. Columns: the eight downstream omics layers. Cell colour: normalised enrichment score (NES), capped at the per-upstream-layer maximum.

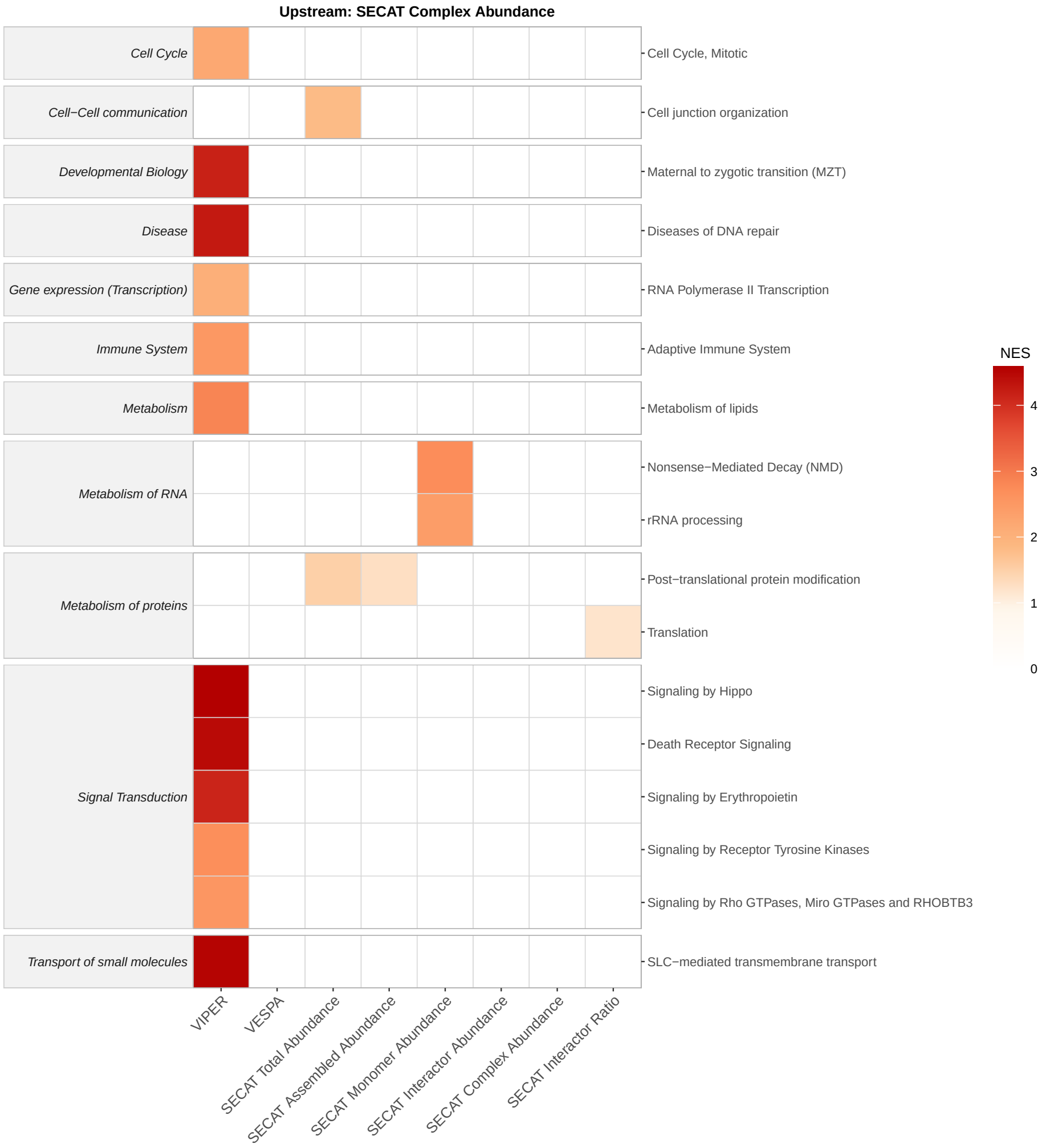

Appendix Figure S7. Downstream pathway NES heatmap for network diffusion with upstream SECAT complex protein abundance layer. Rows: all Reactome L1 pathways significant (BH-adj. p-value < 0.05) in at least one upstream $\times$ downstream combination for this upstream layer, grouped by Reactome L0 top-level category. Columns: the eight downstream omics layers. Cell colour: normalised enrichment score (NES), capped at the per-upstream-layer maximum.

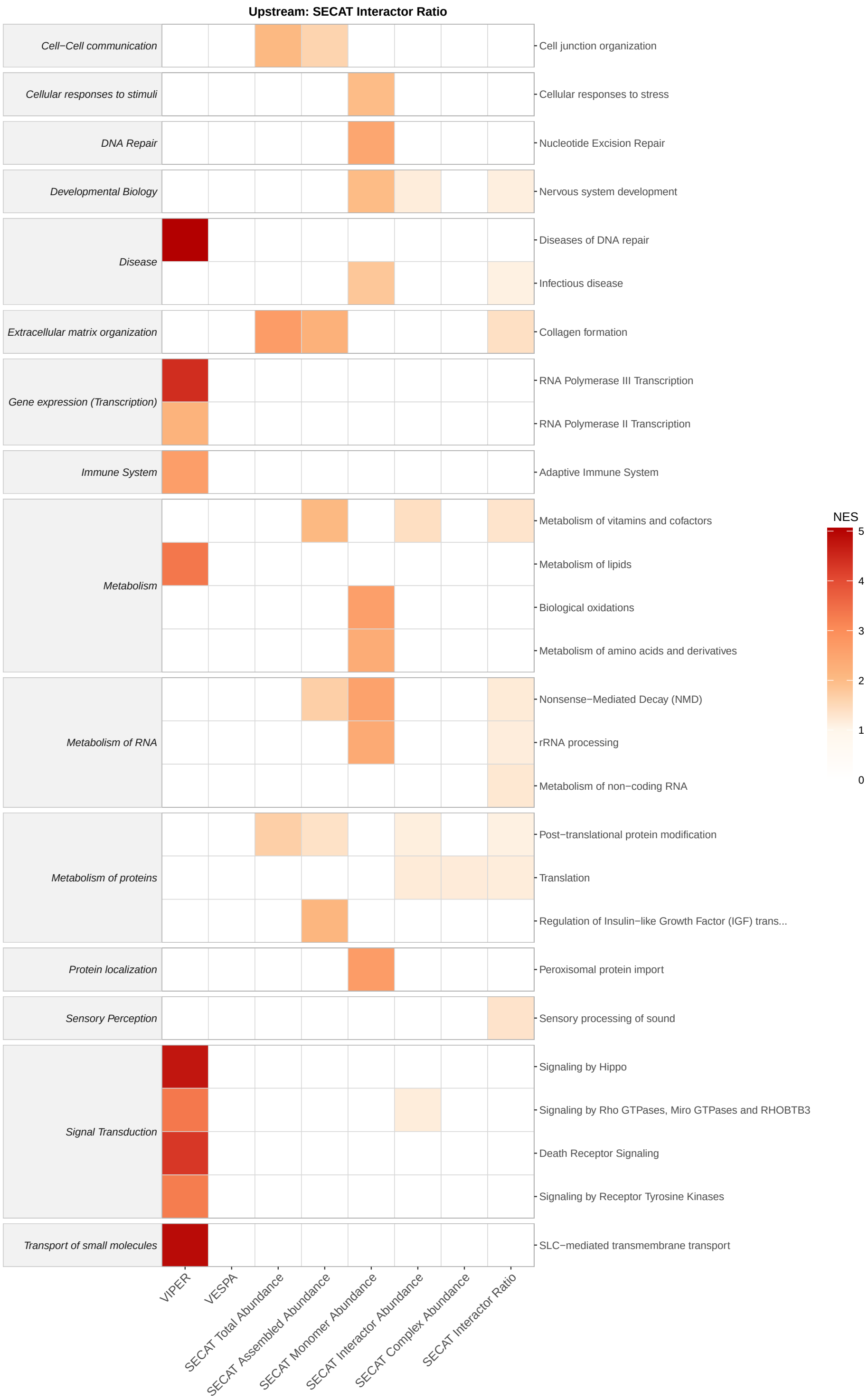

Appendix Figure S8. Downstream pathway NES heatmap for network diffusion with upstream SECAT interactor ratio layer. Rows: all Reactome L1 pathways significant (BH-adj. p-value < 0.05) in at least one upstream $\times$ downstream combination for this upstream layer, grouped by Reactome L0 top-level category. Columns: the eight downstream omics layers. Cell colour: normalised enrichment score (NES), capped at the per-upstream-layer maximum.

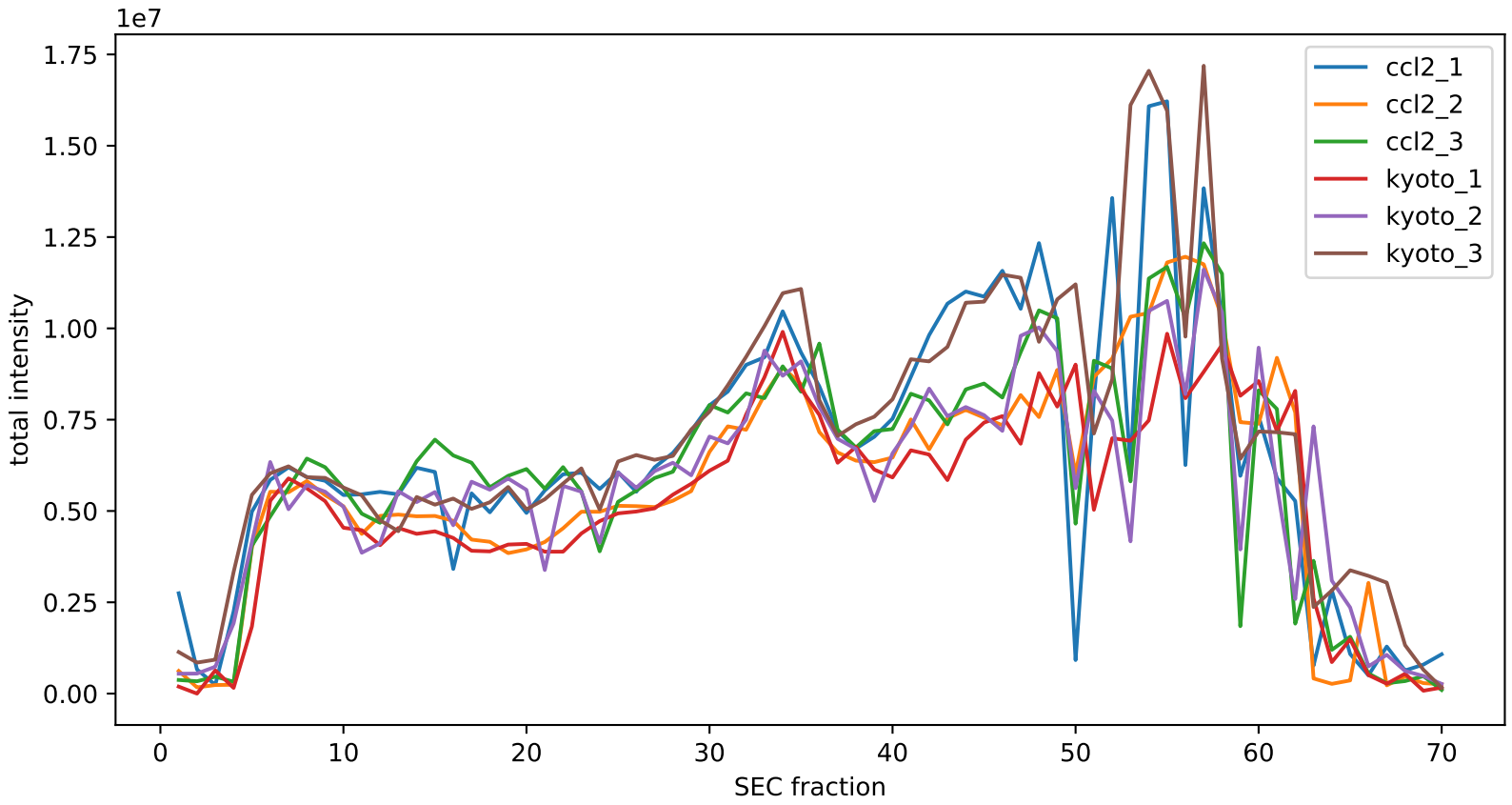

Appendix Figure S9. Summarized raw peptide precursor intensities over the SEC profile for the HeLa Kyoto vs. CCL2 data set. Each line represents one of $n = 3$ biological replicates per cell line (70 SEC fractions each).

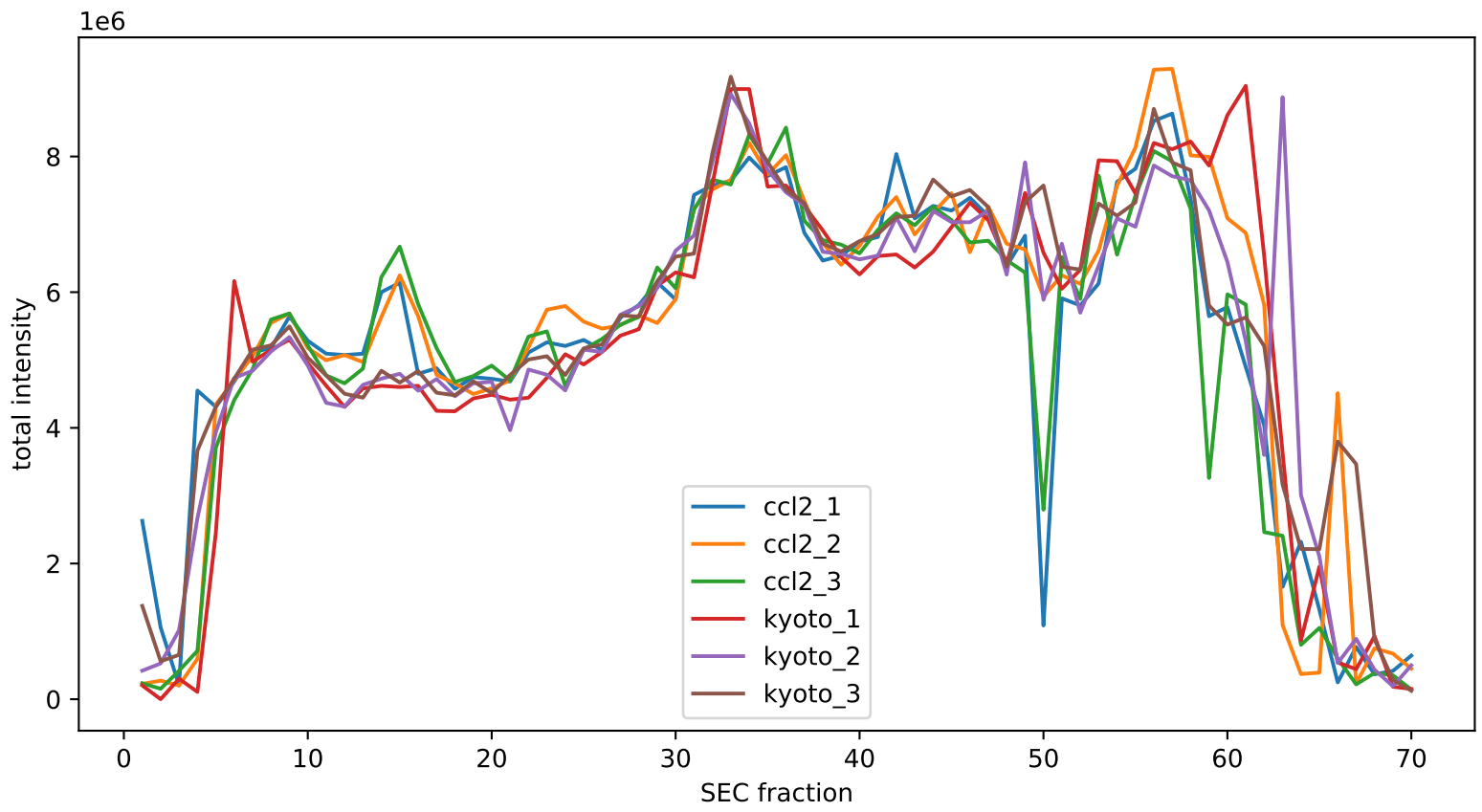

Appendix Figure S10. Summarized normalized peptide precursor intensities over the SEC profile for the HeLa Kyoto vs. CCL2 data set. Each line represents one of $n = 3$ biological replicates per cell line (70 SEC fractions each). Normalization was performed within SECAT as described in Methods.
