# Supplementary material for: Complex assembly and activity states as multifaceted protein attributes explaining phenotypic variability
Source: Mol Syst Biol. 2026 Jul 22;22(10):1551–77. doi: 10.1038/s44320-026-00228-3 (PMC13639060; doi:10.1038/s44320-026-00228-3)
Supplement: Supplementary file 3 [file 44320_2026_228_MOESM2_ESM.pdf]

Complex Assembly and Activity States as Multifaceted Protein Attributes Explaining Phenotypic Variability

George Rosenberger, Peng Xue, Isabell Bludau, Claudia Martelli, Evan Williams, Ben Collins, Andrea Califano, Yansheng Liu, and Ruedi Aebersold

Corresponding author(s): George Rosenberger (george@rosenberger.pro) , Ruedi Aebersold (aebersold@imsb.biol.ethz.ch), Yansheng Liu (yansheng.liu@yale.edu)

Review Timeline:

Submission Date:	26th Sep 25
Editorial Decision:	12th Nov 25
Revision Received:	24th Apr 26
Editorial Decision:	6th Jun 26
Revision Received:	9th Jun 26
Accepted:	11th Jun 26

Editor: Jingyi Hou

Transaction Report:

(Note: With the exception of the correction of typographical or spelling errors that could be a source of ambiguity, letters and reports are not edited. Depending on transfer agreements, referee reports obtained elsewhere may or may not be included in this compilation. Referee reports are anonymous unless the Referee chooses to sign their reports.)

12th Nov 2025

Manuscript Number: MSB-2025-13379
Title: Complex Assembly and Activity States as Multifaceted Protein Attributes Explaining Phenotypic Variability
Author: George Rosenberger
Peng Xue
Isabell Bludau
Claudia Martelli
Evan Williams
Ben Collins
Andrea Califano
Yansheng Liu
Ruedi Aebersold

Dear Dr. Rosenberger,

Thank you again for submitting your work to Molecular Systems Biology. I apologize for the somewhat slow process, which was due to delays in obtaining the referee reports. We have now heard back from the three reviewers who agreed to evaluate your study. As you will see below, the reviewers raise several critical concerns, which unfortunately preclude the publication of the study in its current form.

The reviewers expressed overlapping concerns regarding (i) the overall conceptual novelty and (ii) insufficient clarity and methodological detail. However, as the reviewers also acknowledged that the topic is important and relevant to the field, we would like to offer you the opportunity to revise your manuscript and address the points raised.

Without repeating all the points/comments listed below, the most fundamental issues that need to be convincingly addressed are the following:

1. In light of the comments from Reviewers #1 and #3 regarding novelty, please better place the study within the context of existing literature. Please also more clearly highlight the novel insights provided by your work and specify the precise knowledge gap it fills in the field.

2. Improve the clarity of the manuscript.

3. Reviewer #1 raised concerns about the model system used, whereas Reviewer #3 considered the general approach to be novel. Please respond to Reviewer #1's comments and discuss the potential limitations of your model system in a reasoned and balanced manner.

All other issues raised by the reviewers need to be satisfactorily addressed as well. As you may already know, our editorial policy allows in principle a single round of major revision so it is essential to provide responses to the reviewers' comments that are as complete as possible. Please feel free to contact me in case you would like to discuss in further detail any of the issues raised by the reviewers.

On a more editorial level, we would ask you to address the following issues:

- Please provide a .docx formatted version of the manuscript text (including legends for main figures, EV figures and tables). Please make sure that the changes are highlighted to be clearly visible.

- Please provide individual production quality figure files as .eps, .tif, .jpg (one file per figure).

-Please provide a .docx formatted letter INCLUDING the reviewers' reports and your detailed point-by-point responses to their comments. As part of the EMBO Press transparent editorial process, the point-by-point response is part of the Review Process File (RPF), which will be published alongside your paper.

-Please note that all corresponding authors are required to supply an ORCID ID for their name upon submission of a revised manuscript.

-We replaced Supplementary Information with Expanded View (EV) Figures and Tables that are collapsible/expandable online (see examples in http://msb.embopress.org/content/11/6/812). A maximum of 5 EV Figures can be typeset. EV Figures should be cited as 'Figure EV1, Figure EV2" etc... in the text and their respective legends should be included in the main text after the legends of regular figures.

Additional Tables/Datasets should be labeled and referred to as Table EV1, Dataset EV1, etc. Legends have to be provided in a separate tab in case of .xls files. Alternatively, the legend can be supplied as a separate text file (README) and zipped together with the Table/Dataset file.

For the figures and tables that you do NOT wish to display as Expanded View figures, they should be bundled together with their legends in a single PDF file called *Appendix*, which should start with a short Table of Content. Each legend should be below the corresponding Figure/Table in the Appendix. Appendix figures and tables should be referred to in the main text as: "Appendix Figure S1, Appendix Figure S2, Appendix Table S1" etc. See detailed instructions regarding expanded view here: https://www.embopress.org/page/journal/17444292/authorguide#expandedview.

-Before submitting your revision, primary datasets (and computer code, where appropriate) produced in this study need to be deposited in an appropriate public database (seehttp://msb.embopress.org/authorguide - dataavailability https://www.embopress.org/page/journal/17444292/authorguide#dataavailability).
Please remember to provide a reviewer password if the datasets are not yet public.
The accession numbers and database should be listed in a formal "Data Availability" section (placed after Materials & Method) that follows the model below (see also https://www.embopress.org/page/journal/17444292/authorguide#dataavailability). Please note that the Data Availability Section is restricted to new primary data that are part of this study.
Data availability
The datasets (and computer code) produced in this study are available in the following databases:
- RNA-Seq data: Gene Expression Omnibus GSE46843 (https://www.ncbi.nlm.nih.gov/geo/query/acc.cgi?acc=GSE46843)
- [data type]: [name of the resource] [accession number/identifier/doi] ([URL or identifiers.org/DATABASE:ACCESSION])
*** Note - All links should resolve to a page where the data can be accessed. ***

- At EMBO Press we ask authors to provide source data for the main manuscript figures. You will receive a separate email with instructions for providing source data with your revised manuscript, including how to upload and organize the files.
Additional information on source data and instruction on how to label the files are available

- Our journal encourages inclusion of *data citations in the reference list* to directly cite datasets that were re-used and obtained from public databases. Data citations in the article text are distinct from normal bibliographical citations and should directly link to the database records from which the data can be accessed. In the main text, data citations are formatted as follows: "Data ref: Smith et al, 2001". In the Reference list, data citations must be labeled with "[DATASET]". A data reference must provide the database name, accession number/identifiers and a resolvable link to the landing page from which the data can be accessed at the end of the reference. Further instructions are available at .

- We updated our journal's competing interests policy in January 2022 and request authors to consider both actual and perceived competing interests. Please review the policy https://www.embopress.org/competing-interests and update your competing interests if necessary.
Please use the heading "Disclosure statement and competing interests".

- All Materials and Methods need to be described in the main text using our 'Structured Methods' format. According to this format, the Methods section includes a Reagents and Tools Table (listing key reagents, experimental models, software and relevant equipment and including their sources and relevant identifiers) followed by a Methods and Protocols section describing the methods, ideally using a step-by-step protocol format. The aim is to facilitate adoption of the methodologies across labs.

Please download and fill our Reagents and Tools Table template (.docx), which you can find in our author guidelines: https://www.embopress.org/page/journal/17444292/authorguide#structuredmethods.

When submitting your revised manuscript, please do not include the Reagents and Tools Table in the Methods section of the manuscript but upload it as a separate file choosing the file type "Reagent Table".

An example of a Method paper with Structured Methods can be found here: https://www.embopress.org/doi/10.15252/msb.20178071.

-Regarding data quantification:
Please ensure to specify the name of the statistical test used to generate error bars and P values, the number (n) of independent experiments (please specify technical or biological replicates) underlying each data point and the test used to calculate p-values in each figure legend. Discussion of statistical methodology can be reported in the materials and methods section, but figure legends should contain a basic description of n, P and the test applied.
Graphs must include a description of the bars and the error bars (s.d., s.e.m.).
Please also include scale bars in all microscopy images.

- Please provide a "standfirst text" summarizing the study in one or two sentences (approximately 250 characters, including space), three to four "bullet points" highlighting the main findings and a "synopsis image" (550px width and 400-600 px height,

PNG format) to highlight the paper on our homepage.
Here are a couple of examples:
https://www.embopress.org/doi/10.15252/msb.20199356
https://www.embopress.org/doi/10.15252/msb.20209475
https://www.embopress.org/doi/10.15252/msb.209495

When you resubmit your manuscript, please download our CHECKLIST (https://www.embopress.org/pb-assets/embo-site/EMBO%20Press%20Author%20Checklist-1642513524327.xlsx) and include the completed form in your submission. *Please note* that the Author Checklist will be published alongside the paper as part of the transparent process (https://www.embopress.org/page/journal/17444292/authorguide#transparentprocess).

If you feel you can satisfactorily deal with these points and those listed by the referees, you may wish to submit a revised version of your manuscript. Please attach a covering letter giving details of the way in which you have handled each of the points raised by the referees. A revised manuscript will be once again subject to review and you probably understand that we can give you no guarantee at this stage that the eventual outcome will be favorable.

We look forward to receiving the revised manuscript soon.

Sincerely,
Jingyi

Jingyi Hou, PhD
Senior Editor
Molecular Systems Biology

We realize that it is difficult to revise to a specific deadline. In the interest of protecting the conceptual advance provided by the work, we recommend a revision within 3 months (10th Feb 2026). Please discuss the revision progress ahead of this time with the editor if you require more time to complete the revisions. Use the link below to submit your revision:

https://msb.msubmit.net/cgi-bin/main.plex

*** PLEASE NOTE *** As part of the EMBO Press transparent editorial process initiative (see our Editorial at https://dx.doi.org/10.1038/msb.2010.72), Molecular Systems Biology publishes online a Review Process File with each accepted manuscripts. This file will be published in conjunction with your paper and will include the anonymous referee reports, your point-by-point response and all pertinent correspondence relating to the manuscript. If you do NOT want this File to be published, please inform the editorial office at contact@molsystbiol.org within 14 days upon receipt of the present letter.

Reviewer #1:

This manuscript postulates-and seeks to demonstrate-that protein activity is more closely linked to cellular phenotypic traits than protein abundance. To explore this, the authors incorporate functionally relevant protein attributes, including transcription factor-gene regulatory networks, kinase-substrate relationships, and protein-protein interaction networks. These layers are analyzed in an integrated manner to provide mechanistic insights into cellular phenotypes. The authors further demonstrate that this approach can reveal phenotypic differences among a panel of distinct HeLa cell lines.

The rationale of this paper makes sense: protein abundance, as typically measured in proteomic experiments, represents the cumulative outcome of transcriptional and (post-)translational regulatory mechanisms that shape cellular phenotypes. This concept is intuitive and well supported by existing literature. It is therefore somewhat surprising that the authors begin by attempting to demonstrate this experimentally through an analysis that they claim reveals a buffering effect of protein complexes (as opposed to monomers) against copy number variation and transcriptional heterogeneity. Unfortunately, this analysis appears weak, its generalized conclusion is open to alternative interpretations (as detailed below), and it provides a less solid foundation for the paper than would seem necessary given the prior evidence already available.

The remainder of the manuscript presents an extensive set of computational analyses that derive correlations across multiple data layers and identify network properties associated with specific phenotypic traits (such as cell doubling or susceptibility to

infection), or that differ more generally among the cell lines studied. However, these sections mainly comprise long lists of proteins and processes emerging from the computational analyses, without it becoming clear how these findings demonstrate the value of the proposed integrative approach. This limitation stems largely from the absence of a ground truth in the model system, which prevents a meaningful assessment of the method's performance or interpretability.

As a result, it remains unclear what concrete biological insights can be gained from the study, particularly regarding the specific behavior or phenotypic diversity of the HeLa cell line panel. The main takeaway seems to be that integrating multiple complementary data types provides more information than protein abundance alone-a conclusion that, while reasonable, is conceptually evident from the outset. Overall this leaves a very unfulfilling impression, making it is hard to see the manuscript as a strong candidate for this journal.

Specific comments:
1. The authors observe that "single-state" monomeric and assembled fractions show similar correlations with CNV and mRNA abundance, whereas this relationship diverges for "paired-state" proteins. From this, they conclude that "proteins in assembled conformations are buffered from CNV and mRNA abundance variation compared to their monomers" (p. 4). I find this to be a rather generalized conclusion drawn from Pearson correlation coefficients that are relatively low to begin with, and that differ by only about 0.1. Moreover, multiple layers of regulation-at the levels of RNA, protein stability, post-translational modifications, or protein-protein interactions (or combinations thereof)-could equally explain minor shifts in correlation values. While the authors partly recognize this, this may be interpreted as "buffering" in some specific instances, but alternative regulatory mechanisms may underlie the observations for other proteins. In addition, the protein classes shown in Fig. 2c and d include variable numbers of proteins, raising the concern that modest deviations in smaller datasets (e.g., the top-left panel in Fig. 2c) may disproportionately affect the calculated Pearson correlations. The authors should acknowledge this broader perspective to prevent over-interpretation of the data. Otherwise, the conclusion that "protein complexes buffer against heterogeneity in mRNA abundance" risks leaving readers with an appealing yet insufficiently substantiated message-one that rests largely on a single statistical measure (i.e. Pearson correlation).

2. The various analyses presented in the manuscript yield a large number of proteins, interactions and functional categories that are reported to differ between the analyzed cell lines (pages 8-17). However, it is unclear which of these findings are robust or even biologically meaningful. The core issue is that the cell lines differ in unknown ways, leaving no ground truth to assess the validity or performance of the analyses conducted at the different data levels. A more informative approach would have been to use a model system that includes a defined perturbation with a known biological effect-potentially coupled with the question of how the same perturbation elicits context-dependent (i.e., cell line-specific) downstream responses.

3. Several figures contain an overwhelming amount of information, making it difficult to interpret their main message, even when guided by the authors' descriptions or conclusions (e.g., Fig. 3d). Other figures attempt to integrate so many layers of data that it becomes challenging-at least for this reviewer-to grasp the key message, whether at the detailed or overall level (Figs. 4 and 5).

Reviewer #2:

The manuscript "Complex Assembly and Activity States as Multifaceted Protein Attributes Explaining Phenotypic Variability" by Rosenberger et al. describes a comprehensive systems-level analysis of protein complex dynamics and activity states. As a model system, authors use a well-characterized panel of 14 HeLa cell lines, known for their phenotypic heterogeneity in cell doubling time and susceptibility to Salmonella Typhimurium infection. Introducing a multi-omics framework that integrates SEC-SWATH-MS, network inference tools (SECAT, VIPER, VESPA), and network diffusion analysis (TieDIE), authors demonstrate that protein attributes beyond abundance, such as complex assembly and inferred protein activities, provide deeper functional insights into mechanisms underlying molecular phenotypes.

Overall, this is excellent and well-structured study that builds on prior work by the authors and contributes meaningfully to the field of molecular systems biology. The integration of proteomic, transcriptomic, and phosphoproteomic data to infer functional protein states and align elucidated functional dependencies with phenotypes is compelling. I support publication pending minor text revisions.

Comments:
1. The font size in Figures 3, 4, 5, 7 and Supplementary Figures 5-12 is too small and difficult to read. Annotations on Figure 5 panel A appear to be blurred. Increasing the font size for axis labels, legends, and annotations would significantly improve the clarity and accessibility of otherwise excellently presented data.

2. To strengthen the biological impact of the findings, providing or proposing as future direction some experimental validation of a few computational inferences would be beneficial. For example, could the differential activity of LIMK1 or the reconfigured SCF complex (FBXO2) be confirmed in those HeLa cell lines using orthogonal approaches like kinase assay or co-immunoprecipitation?

3. In the section on "Association of protein abundance and protein complex/network dysregulation with phenotypic variability" it would be helpful to briefly reiterate which HeLa cell lines are susceptible to infection and which exhibit faster or slower doubling times. This would help re-orient the reader when interpreting the differential analyses.

Reviewer #3:

The manuscript submitted by Rosenberger et al describes a novel approach of omic data integration by which transcriptome, proteome, phosphoproteome, and interactome data are used to infer protein activity states. These protein activity states along with protein complex and network states are then used to generate hypotheses about molecular mechanisms driving two different phenotypes, doubling time and infection susceptibility, of two HeLa cell lines. Most of the data has been generated before while protein complex measurements were done as part of this study. They authors find that inferences of complex and network dysregulation are more informative on potential molecular mechanisms and connecting genotype to phenotype than protein abundances alone. The authors use their novel multiomic data integration approach to show that protein complex stoichiometry is a cellular buffering mechanisms against CNVs and transcriptional variability.

The manuscript addresses an important problem in biology in showing that genotype-to-phenotype relationships can only be understood and predicted if we incorporate the molecular interaction networks that mediate these relationships. It is impossible to predict phenotypes from the cellular parts list, the abundances of transcripts and proteins, alone. To make this point, the authors use an interesting model system of two divergent HeLa cell lines that have been extensively profiled in previous studies. However, my main critique point to this manuscript is that at many places it is not understandable. That is because a lot of vague technical terms are used that are not defined. A lot of software is used that is highly specialized (unknown to most readers including the reviewer) and not properly introduced, and as a consequence it remains largely unclear how authors came to the conclusions that they state in the manuscript.

For example: What are the tools SEC-SWATH-MS, VESPA, VIPER, SECAT, STELLA, DeMAND, TieDIE do, conceptually? What do you mean by protein activity? It seems from the manuscript that at different places this means different things?

Here is an example of a sentence that is unclear (page 11):
"Comparing the sets of differentially active proteins between the three functional levels tested by the algorithms (p. adj < 0.05), illustrates the heterogeneity of protein function, as well as potential confounding technical biases between the different types of analysis." What are the three functional levels? What are the tested algorithms? What do you mean by heterogeneity of protein function? Which different types of analyses? Which technical biases?

Here is another example (page 13):
"Therefore, our goal was to assess whether the context-specific PPI networks and SECAT-informed quantitative features at the different levels of information provided by SECAT could be used to elucidate molecular mechanisms differentiating CCL2 and Kyoto HeLa cells." What are the SECAT-informed quantitative features? What are the different levels of information provided by SECAT?

I encourage the authors to check the entire manuscript for these kinds of overly general and cryptic statements and improve the clarity of the manuscript.

Other major points:
The introduction lacks more information on the previous study that generated the molecular signatures of the HeLa cell lines and the findings made since the authors refer to it at multiple places in the manuscript. It would also be nice to have more information provided as to why these profiled HeLa cell lines are a good model system for this study.

The manuscript lacks references to previous work on multiomic data integration approaches, i.e. such as MOFA. What are the advantages and disadvantages of the newly proposed versus previously existing approaches?

One of the key findings reported in this manuscript is that protein complex assemblies enable buffering against protein copy number variation. This to the best of my understanding is not new. Other studies have reported that orphan complex subunits, so those that cannot be incorporated into protein complex assemblies because there is not enough of the other complex subunits, are being rapidly degraded as a consequence. How is your analysis and how are your findings different from this previous work (i.e. reviewed in these articles: https://www.cell.com/trends/cell-biology/fulltext/S0962-8924(22)00038-1, https://pmc.ncbi.nlm.nih.gov/articles/PMC6624128)?

On page 7 the authors describe how they developed and used STELLA to generate cell line-specific PPI networks by integrating publicly available datasets on PPIs with protein abundance profiles obtained from this work. They then use the tool DeMAND to identify proteins with dysregulated interactions (what do you precisely mean by this? interactions that are absent or present in the two cell lines?) to quantify protein complex and network dysregulation (what do you mean by dysregulation more precisely)?

If PPI dysregulation is inferred from protein abundance differences in the two cell lines, then it remains unclear how the authors can later on conclude from this data that PPI dysregulation is more powerful than protein abundances in understanding molecular mechanisms. I agree that PPI data is key in studying molecular mechanisms but protein abundance is also key for inferring differential PPIs between cellular states in their approach, right? So, it is misleading how the authors undermine the importance of protein abundance in their study.

Furthermore, the authors then find that these PPI, complex and network dysregulations are not more powerful than the other data modalities in identifying "molecular determinants that are linked to the phenotypes" (what do you actually precisely mean by molecular determinants?). To still show that PPI, complex and network dysregulation are more powerful, the authors use GO cellular component (CC) annotation enrichments. I wonder to which extent this gives a favour to these inferred data modalities because the GO CC terms are precisely describing protein complexes. How would the analysis look like when using GO biological process or molecular function terms instead?

Towards the end of the results section the authors describe observations from the integrative analyses that might underlie the different phenotypes observed in both HeLa cell lines. However, I miss figures and analyses that show how these findings could not have been made by using protein abundance profiles alone.

The current figures also remain largely unclear because the figure legends lack sufficient information to understand what readers are looking at. For example, figure 6, what are upstream and downstream layers? What is the quantification on the x-axis (numbers ranging from 0-5 labelled Significant subpathways)? Many plots have NES as x-axis label but I couldn't find a place in the manuscript where the abbreviation NES is explained. I also don't understand figure 3d. What am I looking at? How was the relative feature importance determined and how can I see that complex and network dysregulation related the different gene sets better with the two phenotypes? What are the correlation coefficients?

Complex Assembly and Activity States as Multifaceted Protein Attributes Explaining Phenotypic Variability

George Rosenberger[1,10,†], Peng Xue[2,11,†], Isabell Bludau[2,12], Claudia Martelli[2,10], Evan Williams[2,13], Ben C. Collins[2,14], Andrea Califano[1,3,4,5,6,7], Yansheng Liu[8,9], Ruedi Aebersold[2]

[1] Department of Systems Biology, Columbia University Irving Medical Center, New York, NY, USA
[2] Institute of Molecular Systems Biology, ETH Zurich, Zurich, Switzerland
[3] Department of Medicine, Columbia University Irving Medical Center, New York, NY, USA
[4] Department of Biochemistry & Molecular Biophysics, Columbia University Irving Medical Center, New York, NY, USA
[5] Herbert Irving Comprehensive Cancer Center, Columbia University Irving Medical Center, New York, NY, USA
[6] Department of Biomedical Informatics, Columbia University Irving Medical Center, New York, NY, USA
[7] Chan Zuckerberg Biohub New York, New York, NY, USA
[8] Yale Cancer Biology Institute, Yale University, West Haven, CT, USA
[9] Department of Pharmacology, Yale University School of Medicine, New Haven, CT, USA
[10] Present address: Bruker Switzerland AG, Fällanden, Switzerland
[11] Present address: Guangzhou National Laboratory, Guangzhou, China
[12] Present address: Department of Neuropathology, University Hospital Heidelberg, Heidelberg, Germany
[13] Present address: Luxembourg Centre for Systems Biomedicine, University of Luxembourg, Esch-Belval, Luxembourg
[14] Present address: School of Biological Sciences, Queen's University of Belfast, UK

† Equal Contribution
Correspondence to: george@rosenberger.pro, yansheng.liu@yale.edu and aebersold@imsb.biol.ethz.ch

Point-by-point Response to Reviewers

We thank the editor and the reviewers for their constructive feedback and the opportunity to revise our manuscript. We have carefully considered all comments and have performed substantial revisions to address the concerns raised. The most significant changes include:

- A new three-pronged validation framework (DepMap CRISPR essentiality, HPA subcellular localization, Let7 perturbation) providing orthogonal ground-truth evidence for our inferred protein activities (**Figure 3**).
- Direct evidence for complex-based buffering of gene dosage variation, using physical separation of monomeric and assembled protein fractions (**Figure 2**).
- Linking the protein complex buffering hypothesis to cellular fitness via DepMap essentiality analysis ($p = 1.35 \times 10^{-29}$; **Figure 2b**).
- **Box 1** in the Introduction defining all key tools and concepts (SECAT, VIPER, VESPA, diffuStats, and "Protein Activity").
- A new biological overview schematic (**Figure 7**) synthesizing the mechanistic findings and connecting molecular mechanisms to phenotypic divergence.
- Improved figures (**Figures 2–7**), increased font sizes, expanded figure legends, and restructured Methods into MSB Structured Methods format.
- Explicit positioning relative to prior work (including MOFA) and a detailed limitations paragraph, with a direct comparison of mechanistic vs. latent factor models.
- A substantially rewritten Abstract and Introduction emphasizing the three key findings: (1) direct evidence for complex-based buffering, (2) orthogonal validation of inferred activities, and (3) mechanistic insights inaccessible from abundance data alone.
- Removal of STELLA, DeMAND, and TieDIE from the analysis pipeline, replaced by a streamlined SECAT + diffuStats workflow. The core biological conclusions remain unchanged.
- Complete recomputation of all analyses using the latest release versions of all software packages and reference databases (including ARACNe/aracne.network, CORUM, Reactome, DepMap, and all Bioconductor dependencies). This ensures full reproducibility with current tool versions and accounts for minor numerical differences relative to the original submission, while the biological signals and conclusions are conserved throughout.

Below is our point-by-point response.

Editor Comments

Dear Dr. Rosenberger,

Thank you again for submitting your work to Molecular Systems Biology. I apologize for the somewhat slow process, which was due to delays in obtaining the referee reports. We have now heard back from the three reviewers who agreed to evaluate your study. As you will see below, the reviewers raise several critical concerns, which unfortunately preclude the publication of the study in its current form.

The reviewers expressed overlapping concerns regarding (i) the overall conceptual novelty and (ii) insufficient clarity and methodological detail. However, as the reviewers also acknowledged that the topic is important and relevant to the field, we would like to offer you the opportunity to revise your manuscript and address the points raised.

Without repeating all the points/comments listed below, the most fundamental issues that need to be convincingly addressed are the following:

1. In light of the comments from Reviewers #1 and #3 regarding novelty, please better place the study within the context of existing literature. Please also more clearly highlight the novel insights provided by your work and specify the precise knowledge gap it fills in the field.

2. Improve the clarity of the manuscript.

3. Reviewer #1 raised concerns about the model system used, whereas Reviewer #3 considered the general approach to be novel. Please respond to Reviewer #1's comments and discuss the potential limitations of your model system in a reasoned and balanced manner.

All other issues raised by the reviewers need to be satisfactorily addressed as well. As you may already know, our editorial policy allows in principle a single round of major revision so it is essential to provide responses to the reviewers' comments that are as complete as possible. Please feel free to contact me in case you would like to discuss in further detail any of the issues raised by the reviewers.

On a more editorial level, we would ask you to address the following issues: providing a .docx formatted version of the manuscript text; individual production quality figure files; a .docx formatted point-by-point response letter (for the Review Process File); ORCID IDs for all corresponding authors; reformatting supplementary materials as Expanded View (EV) and Appendix figures; depositing primary datasets in public databases; providing source data for main figures; including data citations in the reference list; updating competing interests; restructuring Methods into "Structured Methods" format with a Reagents and Tools Table; ensuring statistical reporting (test names, n, error bars) in all figure legends; and providing a standfirst text, bullet points, and synopsis image.

Sincerely,
Jingyi Hou, PhD
Senior Editor, Molecular Systems Biology

Response: We have revised the manuscript to explicitly address these three fundamental issues:

1. **Context & Novelty:** The Introduction now explicitly positions our work within the context of existing literature on protein buffering and multi-omics integration, including a direct comparison with latent factor models such as MOFA. The buffering analysis emphasizes our *direct physical measurement* of monomer vs. assembled states via SEC-SWATH-MS, contrasting with previous inferential studies. We further demonstrate that this buffering is a fitness-preserving mechanism for essential machinery ($p = 1.35 \times 10^{-29}$, Wilcoxon rank-sum test; Figure 2b).

2. **Clarity:** We have added **Box 1** ("Integrated Multi-Omics Toolkit for Inferring Protein Functional States") to the Introduction, clearly defining all key concepts ("Protein Activity" as catalytic, regulatory, or structural output) and tools (SECAT, VIPER, VESPA, diffuStats). We have also added a new biological overview figure (**Figure 7**) to synthesize the findings into a clear narrative, and we have restructured the manuscript to improve readability throughout. Technical jargon has been systematically replaced or defined on first use.

3. **Model System & Validation:** To address the lack of a "ground truth," we performed a new three-pronged validation (Results section "Multi-omic inference of differential protein activities and complex states"; **Figure 3**) using orthogonal datasets: (i) DepMap CRISPR gene dependency scores for cervical cancer cell lines, (ii) HPA subcellular localization enrichment, and (iii) Let7 microRNA perturbation correlations with infection phenotype. This validation empirically demonstrates that our methods identify functional drivers and essential structural components that abundance analysis misses. We have also added a dedicated limitations paragraph in the Discussion addressing the single-cell-line-panel design and the constraints of SEC-SWATH-MS.

Reviewer #1

> This manuscript postulates—and seeks to demonstrate—that protein activity is more closely linked to cellular phenotypic traits than protein abundance. To explore this, the authors incorporate functionally relevant protein attributes, including transcription factor–gene regulatory networks, kinase–substrate relationships, and protein–protein interaction networks. These layers are analyzed in an integrated manner to provide mechanistic insights into cellular phenotypes. The authors further demonstrate that this approach can reveal phenotypic differences among a panel of distinct HeLa cell lines.
>
> **R1.1:** The rationale of this paper makes sense: protein abundance, as typically measured in proteomic experiments, represents the cumulative outcome of transcriptional and (post-)translational regulatory mechanisms that shape cellular phenotypes. This concept is intuitive and well supported by existing literature. It is therefore somewhat surprising that the authors begin by attempting to demonstrate this experimentally through an analysis that they claim reveals a buffering effect of protein complexes (as opposed to monomers) against copy number variation and transcriptional heterogeneity. Unfortunately, this analysis appears weak, its generalized conclusion is open to alternative interpretations (as detailed below), and it provides a less solid foundation for the paper than would seem necessary given the prior evidence already available.

Response: We acknowledge the reviewer's point that the concept of protein buffering is present in the literature (including our own 2019 study). However, previous studies largely *inferred* buffering through global correlation mismatches between transcriptome and proteome, or from pulsed-SILAC measurements of total protein pools. The novelty of our buffering analysis lies in the **direct physical measurement** of this phenomenon. By utilizing SEC-SWATH-MS, we physically separated monomeric from assembled fractions of the *same* proteins, enabling an internal control where translational and environmental factors are identical for both fractions.

To strengthen this section as requested, we have made the following changes:

1. The Introduction now frames the knowledge gap, stating that "protein complex profiling was not performed, leaving open how complex assembly directly buffers gene dosage variation in this system." The Results section then opens with the explicit claim that "previous studies largely inferred this buffering from global correlation analyses, pulsed-SILAC measurements, or bulk protein pools" and that we "provide direct physical evidence for the complex-based buffering hypothesis by resolving the distinct behavior of monomeric versus assembled fractions of the same proteins." We cite the relevant reviews on orphan subunit quality control (Juszkiewicz & Bhatt, 2018; Pla-Prats & Bhatt, 2022; Taggart et al., 2020).

2. We clarified in the text the "paired state" design: proteins detected in both monomeric and assembled fractions serve as their own internal control. The assembled fraction (Spearman's ρ with CNV: 0.198) is significantly less correlated than the monomeric

fraction (ρ: 0.322; $p = 4.92 \times 10^{-4}$) from the same proteins. For mRNA, the same pattern holds ($\rho_{\text{assembled}}$: 0.484 vs. ρ_{monomer}: 0.574; $p = 1.09 \times 10^{-3}$). This internal control minimizes the "alternative interpretations," as both fractions are subject to the same regulatory environment, yet only the assembled fraction is buffered.

3. We performed a new analysis linking this buffering to cellular fitness (**Figure 2b**). Proteins detected in paired state (both monomer and assembled fractions) are significantly enriched for essential genes compared to proteins found exclusively as monomers ($p = 1.35 \times 10^{-29}$, Wilcoxon rank-sum test; $n_{\text{paired}} = 3{,}439$; $n_{\text{single monomer}} = 654$). This supports the hypothesis that stoichiometry buffering is an active mechanism preserving essential cellular machinery.

R1.2: The remainder of the manuscript presents an extensive set of computational analyses that derive correlations across multiple data layers and identify network properties associated with specific phenotypic traits (such as cell doubling or susceptibility to infection), or that differ more generally among the cell lines studied. However, these sections mainly comprise long lists of proteins and processes emerging from the computational analyses, without it becoming clear how these findings demonstrate the value of the proposed integrative approach. This limitation stems largely from the absence of a ground truth in the model system, which prevents a meaningful assessment of the method's performance or interpretability.

Response: This is a critical and fair point. To address the fundamental concern of a missing ground truth, we have added a **new validation framework** within the Results section "Multi-omic inference of differential protein activities and complex states," using three orthogonal datasets that serve as independent benchmarks (**Figure 3**):

1. **Functional Ground Truth (DepMap Essentiality):** We mapped the top 100 differentially active proteins per method to CRISPR gene dependency scores from DepMap (Public 25Q3, cervical cancer cell lines) to assess whether our methods identify genes essential for cell viability (Figure 3a).
    - **Result:** SECAT interactor ratio features mapped to genes with a median dependency probability of **0.52**, an **8.7-fold enrichment** over differentially abundant proteins (median 0.06; $p = 6.4 \times 10^{-5}$, Wilcoxon rank-sum test). This demonstrates that protein complex stoichiometry changes pinpoint core essential cellular machinery (ribosomes, proteasomes, spliceosomes) that abundance analysis misses.
    - SECAT complex abundance also showed elevated essentiality (median 0.10), while VESPA kinases showed significantly *lower* dependency ($p = 7.8 \times 10^{-4}$), consistent with their role as upstream regulators rather than structural housekeeping components.

2. **Spatial Coherence (HPA Localization):** We assessed whether the top features per method map to coherent subcellular compartments using fast gene set enrichment analysis (fgsea) against HPA annotations (Figure 3b).
    - **Result:** SECAT complex abundance features were significantly enriched for 5 compartments (BH-adj. $p < 0.05$): focal adhesion sites, actin filaments, plasma membrane, vesicles, and mitochondria, precisely the structures implicated in the invasive phenotype. Protein abundance identified only nucleolar proteins.
3. **Phenotypic Relevance (Let7 perturbation):** We leveraged the Let7 microRNA perturbation experiment from our 2019 study, where the same HeLa cell lines were treated with Let7 mimics. We tested whether the molecular features identified by each method in the baseline comparison (CCL2 vs. Kyoto) correlate with the infection phenotype across 10 HeLa sub-cell lines (Figure 3c).
    - **Result:** All methods showed modest median correlations ($|\rho| < 0.12$), consistent with distinct molecular origins of these features. However, SECAT interactor ratio provided a significantly different correlation distribution compared to protein abundance ($p = 0.026$, Wilcoxon rank-sum test), indicating that stoichiometric features capture a distinct biological signal not accessible from abundance data.

Together, these analyses quantitatively demonstrate that our network-based methods extract biologically essential and spatially coherent information that is inaccessible from protein abundance analysis alone.

> **R1.3:** As a result, it remains unclear what concrete biological insights can be gained from the study, particularly regarding the specific behavior or phenotypic diversity of the HeLa cell line panel. The main takeaway seems to be that integrating multiple complementary data types provides more information than protein abundance alone—a conclusion that, while reasonable, is conceptually evident from the outset. Overall this leaves a very unfulfilling impression, making it is hard to see the manuscript as a strong candidate for this journal.

Response: We agree that the general concept that "integration is better" alone would not be sufficient. Our revised manuscript provides specific mechanistic insights that abundance data misses, and the new validation framework (Figure 3) empirically demonstrates this.

To move beyond lists of proteins, we have added **Figure 7** ("Molecular landscape of phenotypic divergence between HeLa CCL2 and HeLa Kyoto"), a biological overview schematic that synthesizes the key findings into a coherent narrative. This figure traces the mechanistic connections from upstream signaling (IQGAP2–CDC42, LIMK1, Rho GTPases) through cytoskeletal remodeling (Arp2/3 complex, WIPF1/WIPF2 stoichiometry) to the invasive phenotype (invadopodium formation, ECM degradation), and separately highlights the constitutive-to-immunoproteasome switch and SCF complex reconfiguration. None of these mechanistic insights, which connect specific protein complex stoichiometries to the HeLa CCL2 invasive phenotype, are accessible from abundance data alone.

Crucially, the revised **Figure 5** now provides the analytical bridge between the network diffusion results and these mechanistic conclusions. Figure 5a is a GSEA activation heatmap summarizing Reactome pathway activity across all 64 upstream × downstream layer combinations, revealing which upstream omics layers (e.g., VESPA kinase activity, VIPER TF activity, SECAT metrics) propagate the strongest cross-layer signal onto which biological processes. The new pathway community network (Fig. 5b) then directly maps individual bridging proteins onto the Reactome pathways they connect: two major clusters emerge: a *cytoskeletal remodelling and invasion* cluster linking Signaling by Rho GTPases, Infectious disease and Nervous system development through Arp2/3 subunits (ARPC1B/2/3/5) and WIPF1, and a *protein turnover and immune adaptation* cluster connecting Post-translational protein modification, Disease and Immune System through proteasome subunits (PSMB2, PSMC2, PSMD2/12) and ubiquitin-pathway components. These two themes served as a systematic guide for selecting the mechanistic examples discussed in Figure 6, ensuring that the biological narrative is anchored in a data-driven, pathway-level framework rather than ad hoc protein selection.

We have also expanded the Discussion to include a direct comparison with latent factor approaches such as MOFA, noting that our mechanistic framework "complements latent factor models by constructing context-specific molecular interaction networks from the measured data and mapping diverse molecular profiles onto them, thereby identifying specific functional drivers (e.g., 'active Kinase X') rather than abstract variance factors." Furthermore, we now include a detailed limitations paragraph acknowledging the constraints of our model system and suggesting future directions including CRISPR perturbation-based causal validation.

> **R1.4.1:** The authors observe that "single-state" monomeric and assembled fractions show similar correlations with CNV and mRNA abundance, whereas this relationship diverges for "paired-state" proteins. From this, they conclude that "proteins in assembled conformations are buffered from CNV and mRNA abundance variation compared to their monomers" (p. 4). I find this to be a rather generalized conclusion drawn from Pearson correlation coefficients that are relatively low to begin with, and that differ by only about 0.1. Moreover, multiple layers of regulation—at the levels of RNA, protein stability, post-translational modifications, or protein-protein interactions (or combinations thereof)—could equally explain minor shifts in correlation values. While the authors partly recognize this, this may be interpreted as "buffering" in some specific instances, but alternative regulatory mechanisms may underlie the observations for other proteins. In addition, the protein classes shown in Fig. 2c and d include variable numbers of proteins, raising the concern that modest deviations in smaller datasets (e.g., the top-left panel in Fig. 2c) may disproportionately affect the calculated Pearson correlations. The authors should acknowledge this broader perspective to prevent over-interpretation of the data. Otherwise, the conclusion that "protein complexes buffer against heterogeneity in mRNA abundance" risks leaving readers with an appealing yet insufficiently substantiated message—one that rests largely on a single statistical measure (i.e. Pearson correlation).

Response: We appreciate the detailed critique. We note that the original manuscript reported Spearman's ρ (rank-based correlation), not Pearson correlation coefficients. Spearman's ρ is more robust to outliers and does not assume linearity, partially mitigating the concern about sensitivity to small deviations in low-n subgroups. We have revised the text to acknowledge the limitations of the correlation analysis and to present it more carefully. Specifically:

1. **Paired-state internal control:** The key analytical advance is the comparison of monomer vs. assembled fractions *within the same proteins*. For "paired" proteins detected in both states, the assembled fraction shows significantly lower correlation with CNV ($\rho = 0.198$) than the monomeric fraction ($\rho = 0.322$; $p = 4.92 \times 10^{-4}$, Fisher's z-test). The same holds for mRNA ($\rho_{\text{assembled}} = 0.484$ vs. $\rho_{\text{monomer}} = 0.574$; $p = 1.09 \times 10^{-3}$). Because both fractions originate from the same translational and post-translational environment (the same protein in the same cell), the observed difference isolates the effect of complex assembly from confounding regulatory mechanisms.

2. **Single-state controls:** We now show that "single state" proteins (detected exclusively as monomer or assembled) exhibit similar CNV and mRNA correlations regardless of their state ($\rho_{\text{monomer}} = 0.225$ vs. $\rho_{\text{assembled}} = 0.293$ for CNV; $\rho_{\text{monomer}} = 0.578$ vs. $\rho_{\text{assembled}} = 0.594$ for mRNA). The divergence only appears in "paired" proteins, which is the specific prediction of the buffering hypothesis.

3. **Functional consequence:** We now link this observation to fitness. Paired-state proteins are significantly more essential than single-monomer proteins ($p = 1.35 \times 10^{-29}$, Wilcoxon rank-sum test; Figure 2b; $n_{\text{paired}} = 3{,}439$ vs. $n_{\text{single monomer}} = 654$). This moves the argument from a descriptive correlation difference to a biologically grounded mechanism: cells must buffer these specific proteins, which form the core of essential complexes (ribosomes, proteasomes, spliceosomes), to survive.

4. **Regarding sample sizes:** We now state the protein counts for each category explicitly in the text and figure legend ($n_{\text{paired}} = 3{,}439$; $n_{\text{single assembled}} = 2{,}869$; $n_{\text{single monomer}} = 654$). We acknowledge that the single-monomer group is the smallest, and we report all statistical comparisons with appropriate tests.

R1.4.2: The various analyses presented in the manuscript yield a large number of proteins, interactions and functional categories that are reported to differ between the analyzed cell lines (pages 8-17). However, it is unclear which of these findings are robust or even biologically meaningful. The core issue is that the cell lines differ in unknown ways, leaving no ground truth to assess the validity or performance of the analyses conducted at the different data levels. A more informative approach would have been to use a model system that includes a defined perturbation with a known biological effect—potentially coupled with the question of how the same perturbation elicits context-dependent (i.e., cell line-specific) downstream responses.

Response: We fully agree. As detailed in our response to R1.2, we have directly addressed the "unknown ways" and "no ground truth" critique by adding a validation section with three orthogonal benchmarks (**Figure 3**):

- The **DepMap CRISPR dependency** dataset provides an essentiality ground truth, confirming that SECAT stoichiometric features identify core essential genes (median dependency 0.52) while abundance features do not (median 0.06).
- The **HPA subcellular localization** analysis validates that our top features map to the specific cellular compartments (focal adhesions, actin filaments, plasma membrane) implicated in the invasive phenotype.
- The **Let7 perturbation** serves as a defined perturbation with known biological effect across the same HeLa panel, testing whether our baseline features predict phenotypic response.

Regarding the suggestion to use a defined perturbation with cell-line-specific downstream responses: the Let7 perturbation experiment fulfills exactly this role, as it was applied across the entire cell-line panel, allowing us to test cell-line-specific response prediction. We acknowledge in the Discussion that future work incorporating targeted CRISPR perturbations alongside multi-omics profiling would enable causal validation and extend the framework beyond observational comparisons.

Beyond this global validation, we have added explicit protein-level contrasts to the manuscript demonstrating specific cases where network-based analysis succeeds and abundance fails: proteasome subunit PSMB8 is undetected in the unfractionated mapDIA dataset yet shows a highly significant SECAT interactor ratio change ($p_{\text{adj}} = 8.1 \times 10^{-11}$), while PSMB9 is quantified but not differentially abundant ($\log_2\text{FC} = 0.43$, $\text{FDR} = 0.50$), revealing a constitutional-to-immunoproteasome switch detectable only by SECAT complex stoichiometry (Fig. 6i); the IQGAP2–CDC42 scaffolding complex is differentially assembled yet neither protein changes in unfractionated total abundance (IQGAP2: $\log_2\text{FC} = -0.32$, $\text{FDR} = 0.49$; CDC42: $\log_2\text{FC} = 0.48$, $\text{FDR} = 0.49$; Fig. 6g); and COP9 signalosome subunits are not significantly changed in unfractionated abundance (all $\text{FDR} > 0.1$) while SECAT reveals differential DDB1/2 supercomplex stoichiometries and significant interactor ratio changes across most CSN subunits (Fig. 6h). These specific examples complement the global validation by illustrating the mechanistic insights that abundance analysis alone would miss.

R1.4.3: Several figures contain an overwhelming amount of information, making it difficult to interpret their main message, even when guided by the authors' descriptions or conclusions (e.g., Fig. 3d). Other figures attempt to integrate so many layers of data that it becomes challenging—at least for this reviewer—to grasp the key message, whether at the detailed or overall level (Figs. 4 and 5).

Response: We have taken several steps to improve figure clarity and accessibility:

- We added **Figure 7**, a biological schematic that provides a clear narrative overview of the key mechanistic findings, removing the need to mentally synthesize the complex data figures.
- We added **Box 1** to define all tools and concepts before the reader encounters the data.
- We increased font sizes for axis labels, legends, and annotations across all main and supplemental figures (Figures 2–6 and all Appendix/EV figures).
- We substantially expanded all figure legends to define abbreviations (e.g., NES = Normalized Enrichment Score), explain axes, and describe statistical tests, sample sizes (n), and error bar definitions.
- The original Figure 3d (relative feature importance) has been removed entirely and replaced by a new three-panel validation figure (Figure 3a–c: DepMap dependency, HPA localization, Let7 correlations), which is more interpretable and directly addresses the ground-truth concern.
- We restructured the figure sequence: the validation results (Figure 3) now appear early, immediately after the buffering analysis (Figure 2), so the reader has the ground-truth benchmark in hand before encountering the detailed differential analyses (Figures 4–6).

Reviewer #2

> The manuscript "Complex Assembly and Activity States as Multifaceted Protein Attributes Explaining Phenotypic Variability" by Rosenberger et al. describes a comprehensive systems-level analysis of protein complex dynamics and activity states. As a model system, authors use a well-characterized panel of 14 HeLa cell lines, known for their phenotypic heterogeneity in cell doubling time and susceptibility to *Salmonella* Typhimurium infection. Introducing a multi-omics framework that integrates SEC-SWATH-MS, network inference tools (SECAT, VIPER, VESPA), and network diffusion analysis (TieDIE), authors demonstrate that protein attributes beyond abundance, such as complex assembly and inferred protein activities, provide deeper functional insights into mechanisms underlying molecular phenotypes.
>
> Overall, this is excellent and well-structured study that builds on prior work by the authors and contributes meaningfully to the field of molecular systems biology. The integration of proteomic, transcriptomic, and phosphoproteomic data to infer functional protein states and align elucidated functional dependencies with phenotypes is compelling. I support publication pending minor text revisions.
>
> **R2.1:** The font size in Figures 3, 4, 5, 7 and Supplementary Figures 5-12 is too small and difficult to read. Annotations on Figure 5 panel A appear to be blurred. Increasing the font size for axis labels, legends, and annotations would significantly improve the clarity and accessibility of otherwise excellently presented data.

Response: We have regenerated all mentioned figures with increased font sizes for axis labels, legends, and annotations. Specifically, the base font size was increased across Figures 3–5 (formerly Figures 3, 4, 5, 7 in the original submission) and Appendix Figures S1–S8 (formerly Supplementary Figures 5–12). All panels were re-rendered to ensure readability at printed journal size. We have also reviewed the Figure 5a heatmap annotations to ensure they are no longer blurred.

> **R2.2:** To strengthen the biological impact of the findings, providing or proposing as future direction some experimental validation of a few computational inferences would be beneficial. For example, could the differential activity of LIMK1 or the reconfigured SCF complex (FBXO2) be confirmed in those HeLa cell lines using orthogonal approaches like kinase assay or co-immunoprecipitation?

Response: We agree that validation is crucial. As detailed in our response to R1.2, we have performed a comprehensive **systems-level validation** using three independent datasets: DepMap CRISPR dependency (essentiality), HPA subcellular localization (spatial coherence), and Let7 microRNA perturbation (phenotypic drivers). These results are presented in the new **Figure 3**.

We believe this global validation is more appropriate for a multi-omics methodology paper than validating a single target, as it demonstrates the generalizability of the methods (VESPA, VIPER, SECAT) in distinguishing signal from noise across hundreds of proteins. Nevertheless, we have added specific experimental validation suggestions to the Discussion for future directed studies:

“the predicted differential LIMK1 signaling activity could be confirmed by in vitro kinase assays with cofilin substrates, the inferred SCF complex reconfiguration involving FBXO2 by co-immunoprecipitation and ubiquitination assays, and the WIPF1/WIPF2 stoichiometry shift by live-cell imaging of invadopodium dynamics after targeted knockdown. Similarly, the predicted immunoproteasome switch (Fig. 6i) could be validated by activity-based profiling of proteasome catalytic subunits.”

> **R2.3:** In the section on “Association of protein abundance and protein complex/network dysregulation with phenotypic variability” it would be helpful to briefly reiterate which HeLa cell lines are susceptible to infection and which exhibit faster or slower doubling times. This would help re-orient the reader when interpreting the differential analyses.

Response: We have added a clarification in the Introduction (p. 5) that now explicitly states the phenotypic characteristics: “CCL2 (more invasive and infection-susceptible); Kyoto (faster doubling time).” This orientation is provided before the Results begin, so the reader has the phenotypic context in hand when interpreting the differential analyses.

Reviewer #3

The manuscript submitted by Rosenberger et al describes a novel approach of omic data integration by which transcriptome, proteome, phosphoproteome, and interactome data are used to infer protein activity states. These protein activity states along with protein complex and network states are then used to generate hypotheses about molecular mechanisms driving two different phenotypes, doubling time and infection susceptibility, of two HeLa cell lines. Most of the data has been generated before while protein complex measurements were done as part of this study. They authors find that inferences of complex and network dysregulation are more informative on potential molecular mechanisms and connecting genotype to phenotype than protein abundances alone. The authors use their novel multiomic data integration approach to show that protein complex stoichiometry is a cellular buffering mechanisms against CNVs and transcriptional variability.

R3.1: The manuscript addresses an important problem in biology in showing that genotype-to-phenotype relationships can only be understood and predicted if we incorporate the molecular interaction networks that mediate these relationships. It is impossible to predict phenotypes from the cellular parts list, the abundances of transcripts and proteins, alone. To make this point, the authors use an interesting model system of two divergent HeLa cell lines that have been extensively profiled in previous studies. However, my main critique point to this manuscript is that at many places it is not understandable. That is because a lot of vague technical terms are used that are not defined. A lot of software is used that is highly specialized (unknown to most readers including the reviewer) and not properly introduced, and as a consequence it remains largely unclear how authors came to the conclusions that they state in the manuscript.

Response: We apologize for the lack of clarity in the original manuscript. We have significantly revised the text to define all specialized terminology early and systematically. The most important addition is **Box 1** ("Integrated Multi-Omics Toolkit for Inferring Protein Functional States"), placed in the Introduction before the Results section. Box 1 explicitly defines:

- **"Protein Activity":** The functional output of a protein, defined by context as catalytic (signaling), regulatory (transcriptional), or structural (complex assembly).
- **VIPER:** Infers Gene Regulatory Activity (TF activity) from transcriptomic data by enrichment analysis of TF regulon targets.
- **VESPA:** Infers Signaling Activity (kinase/phosphatase activity) from phosphoproteomic data using kinase-substrate relationship enrichment.
- **SECAT:** Infers Protein Complex Assembly State from SEC-SWATH-MS co-elution profiles, quantifying complex stoichiometry and distinguishing monomeric from assembled pools.

- **diffuStats:** Network diffusion analysis integrating layers via random-walk propagation over PPI and gene regulatory networks.

We have also removed the DeMAND, STELLA, and TieDIE analyses from the manuscript entirely, as these tools were not central to the core findings and added unnecessary complexity. TieDIE has been replaced by diffuStats, which provides a more flexible network diffusion framework. The revised manuscript focuses on three clearly defined analytical layers (VIPER, VESPA, SECAT) integrated through network diffusion (diffuStats). We have performed a systematic pass through the entire manuscript to replace jargon with defined terms or provide definitions on first use.

> **R3.2 - R3.5:** For example: What are the tools SEC-SWATH-MS, VESPA, VIPER, SECAT, STELLA, DeMAND, TieDIE do, conceptually? What do you mean by protein activity? It seems from the manuscript that at different places this means different things? Here is an example of a sentence that is unclear (page 11): "Comparing the sets of differentially active proteins between the three functional levels tested by the algorithms (p. adj < 0.05), illustrates the heterogeneity of protein function, as well as potential confounding technical biases between the different types of analysis." What are the three functional levels? What are the tested algorithms? What do you mean by heterogeneity of protein function? Which different types of analyses? Which technical biases? Here is another example (page 13): "Therefore, our goal was to assess whether the context-specific PPI networks and SECAT-informed quantitative features at the different levels of information provided by SECAT could be used to elucidate molecular mechanisms differentiating CCL2 and Kyoto HeLa cells." What are the SECAT-informed quantitative features? What are the different levels of information provided by SECAT? I encourage the authors to check the entire manuscript for these kinds of overly general and cryptic statements and improve the clarity of the manuscript.

Response: We have addressed each of these clarity issues through comprehensive revisions:

- **Tool definitions:** SEC-SWATH-MS, VESPA, VIPER, SECAT, and diffuStats are now described both conceptually in **Box 1** and technically in the Introduction paragraphs. We have removed DeMAND, STELLA, and TieDIE from the manuscript entirely, as they were not central to the core findings and added confusion. TieDIE has been replaced by diffuStats, which implements a more flexible random-walk-based network propagation framework. The revised analysis uses only three clearly defined tools (VIPER, VESPA, SECAT) with network diffusion (diffuStats) as integration layer.

- **"Protein Activity":** Now explicitly defined in Box 1 as the functional output of a protein, whether catalytic (signaling, inferred by VESPA), regulatory (transcriptional, inferred by VIPER), or structural (complex assembly, measured by SECAT). This definition is used consistently throughout.

- **"Three functional levels" / "tested algorithms":** The revised text now explicitly names these as (i) signaling activity (VESPA, 549 kinases/phosphatases), (ii) transcription factor

activity (VIPER, 6,156 TF/co-TF), and (iii) protein complex dynamics (SECAT, 6 quantitative metrics per protein).

- **"SECAT-informed quantitative features" / "different levels of information":** We now define SECAT's six quantitative metrics on first mention: *total abundance*, *assembled abundance*, *monomer abundance*, *complex abundance*, *interactor abundance*, and *interactor ratio* (stoichiometric ratio relative to interaction partners).

- **"Heterogeneity of protein function":** The revised text (Figure EV3 discussion) now explicitly states that the three methods identify largely non-overlapping protein sets, with minimal overlap found between signaling (VESPA) and structural (SECAT) differentially active proteins (4 proteins, 0.3%), and 13.5% overlap between SECAT and VIPER. This reflects the distinct functional dimensions each method captures: VESPA nominates upstream kinase/phosphatase enzymes from phosphoproteomic data, while SECAT identifies downstream structural targets from co-fractionation profiles. As shown in Figure EV3d, the indirect connection between phosphorylation and complex dynamics is present at the substrate level (e.g., DDB2), but the enzyme identities reported by VESPA do not overlap with the structural protein identities reported by SECAT.

- We have performed a full systematic pass through the manuscript to identify and clarify all remaining instances of vague or overly general language.

R3.6: Other major points: The introduction lacks more information on the previous study that generated the molecular signatures of the HeLa cell lines and the findings made since the authors refer to it at multiple places in the manuscript. It would also be nice to have more information provided as to why these profiled HeLa cell lines are a good model system for this study.

Response: We have expanded the Introduction to better summarize the 2019 *Nature Biotechnology* study (Liu et al.) and to explain why these HeLa cell lines are a uniquely suited model system. Specifically, we now describe:

- The study profiled "14 HeLa samples gathered from 13 laboratories" with "matched genomic, transcriptomic, proteomic, and phenotypic (cell doubling time, *Salmonella* infection susceptibility) data."

- The key finding was "the robust correlation between 'proteotypes', i.e., the contextual state of the proteome, and cell phenotypes, particularly cell doubling time and susceptibility to *Salmonella* Typhimurium infection (CCL2, more invasive and infection-susceptible; Kyoto, faster doubling time)."

- We frame the cell line panel as a "natural perturbation" system: "genetic drift has generated a diverse landscape of proteotypes and phenotypes, analogous to the clonal heterogeneity observed in solid tumors, providing an ideal testbed for evaluating whether inferred protein activities correlate better with cell function than simple abundance."

- We state the three hypotheses from the prior study that the current work tests: (i) protein complex stoichiometry buffers CNV/transcriptional variation, (ii) protein function is more closely related to phenotype than protein abundance, and (iii) multi-omics data provide complementary information to connect molecular profiles to phenotypes.

R3.7: The manuscript lacks references to previous work on multiomic data integration approaches, i.e. such as MOFA. What are the advantages and disadvantages of the newly proposed versus previously existing approaches?

Response: We have addressed this at two points in the revised manuscript:

1. **Introduction (p. 4):** We now explicitly compare with MOFA: "While latent factor models such as MOFA can identify shared variance across omics layers, they primarily capture associational patterns and do not resolve the mechanistic protein-level processes, such as complex assembly or kinase signaling, that drive phenotypic variation." This positions the knowledge gap our approach fills.

2. **Discussion:** We provide a more detailed comparison: "This mechanistic approach complements latent factor models (e.g., MOFA) by constructing context-specific molecular interaction networks from the measured data and mapping diverse molecular profiles onto them, thereby identifying specific functional drivers (e.g., 'active Kinase X') rather than abstract variance factors." We also reference footprint-based functional analysis (Dugourd et al., 2019) as a related conceptual approach.

In summary, the key distinction is: MOFA identifies *which omics layers co-vary* (dimensionality reduction), whereas our approach identifies *which specific molecular mechanisms drive the observed variation* (mechanistic interpretation via context-specific molecular interaction networks constructed from the measured data).

R3.8: One of the key findings reported in this manuscript is that protein complex assemblies enable buffering against protein copy number variation. This to the best of my understanding is not new. Other studies have reported that orphan complex subunits, so those that cannot be incorporated into protein complex assemblies because there is not enough of the other complex subunits, are being rapidly degraded as a consequence. How is your analysis and how are your findings different from this previous work (i.e. reviewed in these articles: https://www.cell.com/trends/cell-biology/fulltext/S0962-8924(22)00038-1, https://pmc.ncbi.nlm.nih.gov/articles/PMC6624128)?

Response: We agree that the *concept* of orphan subunit degradation buffering is well established (as reviewed in the cited articles by Taggart & Bhatt, 2020 and Pla-Prats & Bhatt, 2022). Our revised manuscript now explicitly cites these reviews and the underlying quality control mechanisms. However, previous studies inferred buffering from (i) global correlation mismatches between total mRNA and total protein levels, or (ii) pulsed-SILAC measurements showing elevated degradation rates of excess subunits (e.g., Salovska et al., 2020). Our study provides a fundamentally different type of evidence: **direct physical separation of monomeric and assembled fractions** of the same proteins via SEC-SWATH-MS. This allows us to directly observe that, within the same cell, the assembled pool is insulated from CNV/mRNA fluctuations while the monomeric pool is not.

The revised text now states (Results, "Quantitative effects" section): "Multiple layers of regulation contribute to maintaining protein complex stoichiometry: coordinated translational control can tune subunit production rates (Taggart *et al*, 2020), while orphaned subunits that fail to assemble are targeted for degradation by the ubiquitin-proteasome system (Juszkiewicz & Hegde, 2018; Pla-Prats & Thomä, 2022). The relative contributions of these mechanisms depend on the biological context: for instance, gain-type aneuploidy is primarily buffered by accelerated degradation of excess subunits, whereas loss-type aneuploidy seems to engage selective upregulation of protein synthesis (Di *et al*, 2025), establishing a multi-layered buffering system against gene dosage variation (Liu *et al*, 2019, 2016). However, previous studies largely inferred this buffering from global correlation analyses, pulsed-SILAC measurements, or bulk protein pools (Salovska *et al*, 2020). Here, we provide direct physical evidence for the complex-based buffering hypothesis by resolving the distinct behavior of monomeric versus assembled fractions of the same proteins."

Additionally, as described in R1.1, we now link this buffering to the preservation of essential cellular machinery ($p = 1.35 \times 10^{-29}$; Figure 2b), adding a functional evolutionary dimension that is absent from prior work.

R3.9: On page 7 the authors describe how they developed and used STELLA to generate cell line-specific PPI networks by integrating publicly available datasets on PPIs with protein abundance profiles obtained from this work. They then use the tool DeMAND to identify proteins with dysregulated interactions (what do you precisely mean by this? interactions that are absent or present in the two cell lines?) to quantify protein complex and network dysregulation (what do you mean by

dysregulation more precisely)? If PPI dysregulation is inferred from protein abundance differences in the two cell lines, then it remains unclear how the authors can later on conclude from this data that PPI dysregulation is more powerful than protein abundances in understanding molecular mechanisms. I agree that PPI data is key in studying molecular mechanisms but protein abundance is also key for inferring differential PPIs between cellular states in their approach, right? So, it is misleading how the authors undermine the importance of protein abundance in their study.

Response: This is an important distinction and we thank the reviewer for raising it. First, a clarification on methodology: the revised manuscript no longer uses STELLA or DeMAND, which have been replaced by the more streamlined SECAT-only workflow. This simplification removes two layers of software complexity without affecting the biological conclusions, because SECAT's co-elution-based analysis provides a more direct and interpretable readout of protein complex dynamics than the abundance-inferred networks produced by STELLA/DeMAND. TieDIE has been replaced by diffuStats, a more flexible random-walk-based network diffusion framework. SECAT does not simply infer differential PPIs from protein abundance differences. Instead, SECAT analyzes the **co-elution profiles** of interacting proteins across 70 SEC fractions, i.e., the physical separation of intact complexes by size. The PPI inference is based on the *correlation structure* (co-elution pattern) between interacting partners, not on their individual abundances. A protein can be highly abundant yet show altered stoichiometry (interactor ratio) relative to its partners, or vice versa.

Our new DepMap validation (Figure 3a) provides direct empirical evidence for this distinction: SECAT's "interactor ratio" metric (which captures stoichiometric changes) identifies essential genes with a median dependency probability of **0.52**, while simple differential "abundance" identifies genes with a median dependency of only **0.06**, an 8.7-fold difference ($p = 6.4 \times 10^{-5}$, Wilcoxon rank-sum test). This proves that the network transformation extracts orthogonal, biologically critical information that is inaccessible from abundance data alone.

We do not undermine the importance of protein abundance. Rather, we demonstrate that abundance is a necessary but insufficient proxy for function, and that the transformation of abundance data through context-specific molecular interaction networks (PPI co-elution, kinase-substrate relationships, TF regulons) reveals functional layers that abundance measurements alone cannot capture. We have revised the text to state this distinction more carefully.

R3.10: Furthermore, the authors then find that these PPI, complex and network dysregulations are not more powerful than the other data modalities in identifying "molecular determinants that are linked to the phenotypes" (what do you actually precisely mean by molecular determinants?). To still show that PPI, complex and network dysregulation are more powerful, the authors use GO cellular component (CC) annotation enrichments. I wonder to which extent this gives a favour to these inferred data modalities because the GO CC terms are precisely describing protein complexes. How would the analysis look like when using GO biological process or molecular function terms instead?

Response: The reviewer raises a valid methodological point. In the revised manuscript, we have replaced the GO Cellular Component (GO:CC) analysis with a more rigorous validation using the **Human Protein Atlas (HPA)** subcellular localization annotations, analyzed via fast gene set enrichment analysis (fgsea, 10,000 permutations; Figure 3b). This analysis uses experimentally determined protein localizations from immunofluorescence imaging (the HPA "Main location" field), not GO annotation terms, and therefore provides independent validation.

The key finding is that **SECAT complex abundance** features map to five significantly enriched compartments (BH-adj. $p < 0.05$): focal adhesion sites, actin filaments, plasma membrane, vesicles, and mitochondria. These are precisely the subcellular structures implicated in the mechanistic findings of our differential analysis (cytoskeletal remodeling, ECM degradation, immunoproteasome). In contrast, **protein abundance** features map only to nucleolar proteins, an unrelated compartment.

While it is true that SECAT is designed to detect changes at the complex/interaction level, the fact that these changes independently map to the specific compartments driving the invasive phenotype validates the biological relevance of this approach. The HPA analysis does not inherently favour complex-level features (unlike GO:CC), as it captures all protein localizations regardless of complex membership.

> **R3.11:** Towards the end of the results section the authors describe observations from the integrative analyses that might underlie the different phenotypes observed in both HeLa cell lines. However, I miss figures and analyses that show how these findings could not have been made by using protein abundance profiles alone.

Response: The new validation framework in **Figure 3** directly addresses this concern with side-by-side comparisons across all methods:

- **DepMap essentiality (Figure 3a):** The top 100 proteins ranked by each method are compared head-to-head for their CRISPR gene dependency scores. SECAT interactor ratio features map to highly essential genes (median dependency 0.52), while differentially abundant proteins do *not* (median 0.06; $p = 6.4 \times 10^{-5}$). This directly shows that abundance alone fails to identify the core essential machinery that complex stoichiometry analysis captures.

- **HPA spatial enrichment (Figure 3b):** SECAT complex abundance identifies 5 biologically relevant subcellular compartments (focal adhesions, actin filaments, plasma membrane, vesicles, mitochondria), while protein abundance identifies only nucleolar proteins. This demonstrates that abundance-derived features do not localize to the invasion-relevant structures.

- **Complementarity analysis (Figure EV3):** The revised manuscript shows that VESPA (signaling) and SECAT (structural) share 0% overlap in identified proteins, while there is 15.3% overlap between SECAT and VIPER. The 55-protein VIPER–SECAT intersection includes proteins like CDH2 and CTNNB1 (linking WNT5A regulatory activity to adherens

junction complex dynamics), demonstrating cross-layer convergence that neither method captures alone.

Together, these analyses provide quantitative evidence that each modality captures distinct and complementary biology that protein abundance analysis misses.

In addition to these global comparisons, we have added explicit protein-level contrasts to the Results text. For instance, the IQGAP2–CDC42 scaffolding complex is differentially assembled between cell lines by SECAT, yet neither protein shows a significant change in unfractionated total abundance (IQGAP2: $\log_2 FC = -0.32$, $FDR = 0.49$; CDC42: $\log_2 FC = 0.48$, $FDR = 0.49$; Fig. 6g); COP9 signalosome subunits are not significantly changed in unfractionated abundance (all $FDR > 0.1$) while SECAT reveals differential DDB1/2 supercomplex stoichiometries and significant interactor ratio changes across most CSN subunits (Fig. 6h); and the proteasome undergoes a constitutional-to-immunoproteasome switch: PSMB8 is undetected in the unfractionated mapDIA dataset yet shows a SECAT interactor ratio $p_{adj} = 8.1 \times 10^{-11}$ (Fig. 6i). These examples directly demonstrate that the mechanistic conclusions in Figure 6 could not have been reached from protein abundance profiles alone.

R3.12: The current figures also remain largely unclear because the figure legends lack sufficient information to understand what readers are looking at. For example, figure 6, what are upstream and downstream layers? What is the quantification on the x-axis (numbers ranging from 0-5 labelled Significant subpathways)? Many plots have NES as x-axis label but I couldn't find a place in the manuscript where the abbreviation NES is explained. I also don't understand figure 3d. What am I looking at? How was the relative feature importance determined and how can I see that complex and network dysregulation related the different gene sets better with the two phenotypes? What are the correlation coefficients?

Response: We have substantially expanded all figure legends to address these specific issues:

- **NES:** Now defined on first use in the text and in figure legends as "Normalized Enrichment Score" (e.g., Figure 3b legend: "Normalized enrichment scores (NES; fgsea, 10,000 permutations)").

- **Upstream and downstream layers:** Explicitly defined in the Results text and Figure 5 legend. Upstream layers are the eight molecular features used as diffusion seeds (VIPER TF activity, VESPA kinase activity, and six SECAT metrics). Downstream layers are the same eight features used as output targets. The diffuStats z-score method propagates upstream signals across the PPI/GRN network to assess effects on downstream layers, yielding 64 pairwise combinations.

- **Figure 5a (formerly Figure 6):** The legend now specifies: "Cell colour represents a combined score, defined as the product of the fraction of significant L1 subpathways (BH-adj. $p < 0.05$) and the mean leading-edge fraction across those significant pathways, capped at the 95th percentile." Columns are "grouped by upstream omics

layer (eight blocks)” and rows are “Reactome top-level (L0) categories (hierarchically clustered).”

- **Figure 3 (validation, formerly Figure 3d):** The revised Figure 3 now presents three clearly separated panels: (a) DepMap dependency score distributions per method, (b) HPA enrichment NES per compartment, and (c) Let7 correlation distributions per method. Each panel includes the statistical test used, sample sizes, and axis definitions. The legend for Figure 3a specifies: “median gene dependency probability” and the 8.7-fold enrichment.

- **Figure 4 legends (boxplots):** Now explicitly define “centre line, median; box limits, first and third quartiles; whiskers, $1.5 \times$ IQR; points, individual leading-edge target NES values.”

- **All figures:** Font sizes have been increased for axis labels, legends, and annotations as noted in R2.1.

6th Jun 2026

Manuscript Number: MSB-2025-13379R
Title: Complex Assembly and Activity States as Multifaceted Protein Attributes Explaining Phenotypic Variability
Author: George Rosenberger
Peng Xue
Isabell Bludau
Claudia Martelli
Evan Williams
Ben Collins
Andrea Califano
Yansheng Liu
Ruedi Aebersold

Dear Dr. Rosenberger,

Thank you for sending us your revised manuscript. We have now heard back from the two reviewers who were asked to re-evaluate your study. As you will see, both reviewers are satisfied with the revisions and consider the manuscript to have improved substantially during the revision process.

Before we can formally accept your manuscript, we would ask you to address the following editorial-level issues:

1. Please remove "Author contribution" section from the manuscript file.

2. "Disclosure statement and competing interests" should be renamed to "Disclosure and Competing Interests Statement".

3. Please move the declaration of AI usage to the Methods section.

4. When submitting your revised manuscript, please do not include the Reagents and Tools Table in the Methods section of the manuscript but upload it as a separate file choosing the file type "Reagent Table". Please download and fill our Reagents and Tools Table template (.docx), which you can find in our author guidelines: https://www.embopress.org/page/journal/17444292/authorguide#structuredmethods.

5. Please remove the authors' names from the Appendix.

6. Please move the figure legends to the end of the manuscript file.

7. Data availability: Please note that this section applies only to primary datasets generated in this study. Published datasets should be cited using standard "data citations" in both the manuscript text and the reference list.

8. Data citation formatting guideline: Our journal encourages inclusion of *data citations in the reference list* to directly cite datasets that were re-used and obtained from public databases. Data citations in the article text are distinct from normal bibliographical citations and should directly link to the database records from which the data can be accessed. In the main text, data citations are formatted as follows: "Data ref: Smith et al, 2001". In the Reference list, data citations must be labeled with "[DATASET]". A data reference must provide the database name, accession number/identifiers and a resolvable link to the landing page from which the data can be accessed at the end of the reference. Further instructions are available at < https://link.springer.com/journal/44320/submission-guidelines#cms-Reference-guidelines >.

9.Please add a callout to Box 1.

10. EV datasets should be provided as EV tables should be uploaded as separate Excel (.xlsx) files, with one file per table. Each file should include a separate sheet labeled 'Legend' containing the corresponding table legend.

11. We have updated the dimension requirements for the visual abstract. Please provide an updated version in PNG format with exact dimensions of 550 px (width) × 300 px (height).

12. Please remove the synopsis text from the manuscript file and submit it as a separate Word document.

13. Please indicate the statistical test used for data analysis in the legends of figures 4A,B,C,D; 5A; EV-2G; EV-3A-D.

14. The manuscript sections should be in the following order: Title page - Abstract - Introduction - Results - Discussion -

Methods -Data Availability - Acknowledgments - Disclosure and Competing Interests Statement - References - Figure Legends - Expanded View Figure Legends.

Use the following link to submit your revised paper:

https://msb.msubmit.net/cgi-bin/main.plex

As a matter of course, please make sure that you have correctly followed the instructions for authors as given on the submission website.

Thank you for submitting this interesting paper to Molecular Systems Biology.

Kind regards,
Jingyi

Jingyi Hou, PhD
Senior Editor
Molecular Systems Biology

*** PLEASE NOTE *** As part of the EMBO Press transparent editorial process initiative (see our Editorial at https://dx.doi.org/10.1038/msb.2010.72 , Molecular Systems Biology will publish online a Review Process File to accompany accepted manuscripts. When preparing your letter of response, please be aware that in the event of acceptance, your cover letter/point-by-point document will be included as part of this File, which will be available to the scientific community. More information about this initiative is available in our Instructions to Authors. If you have any questions about this initiative, please contact the editorial office (msb@embo.org).

Reviewer #2:

The authors satisfactory addressed all my concerns and I therefore am happy to accept their paper in its current form.

Reviewer #3:

The authors have done a huge effort to address my concerns. The manuscript has substantially improved in clarity and I also very much appreciate the clarity and comprehensiveness of the response letter. I have no further requests.

All editorial and formatting issues were resolved by the authors.

11th Jun 2026
Manuscript number: MSB-2025-13379RR
Title: Complex Assembly and Activity States as Multifaceted Protein Attributes Explaining Phenotypic Variability

Dear Dr. Rosenberger,

Thank you again for sending us your revised manuscript. We are now satisfied with the modifications made and I am pleased to inform you that your paper has been accepted for publication.

Your manuscript will be processed for publication by EMBO Press. It will be copy edited and you will receive page proofs prior to publication. Please note that you will be contacted by Springer Nature Author Services to complete licensing and payment information.

You may qualify for financial assistance for your publication charges - either via a Springer Nature fully open access agreement or an EMBO initiative. Check your eligibility: https://link.springer.com/journal/44320/how-to-publish-with-us

Should you be planning a Press Release on your article, please notify the production team via this form in order to coordinate publication and release dates: https://forms.cloud.microsoft/e/eH4A8JVP6g

If you have any questions, please do not hesitate to contact the Editorial Office. Thank you for your contribution to Molecular Systems Biology.

Kind regards,
Jingyi

Jingyi Hou, PhD
Senior Editor
Molecular Systems Biology

>>> Please note that it is Molecular Systems Biology policy for the transcript of the editorial process (containing referee reports and your response letter) to be published as an online supplement to each paper. If you do NOT want this, you will need to inform the Editorial Office via email immediately. More information is available here: https://link.springer.com/partners/embo-press/editorial-policies#Peer%20review

EMBO Press Author Checklist

Corresponding Author Name: George Rosenberger, Yansheng Liu, Ruedi Aebersold	
Journal Submitted to: Molecular Systems Biology	
Manuscript Number: MSB-2025-13379	

USEFUL LINKS FOR COMPLETING THIS FORM
The EMBO Journal - Author Guidelines
EMBO Reports - Author Guidelines
Molecular Systems Biology - Author Guidelines
EMBO Molecular Medicine - Author Guidelines

Reporting Checklist for Life Science Articles (updated January
This checklist is adapted from Materials Design Analysis Reporting (MDAR) Checklist for Authors. MDAR establishes a minimum set of requirements in transparent reporting in the life sciences (see Statement of Task: 10.31222/osf.io/9sm4x). Please follow the journal's guidelines in preparing your
Please note that a copy of this checklist will be published alongside your article.

Abridged guidelines for figures
1. Data
The data shown in figures should satisfy the following conditions:
- the data were obtained and processed according to the field's best practice and are presented to reflect the results of the experiments in an accurate and unbiased manner.
- ideally, figure panels should include only measurements that are directly comparable to each other and obtained with the same assay.
- plots include clearly labeled error bars for independent experiments and sample sizes. Unless justified, error bars should not be shown for technical
- if n<5, the individual data points from each experiment should be plotted. Any statistical test employed should be justified.
- Source Data should be included to report the data underlying figures according to the guidelines set out in the authorship guidelines on Data

2. Captions
Each figure caption should contain the following information, for each panel where they are relevant:
- a specification of the experimental system investigated (eg cell line, species name).
- the assay(s) and method(s) used to carry out the reported observations and measurements.
- an explicit mention of the biological and chemical entity(ies) that are being measured.
- an explicit mention of the biological and chemical entity(ies) that are altered/varied/perturbed in a controlled manner.
- the exact sample size (n) for each experimental group/condition, given as a number, not a range;
- a description of the sample collection allowing the reader to understand whether the samples represent technical or biological replicates (including how many animals, litters, cultures, etc.).
- a statement of how many times the experiment shown was independently replicated in the laboratory.
- definitions of statistical methods and measures:
  - common tests, such as t-test (please specify whether paired vs. unpaired), simple χ2 tests, Wilcoxon and Mann-Whitney tests, can be unambiguously identified by name only, but more complex techniques should be described in the methods section;
  - are tests one-sided or two-sided?
  - are there adjustments for multiple comparisons?
  - exact statistical test results, e.g., P values = x but not P values < x;
  - definition of 'center values' as median or average;
  - definition of error bars as s.d. or s.e.m.

Please complete ALL of the questions below.
Select "Not Applicable" only when the requested information is not relevant for your study.

Materials

Newly Created Materials	Information included in the manuscript?	In which section is the information available? (Reagents and Tools Table, Materials and Methods, Figures, Data Availability Section)
New materials and reagents need to be available; do any restrictions apply?	Not Applicable	Data & Source Code Availability Section

Antibodies	Information included in the manuscript?	In which section is the information available? (Reagents and Tools Table, Materials and Methods, Figures, Data Availability Section)
For **antibodies** provide the following information:<br>- Commercial antibodies: RRID (if possible) or supplier name, catalogue number and or/clone number<br>- Non-commercial: RRID or citation	Not Applicable	

DNA and RNA sequences	Information included in the manuscript?	In which section is the information available? (Reagents and Tools Table, Materials and Methods, Figures, Data Availability Section)
**Short novel DNA or RNA including primers, probes**: provide the sequences.	Not Applicable	

Cell materials	Information included in the manuscript?	In which section is the information available? (Reagents and Tools Table, Materials and Methods, Figures, Data Availability Section)
**Cell lines:** Provide species information, strain. Provide accession number in repository **OR** supplier name, catalog number, clone number, and/**OR** RRID.	Yes	Reagents and Tools Table
**Primary cultures:** Provide species, strain, sex of origin, genetic modification status.	Not Applicable	
Report if the cell lines were recently **authenticated** (e.g., by STR profiling) and tested for mycoplasma contamination.	Yes	

Experimental animals	Information included in the manuscript?	In which section is the information available? (Reagents and Tools Table, Materials and Methods, Figures, Data Availability Section)
**Laboratory animals or Model organisms:** Provide species, strain, sex, age, genetic modification status. Provide accession number in repository **OR** supplier name, catalog number, clone number, **OR** RRID.	Not Applicable	
**Animal observed in or captured from the field:** Provide species, sex, and age where possible.	Not Applicable	
Please detail **housing and husbandry conditions**.	Not Applicable	

Plants and microbes	Information included in the manuscript?	In which section is the information available? (Reagents and Tools Table, Materials and Methods, Figures, Data Availability Section)
**Plants:** provide species and strain, ecotype and cultivar where relevant, unique accession number if available, and source (including location for collected wild specimens).	Not Applicable	
**Microbes:** provide species and strain, unique accession number if available, and source.	Not Applicable	

Human research participants	Information included in the manuscript?	In which section is the information available? (Reagents and Tools Table, Materials and Methods, Figures, Data Availability Section)
If collected and within the bounds of privacy constraints report on age, sex and gender or ethnicity for all study participants.	Not Applicable	

Core facilities	Information included in the manuscript?	In which section is the information available? (Reagents and Tools Table, Materials and Methods, Figures, Data Availability Section)
If your work benefited from core facilities, was their service mentioned in the acknowledgments section?	Yes	Acknowledgments

Design

Study protocol	Information included in the manuscript?	In which section is the information available? (Reagents and Tools Table, Materials and Methods, Figures, Data Availability Section)
If study protocol has been **pre-registered, provide DOI in the manuscript**. For clinical trials, provide the trial registration number **OR** cite DOI.	Not Applicable	
Report the **clinical trial registration number** (at ClinicalTrials.gov or equivalent), where applicable.	Not Applicable	

Laboratory protocol	Information included in the manuscript?	In which section is the information available? (Reagents and Tools Table, Materials and Methods, Figures, Data Availability Section)
Provide DOI OR other citation details if **external detailed step-by-step protocols** are available.	Yes	Materials and Methods

Experimental study design and statistics	Information included in the manuscript?	In which section is the information available? (Reagents and Tools Table, Materials and Methods, Figures, Data Availability Section)
Include a statement about **sample size** estimate even if no statistical methods were used.	Yes	Results, Figures, Extended Figures
Were any steps taken to minimize the effects of subjective bias when allocating animals/samples to treatment (e.g. **randomization procedure**)? If yes, have they been described?	Not Applicable	
Include a statement about **blinding** even if no blinding was done.	Not Applicable	
Describe **inclusion/exclusion criteria** if samples or animals were excluded from the analysis. Were the criteria pre-established?<br><br>If sample or data points were omitted from analysis, report if this was due to attrition or intentional exclusion and provide justification.	Not Applicable	
For every figure, are **statistical tests** justified as appropriate? Do the data meet the assumptions of the tests (e.g., normal distribution)? Describe any methods used to assess it. Is there an estimate of variation within each group of data? Is the variance similar between the groups that are being statistically compared?	Yes	Results, Figures, Extended Figures

Sample definition and in-laboratory replication	Information included in the manuscript?	In which section is the information available? (Reagents and Tools Table, Materials and Methods, Figures, Data Availability Section)
In the figure legends: state number of times the experiment was **replicated** in laboratory.	Yes	Figures
In the figure legends: define whether data describe **technical or biological replicates**.	Yes	Figures

Ethics

Ethics	Information included in the manuscript?	In which section is the information available? (Reagents and Tools Table, Materials and Methods, Figures, Data Availability Section)
Studies involving **human participants**: State details of **authority granting ethics approval** (IRB or equivalent committee(s), provide reference number for approval.	Not Applicable	
Studies involving **human participants**: Include a statement confirming that **informed consent** was obtained from all subjects and that the experiments conformed to the principles set out in the WMA Declaration of Helsinki and the Department of Health and Human Services Belmont Report.	Not Applicable	
Studies involving **human participants:** For publication of **patient photos**, include a statement confirming that consent to publish was obtained.	Not Applicable	
Studies involving experimental **animals**: State details of **authority granting ethics approval** (IRB or equivalent committee(s), provide reference number for approval. Include a statement of compliance with ethical regulations.	Not Applicable	
Studies involving **specimen and field samples:** State if relevant **permits** obtained, provide details of authority approving study; if none were required, explain why.	Not Applicable	

Dual Use Research of Concern (DURC)	Information included in the manuscript?	In which section is the information available? (Reagents and Tools Table, Materials and Methods, Figures, Data Availability Section)
Could your study fall under dual use research restrictions? Please check biosecurity documents and list of **select agents and toxins** (CDC): https://www.selectagents.gov/sat/list.htm	Not Applicable	
If you used a select agent, is the security level of the lab appropriate and reported in the manuscript?	Not Applicable	
If a study is subject to dual use research of concern regulations, is the name of the **authority granting approval and reference number** for the regulatory approval provided in the manuscript?	Not Applicable	

Reporting

The MDAR framework recommends adoption of discipline-specific guidelines, established and endorsed through community initiatives. Journals have their own policy about requiring specific guidelines and recommendations to complement MDAR.

Adherence to community standards	Information included in the manuscript?	In which section is the information available? (Reagents and Tools Table, Materials and Methods, Figures, Data Availability Section)
State if relevant guidelines or checklists (e.g., **ICMJE, MIBBI, ARRIVE, PRISMA**) have been followed or provided.	Not Applicable	
For **tumor marker prognostic studies,** we recommend that you follow the **REMARK** reporting guidelines (see link list at top right). See author guidelines, under 'Reporting Guidelines'. Please confirm you have followed these guidelines.	Not Applicable	
For **phase II and III randomized controlled trials**, please refer to the **CONSORT** flow diagram (see link list at top right) and submit the CONSORT checklist (see link list at top right) with your submission. See author guidelines, under 'Reporting Guidelines'. Please confirm you have submitted this list.	Not Applicable	

Data Availability

Data availability	Information included in the manuscript?	In which section is the information available? (Reagents and Tools Table, Materials and Methods, Figures, Data Availability Section)
Have **primary datasets** been deposited according to the journal's guidelines (see 'Data Deposition' section) and the respective accession numbers provided in the Data Availability Section?	Yes	Data Availability Section
Were **human clinical and genomic datasets** deposited in a public access-controlled repository in accordance to ethical obligations to the patients and to the applicable consent agreement?	Not Applicable	
Are **computational models** that are central and integral to a study available without restrictions in a machine-readable form? Were the relevant accession numbers or links provided?	Yes	Data Availability Section
If publicly available data were reused, provide the respective **data citations in the reference list**.	Yes	Data Availability Section
